# Regioselective Simmons–Smith-type cyclopropanations of polyalkenes enabled by transition metal catalysis†
†Electronic supplementary information (ESI) available: Experimental procedures and characterization data. CCDC 1584851. For ESI and crystallographic data in CIF or other electronic format see DOI: 10.1039/c7sc04861k


**DOI:** 10.1039/c7sc04861k

**Published:** 2018-01-02

**Authors:** Jacob Werth, Christopher Uyeda

**Affiliations:** a Department of Chemistry , Purdue University , West Lafayette , IN 47907 , USA . Email: cuyeda@purdue.edu

## Abstract

A [^*i*–Pr^PDI]CoBr_2_ complex (PDI = pyridine-diimine) catalyzes Simmons–Smith-type reductive cyclopropanation reactions using CH_2_Br_2_ in combination with Zn. In contrast to its non-catalytic variant, the cobalt-catalyzed cyclopropanation is capable of discriminating between alkenes of similar electronic properties based on their substitution patterns: monosubstituted > 1,1-disubstituted > (*Z*)-1,2-disubstituted > (*E*)-1,2-disubstituted > trisubstituted. This property enables synthetically useful yields to be achieved for the monocyclopropanation of polyalkene substrates, including terpene derivatives and conjugated 1,3-dienes. Mechanistic studies implicate a carbenoid species containing both Co and Zn as the catalytically relevant methylene transfer agent.

## Abstract 2

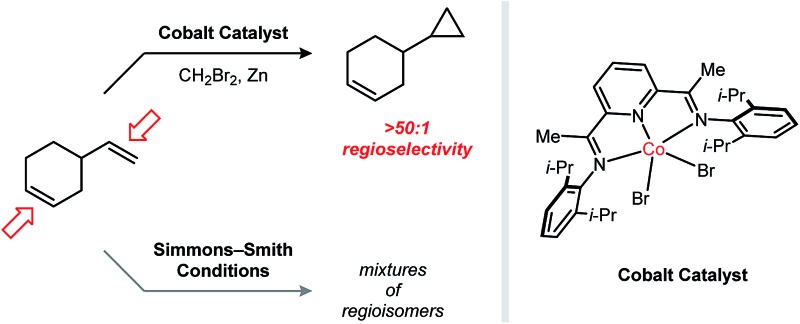
A cobalt catalyst imparts a high degree of regioselectivity in the reductive cyclopropanation of polyalkenes.

## Introduction

**Fig. 1:**
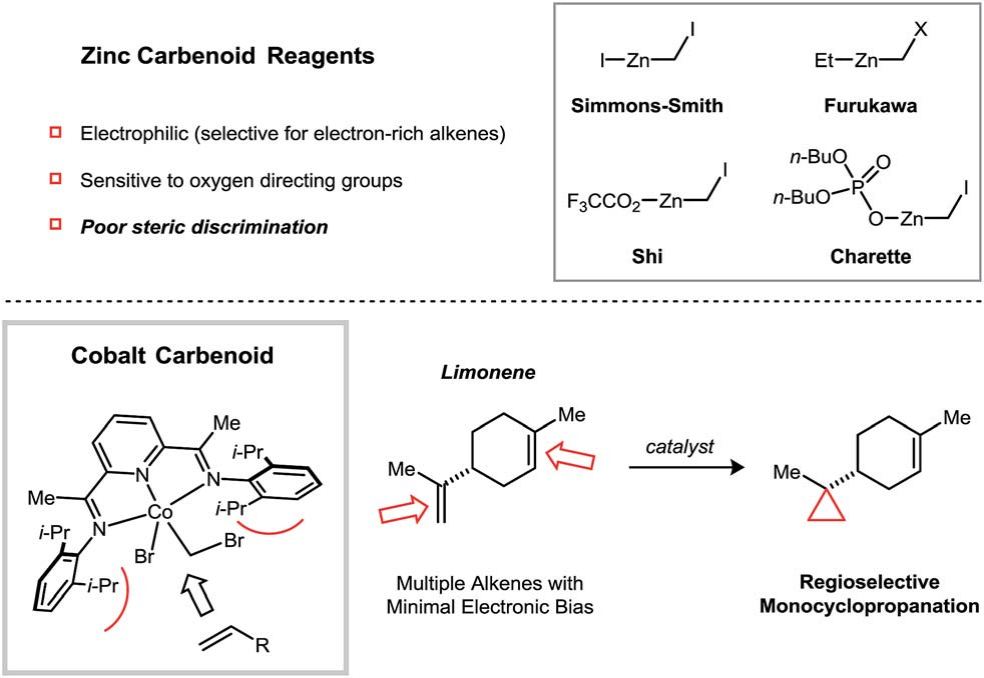
Factors governing alkene selectivity in Zn carbenoid-mediated cyclopropanation reactions. Cobalt-catalyzed reductive cyclopropanation exhibiting high regioselectivities in polyalkene substrates based on alkene substitution patterns.

Cyclopropanes are common structural elements in synthetic and natural biologically active compounds.^1^ The Simmons–Smith cyclopropanation reaction was first reported over half a century ago but remains today one of the most useful methods for converting an alkene into a cyclopropane.^2^ As compared to diazomethane, which is shock sensitive and must be prepared from complex precursors, CH_2_I_2_ is both stable and readily available, making it an attractive methylene source. Additionally, the stereospecificity of the Simmons–Smith reaction allows diastereomeric relationships in cyclopropanes to be established with a high degree of predictability. Several advances have addressed many of the limitations of the initial Simmons–Smith protocol. For example, Et_2_Zn can be used in the place of Zn to more reliably and quantitatively generate the active carbenoid reagent.^3^ Acidic additives, such as CF_3_CO_2_H^4^ and substituted phenols,^5^ have been found to accelerate the cyclopropanation of challenging substrates. Finally, Zn carbenoids bearing dialkylphosphate anions^6^ or bipyridine ligands^7^ are sufficiently stable to be stored in solution at low temperatures (Fig. 1).

Despite the many notable contributions in Zn carbenoid chemistry, a persistent limitation of Simmons–Smith-type cyclopropanations is their poor selectivity when attempting to discriminate between multiple alkenes of similar electronic properties. For example, the terpene natural product limonene possesses a 1,1-disubstituted and a trisubstituted alkene. Friedrich reported that, under a variety of Zn carbenoid conditions, the two alkenes are cyclopropanated with similar rates, resulting in mixtures of monocyclopropanated (up to a 5 : 1 ratio of regioisomers) and dicyclopropanated products.^8^ This issue is exacerbated by the challenge associated with separating the two monocyclopropane regioisomers, which only differ in the position of a non-polar CH_2_ group. In general, synthetically useful regioselectivities in Simmons–Smith reactions are only observed for substrates containing directing groups.^9^

In principle, catalysis may provide an avenue to address selectivity challenges in Simmons–Smith-type cyclopropanations; however, unlike diazoalkane transfer reactions, which are catalyzed by a broad range of transition metal complexes,9b,^10^ there has been comparatively little progress toward the development of robust catalytic strategies for reductive cyclopropanations.^11^ Lewis acids in substoichiometric loadings have been observed to accelerate the Simmons–Smith reaction, but in many cases, this rate effect is restricted to allylic alcohol substrates.^12^,^13^

Recently, our group described an alternative approach to catalyzing reductive cyclopropanation reactions using a transition metal complex that is capable of activating the dihaloalkane reagent by C–X oxidative addition. A dinickel catalyst was shown to promote methylene^14^ and vinylidene^15^ transfer using CH_2_Cl_2_ and 1,1-dichloroalkenes in combination with Zn as a stoichiometric reductant. Here, we describe a mononuclear [PDI]Co (PDI = pyridine-diimine) catalyst^16^ that imparts a high degree of steric selectivity in the cyclopropanation of polyalkene substrates. Mechanistic studies suggest that the key intermediate responsible for methylene transfer is a heterobimetallic conjugate of Co and Zn.

## Results and discussion

4-Vinyl-1-cyclohexene contains a terminal and an internal alkene of minimal electronic differentiation and thus provided a suitable model substrate to initiate our studies (Table 1).^8^,^17^ Under standard CH_2_I_2_/Et_2_Zn conditions (entry 1), there is a modest preference for cyclopropanation of the more electron-rich disubstituted alkene (rr = 1 : 6.7) with increasing amounts of competing dicyclopropanation being observed at higher conversions (entries 2 and 3). Other modifications to the conditions, including the use of a Brønsted acid^4^ (entry 4) or a Lewis acid additive12b,^18^ (entry 5), did not yield any improvements in selectivity. Likewise, an Al carbenoid generated using CH_2_I_2_ and AlEt_3_ afforded a similar preference for cyclopropanation of the endocyclic alkene (entry 6).^19^

**Table 1:** Regioselectivity studies using Zn and Al carbenoid reagents^*a*^

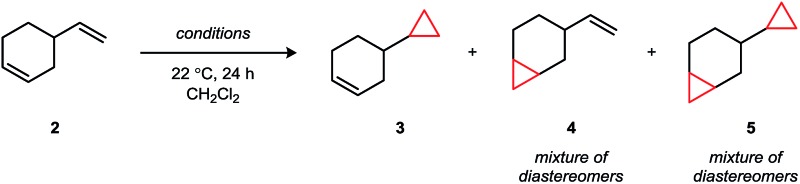				
Entry	Reaction conditions	Yield (3 + 4)	rr (3 : 4)	Yield 5
1	CH_2_I_2_ (1.0 equiv.), Et_2_Zn (0.5 equiv.)	28%	1 : 6.7	3%
2	CH_2_I_2_ (1.0 equiv.), Et_2_Zn (1.0 equiv.)	33%	1 : 4.6	5%
3	CH_2_I_2_ (2.0 equiv.), Et_2_Zn (2.0 equiv.)	53%	1 : 6.5	16%
4	CH_2_I_2_ (1.0 equiv.), Et_2_Zn (1.0 equiv.), 3,5-difluorobenzoic acid (2.0 equiv.)	28%	1 : 3.5	19%
5	CH_2_I_2_ (2.0 equiv.), Et_2_Zn (2.0 equiv.), TiCl_4_ (0.2 equiv.)	13%	1 : 4.6	1%
6	CH_2_I_2_ (1.2 equiv.), AlEt_3_ (1.2 equiv.)	38%	1 : 3.1	9%

^*a*^Reaction conditions: 4-vinylcyclohexene (0.14 mmol), CH_2_Cl_2_ (1.0 mL), 24 h, 22 °C. Yields and ratios of regioisomers were determined by GC analysis against an internal standard.

In a survey of transition metal catalysts, the [^*i*–Pr^PDI]CoBr_2_ complex **1** was identified as a highly regioselective catalyst for the cyclopropanation of 4-vinyl-1-cyclohexene, targeting the less hindered terminal alkene (Table 2). CH_2_Br_2_ and Zn alone do not afford any background levels of cyclopropanation (entry 1); however, the addition of 6 mol% [^*i*–Pr^PDI]CoBr_2_ (**1**) provided monocyclopropane **3** (81% yield) with a >50 : 1 rr and <1% of the dicyclopropane product (entry 5). The steric profile of the catalyst appears to be critically important for yield. For example, the mesityl- (entry 6) and phenyl-substituted variants (entry 7) of the ligand provided only 58% and 4% yield respectively under the same reaction conditions. Related N-donor ligands similarly afforded low levels of conversion (entries 8–12) as did the use of other first-row transition metals, including Fe (entry 14) and Ni (entry 15), in the place of Co.

**Table 2:** Catalyst structure–activity relationship studies^*a*^

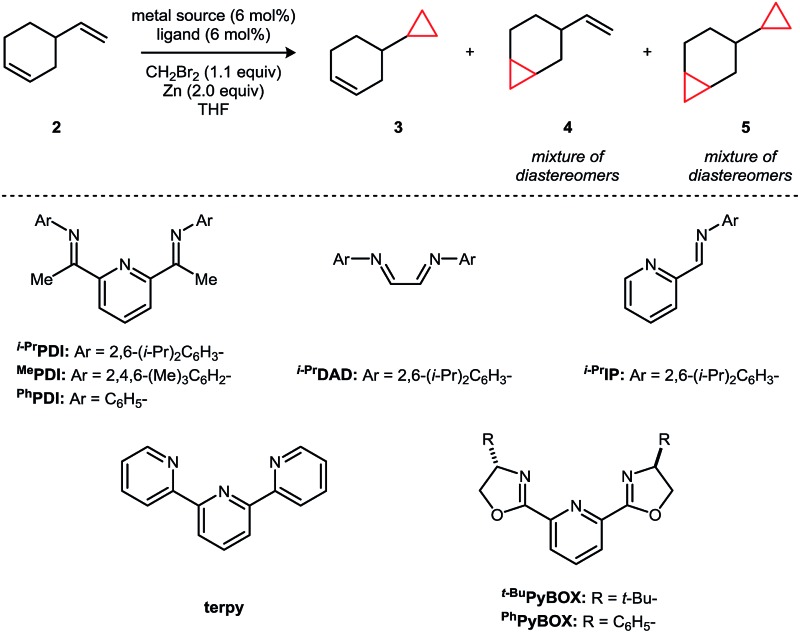					
Entry	Metal source	Ligand	Yield (3 + 4)	rr (3 : 4)	Yield 5
1	—	—	<1%	—	<1%
2	CoBr_2_	—	<1%	—	<1%
3	Co(DME)Br_2_	—	<1%	—	<1%
4	—	^*i*–Pr^PDI	<1%	—	<1%
5	CoBr_2_	^*i*–Pr^PDI	81%	>50 : 1	<1%
6	CoBr_2_	^Me^PDI	58%	>50 : 1	<1%
7	CoBr_2_	^Ph^PDI	4%	—	<1%
8	CoBr_2_	^*i*–Pr^DAD	<1%	—	<1%
9	CoBr_2_	^*i*–Pr^IP	2%	—	<1%
10	CoBr_2_	Terpy	4%	—	<1%
11	CoBr_2_	^*t*–Bu^PyBOX	<1%	—	<1%
12	CoBr_2_	^Ph^PyBOX	<1%	—	<1%
13	CoBr_2_	PPh_3_ (12 mol%)	<1%	—	0%
14	FeBr_2_	^*i*–Pr^PDI	3%	—	<1%
15	NiBr_2_	^*i*–Pr^PDI	<1%	—	<1%

^*a*^Reaction conditions: 4-vinylcyclohexene (0.14 mmol), THF (1.0 mL), 24 h, 22 °C. Yields and ratios of regioisomers were determined by GC analysis against an internal standard.

**Fig. 2:**
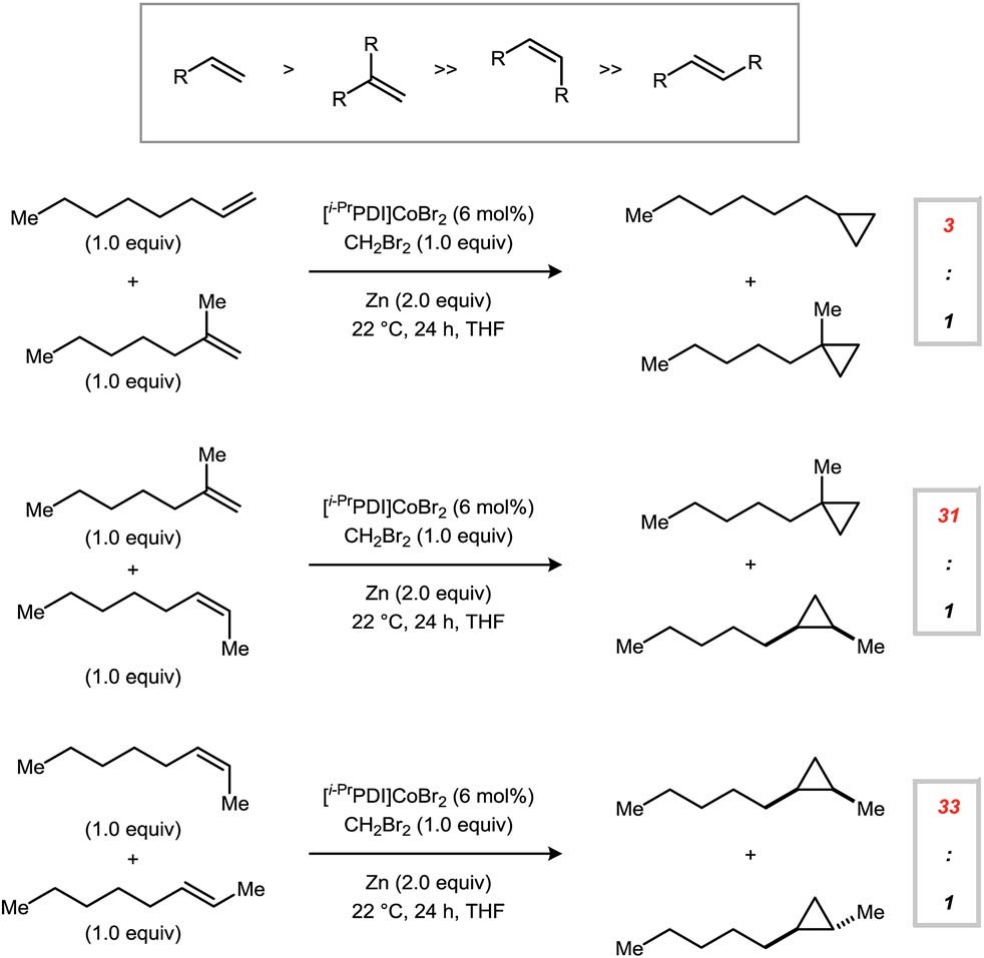
Intermolecular competition experiments probing selectivity based on alkene substitution patterns. Reactions were conducted using a 1 : 1 : 1 molar ratio of the two alkene starting materials and CH_2_Br_2_.

In order to define the selectivity properties of catalyst **1**, we next conducted competition experiments using alkenes bearing different patterns of substitution (Fig. 2). Reactions were carried out using an equimolar amount of each alkene and run to full conversion of the limiting CH_2_Br_2_ reagent (1.0 equiv.). Monosubstituted alkenes are the most reactive class of substrates using **1** but are not adequately differentiated from 1,1-disubstituted alkenes (3 : 1). By contrast, terminal alkenes are significantly more reactive than internal alkenes, providing synthetically useful selectivities (≥31 : 1). Furthermore, a model *Z*-alkene was cyclopropanated in preference to its *E*-alkene congener in a 33 : 1 ratio. Using catalyst **1**, trisubstituted alkenes are poorly reactive, and no conversion is observed for tetrasubstituted alkenes.

**Fig. 3:**
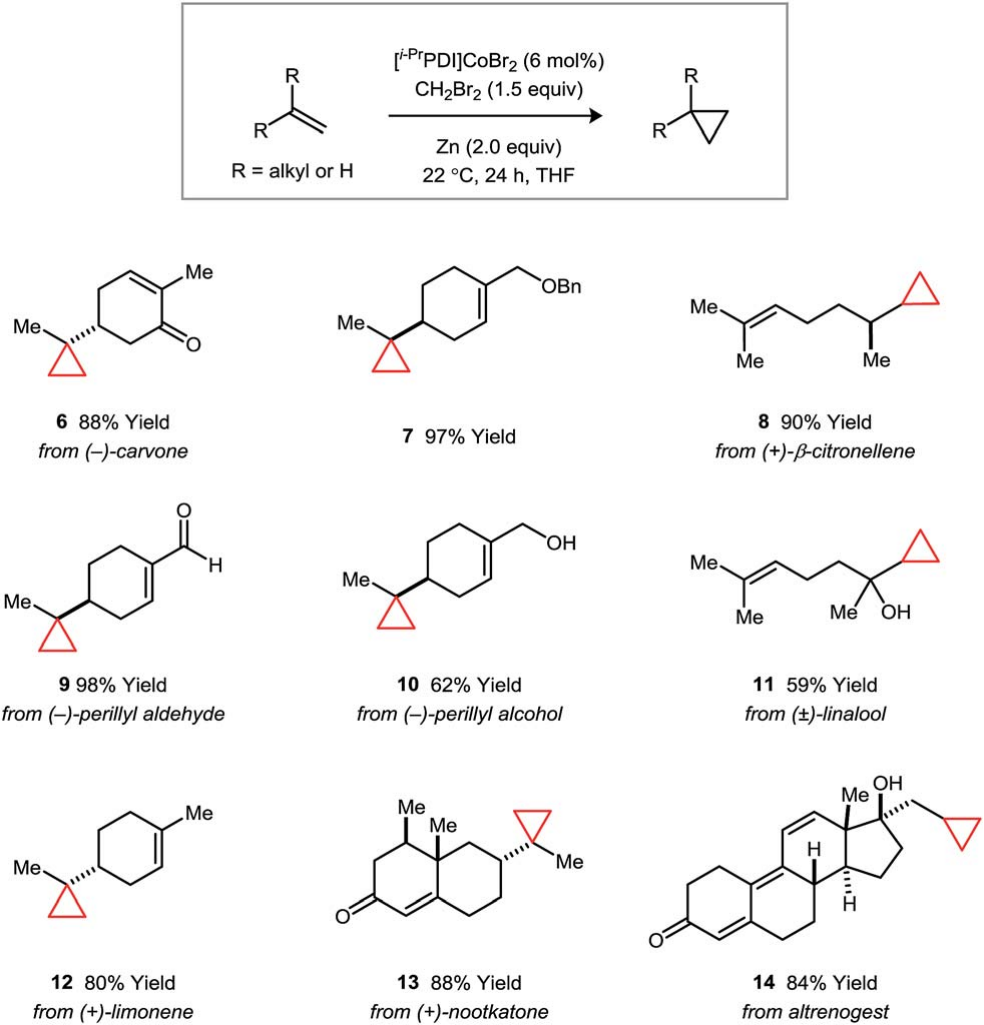
Catalytic regioselective monocyclopropanations of terpene natural products and derivatives. Isolated yields following purification are averaged over two runs.

The synthetic applications of the catalytic regioselective cyclopropanation were examined using the terpene natural products and derivatives shown in Fig. 3. In all cases, the selectivity properties follow the trends established in the competition experiments. Substrates containing ether or free alcohol functionalities (*e.g.*, **7**, **10**, and **11**) exhibit a strong directing group effect under classical Simmons–Smith conditions; however, catalyst **1** overrides this preference and targets the less hindered alkene. Additionally, the presence of electron-deficient α,β-unsaturated carbonyl systems (*e.g.*, **9**, **13**, and **14**) do not perturb the expected steric selectivity.

**Fig. 4:**
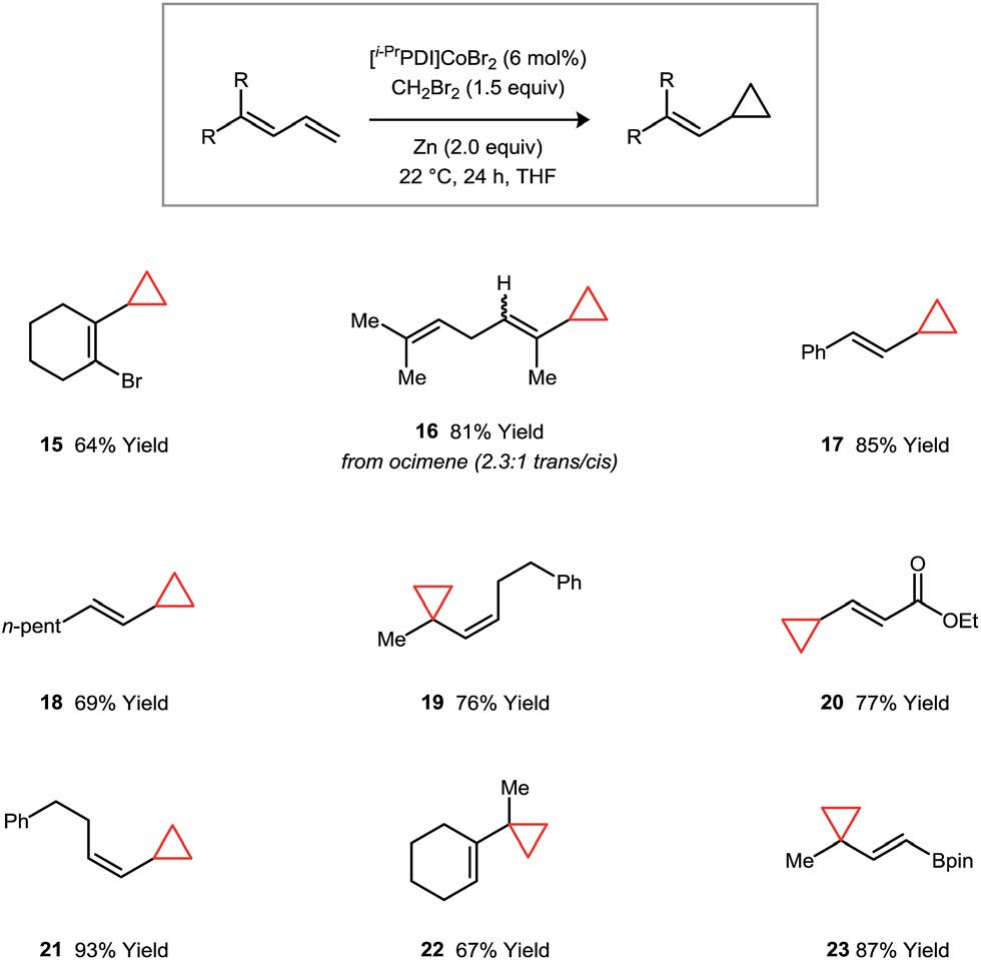
Catalytic regioselective monocyclopropanations of 1,3-dienes. Isolated yields following purification are averaged over two runs.

Vinylcyclopropanes are a valuable class of synthetic intermediates that engage in catalytic strain-induced ring-opening reactions.^20^ The monocyclopropanation of a diene represents an attractive approach to their synthesis but would require a catalyst that is capable of imparting a high degree of regioselectivity and avoiding secondary additions to form dicyclopropane products.^21^ These challenges are addressed for a variety of diene classes using catalyst **1** (Fig. 4). Over the substrates that we have examined, the selectivities for cyclopropanation of the terminal over the internal double bond of the diene system are uniformly high. Additionally, the catalyst is tolerant of vinyl bromide (**15**) and vinyl boronate (**23**) functional groups, which are commonly used in cross-coupling reactions.

**Fig. 5:**
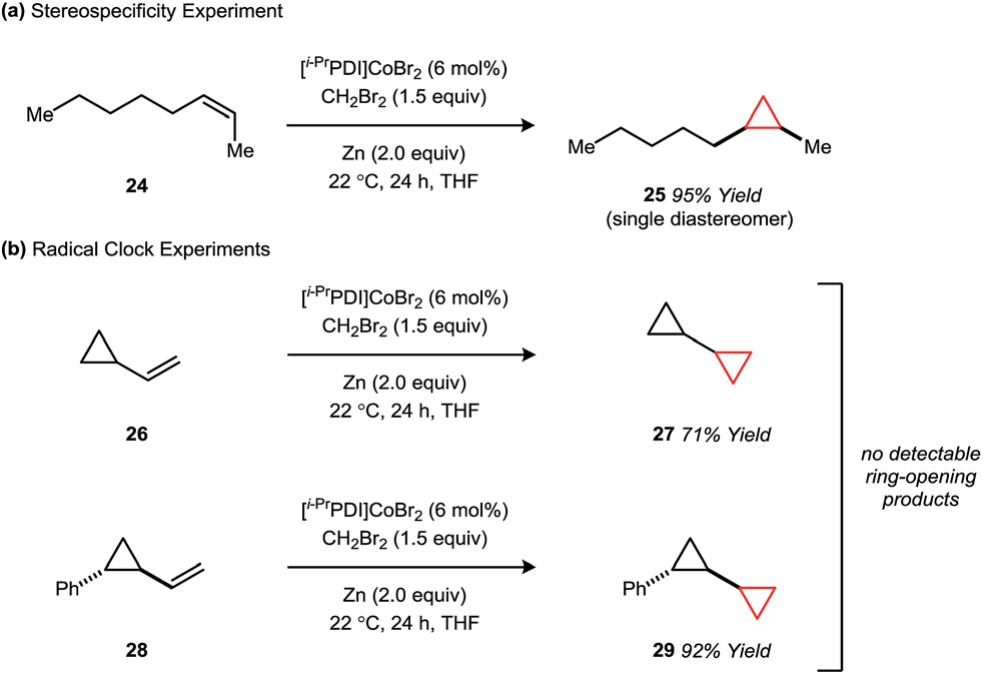
Mechanistic studies probing the concertedness of cyclopropane formation.

Like the non-catalytic Simmons–Smith reaction,2*c* the cyclopropanation using **1** is stereospecific within the limit of detection, implying a mechanism in which the two C–C σ-bonds are either formed in a concerted fashion or by a stepwise process that does not allow for single bond rotation. For example, cyclopropanation of the *Z*-alkene **24** affords the *cis*-disubstituted cyclopropane **25** in 95% yield as a single diastereomer (Fig. 5a). Furthermore, the vinylcyclopropane substrates **26** and **28**, commonly used as tests for cyclopropylcarbinyl radical intermediates, react without ring-opening to afford products **27** and **29** (Fig. 5b).

**Fig. 6:**
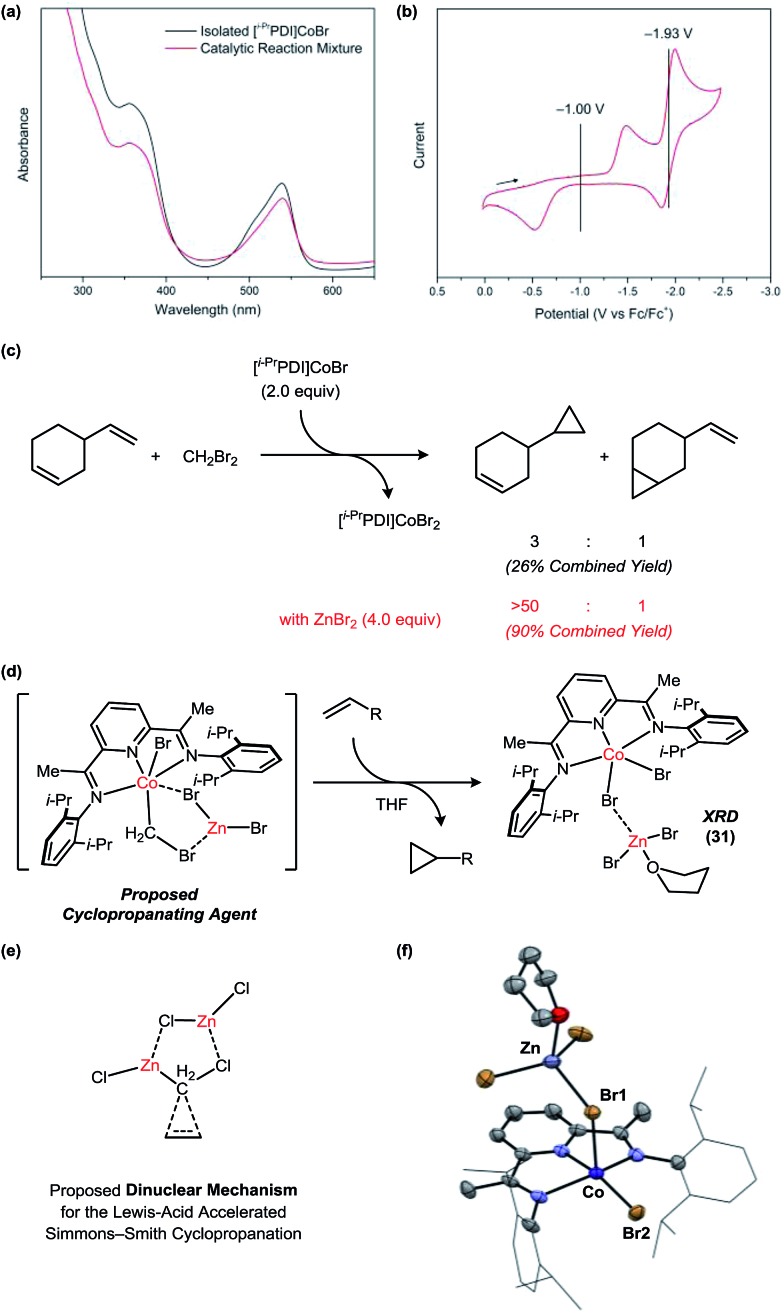
Mechanistic studies probing the nature of the active carbenoid intermediate. (a) Identification of the catalyst resting state. (b) Cyclic voltammetry data for the [^*i*–Pr^PDI]CoBr_2_ complex. (c) Stoichiometric cyclopropanation reactions using the [^*i*–Pr^PDI]CoBr_2_ complex in the absence and presence of ZnBr_2_. (d) A proposed Co/Zn carbenoid species. (e) Proposed dinuclear mechanism for the Lewis acid-accelerated Simmons–Smith reaction. (f) Solid-state structure for the [^*i*–Pr^PDI]CoBr_2_Zn(THF/Et_2_O)Br_2_ complex (**31**). The solvent molecule bound to Zn is disordered between Et_2_O and THF. Only the THF-bound structure is shown for clarity.

Under standard catalytic conditions, the reaction mixtures using **1** adopt a deep violet color, which persists until complete consumption of the alkene. The UV-vis spectrum of the catalytic mixture at partial conversion is consistent with a Co(i) resting state (Fig. 6a). The authentic [^*i*–Pr^PDI]CoBr complex (**30**) can be prepared by stirring the [^*i*–Pr^PDI]CoBr_2_ complex **1** over excess Zn metal.^22^ Cyclic voltammetry data (Fig. 6b) indicates an *E*_1/2_ for the Co(ii)/Co(i) redox couple of –1.00 V *vs.* Fc/Fc^+^. The large peak-to-peak separation (0.96 V in 0.3 M [*n*-Bu_4_N][PF_6_]/THF) is characteristic of a slow bromide dissociation step following electron transfer. The second Co(i)/Co(0) reduction event is significantly more cathodic at –1.93 V and is inaccessible using Zn.

In order to decouple the cyclopropanation steps of the mechanism from catalyst turnover, we conducted stoichiometric reactions with the isolated [^*i*–Pr^PDI]CoBr complex in the absence of Zn (Fig. 6c). The reaction of **30** with 4-vinylcyclohexene and CH_2_Br_2_ generates the [^*i*–Pr^PDI]CoBr_2_ complex **1** within 24 h at room temperature but forms cyclopropanated products in a relatively low combined yield of 26%, which is not commensurate with the efficiency of the catalytic process. Furthermore, the regioselectivity is only 3 : 1, whereas the catalytic cyclopropanation achieves a >50 : 1 selectivity for this substrate. When the same stoichiometric reaction is conducted in the presence of ZnBr_2_, the yield and selectivity of the catalytic process is fully restored.

The Co-containing product (**31**) of the stoichiometric reaction in the presence of ZnBr_2_ is green, which is notably distinct from the tan color of the [^*i*–Pr^PDI]CoBr_2_ complex **1**. This green species is NMR silent but may be crystallized from saturated solutions in Et_2_O to afford **31** (Fig. 6f). The solid-state structure reveals the expected [^*i*–Pr^PDI]CoBr_2_ fragment in a distorted square pyramidal geometry (*τ*_5_ = 0.36) with a Zn(THF/Et_2_O)Br_2_ Lewis acid coordinated to one of the Br ligands. This interaction induces an asymmetry in the structure, causing the Co–Br1 distance (2.557(1) Å) to be elongated relative to the Co–Br2 distance (2.358(2) Å).

Collectively, these studies suggest that both Co and Zn are present in the reactive carbenoid intermediate, and that ZnBr_2_ may interact with the [^*i*–Pr^PDI]Co complex through Lewis acid–base interactions. There is a notable similarity between the observed Co/Zn effect and previous studies of Lewis acid acceleration in the Simmons–Smith cyclopropanation. For example, Zn carbenoid reactions are known to be accelerated by the presence of ZnX_2_,12*c* which is generated as a byproduct of the reaction. DFT calculations conducted by Nakamura have suggested that the origin of this rate acceleration may be due to the accessibility of a five-membered ring transition state, which requires the presence of an additional Zn equivalent to function as a halide shuttle.^23^

## Conclusions

In summary, transition metal catalysis provides a pathway to accessing unique selectivity in reductive carbenoid transfer reactions. A [^*i*–Pr^PDI]CoBr_2_ complex functions as a robust catalyst for Simmons–Smith type cyclopropanation using a CH_2_Br_2_/Zn reagent mixture. This system exhibits the highest regioselectivities that have been observed in reductive cyclopropanations based solely on the steric properties of the alkene substrate. Accordingly, a range of terpenes and conjugated dienes may be converted to a single monocyclopropanated product. Ongoing studies are directed at exploring the applications of transition metal catalysts to other classes of carbenoid transfer reactions.

## Conflicts of interest

There are no conflicts to declare.

## Supplementary Material

Supplementary information

Crystal structure data
