# Supplementary material for: Regioselective Simmons–Smith-type cyclopropanations of polyalkenes enabled by transition metal catalysis
Source: Chem Sci. 2018 Jan 2;9(6):1604–9. doi: 10.1039/c7sc04861k (PMC5890799; doi:10.1039/c7sc04861k)
Supplement: Supplementary file 1 [file SC-009-C7SC04861K-s001.pdf]

Electronic Supplementary Material (ESI) for Chemical Science.
This journal is © The Royal Society of Chemistry 2018

Regioselective Simmons–Smith-type Cyclopropanations of Polyalkenes Enabled by Transition-Metal Catalysis

Jacob Werth and Christopher Uyeda

Department of Chemistry, Purdue University, West Lafayette, IN 47907, United States

Correspondence: cuyeda@purdue.edu

Supporting Information

1. General Information S2

2. Procedures for Zn and Al Carbenoid Cyclopropanations S3

3. Optimization Studies for Transition Metal-Catalyzed Cyclopropanations S10

4. Procedures for Competition Experiments S12

5. Procedures for Regioselective Monocyclopropanation Reactions S16

6. Procedures for Regioselective Monocyclopropanations of 1,3-Dienes S21

7. CV Data S25

8. Synthesis and Characterization of Co-Zn Complex S26

9. Mechanistic Experiments S29

10. NMR Spectra S31

11. IR Spectra S53

1. General Information

General considerations. All manipulations were carried out using standard Schlenk or glovebox techniques under an atmosphere of N_2. THF was dried and degassed by passing through a column of activated alumina and sparging with Ar gas. $CDCl_3$ was purchased from Cambridge Isotope Laboratories, Inc., degassed, and stored over activated 3 Å molecular sieves prior to use. All other reagents and starting materials were purchased from commercial vendors and used without further purification unless otherwise noted. PDI ligands and the [$^{i\text{-Pr}}$PDI]$CoBr_2$ complex **1** were synthesized according to reported methods.[1,2] Liquid reagents were degassed and stored over activated 3 Å molecular sieves prior to use. Zn powder (325 mesh, 99.9%) and $CoBr_2$ were purchased from Strem. $CoBr_2$ was dried in the oven and stored in the glovebox.

Physical methods. ^{1}H and ^{13}C{^{1}H} NMR spectra were collected at room temperature on a Varian INOVA 300 MHz spectrometer or a Bruker Avance 500 MHz spectrometer. ^{1}H and ^{13}C{^{1}H} NMR spectra are reported in parts per million relative to tetramethylsilane, using the residual solvent resonances as an internal standard. High-resolution mass data were obtained using an Agilent 6320 Trap LC/MS, Agilent 5975C GC/MS, or Thermo Electron Corporation MAT 95XP-Trap. ATR-IR data were collected on a Thermo Scientific Nicolet Nexus spectrometer. Elemental analysis was performed by Midwest Microlab (Indianapolis, IN).

X-ray crystallography. Single-crystal X-ray diffraction studies were carried out at the Purdue University X-ray crystallography facility.

Procedure for XRD data collected using the Bruker Quest instrument (Cu Source). Single crystals of were coated with mineral oil and quickly transferred to the goniometer head of a Bruker Quest diffractometer with kappa geometry, an I-μ-S microsource X-ray tube, laterally graded multilayer (Goebel) mirror single crystal for monochromatization, a Photon2 CMOS area detector and an Oxford Cryosystems low temperature device. Examination and data collection were performed with Cu Kα radiation (λ = 1.54178 Å) at 150 K. Data were collected, reflections were indexed and processed, and the files scaled and corrected for absorption using APEX3.[3]

Structure Solution and Refinement. The space groups were assigned and the structures were solved by direct methods using XPREP within the SHELXTL suite of programs[4] and refined by full matrix least squares against F^2 with all reflections using Shelxl2016[5] using the graphical interface Shelxle.[6] If not specified otherwise H atoms attached to carbon atoms were positioned geometrically and constrained to ride on their parent atoms, with carbon hydrogen bond distances of 0.95 Å for aromatic C-H, 1.00, 0.99 and 0.98 Å for aliphatic C-H, CH_2 and CH_3 moieties, respectively. Methyl H atoms were allowed to rotate but not to tip to best fit the experimental electron density. U_{iso}(H) values were set to a multiple of U_{eq}(C) with 1.5 for CH_3, and 1.2 for C-H units, respectively. Additional data collection and refinement details, including description of disorder can be found in Section 9 of the Supporting Information.

2. Procedures for Zn and Al Carbenoid Cyclopropanations

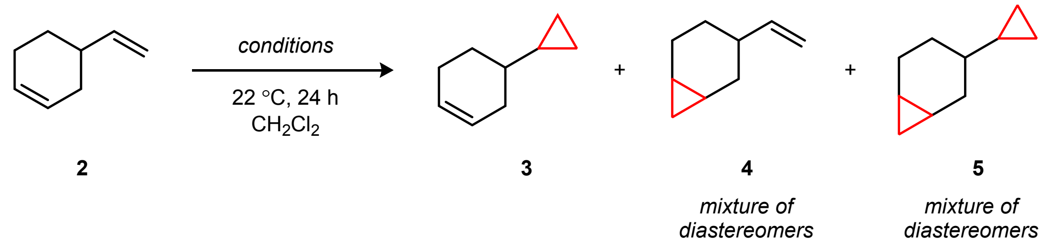

entry	reaction conditions	yield (**3** + **4**)	rr (**3**:**4**)	yield **5**
1	CH_2I_2 (1.0 equiv), Et_2Zn (0.5 equiv)	28%	1:6.7	3%
2	CH_2I_2 (1.0 equiv), Et_2Zn (1.0 equiv)	33%	1:4.6	5%
3	CH_2I_2 (2.0 equiv), Et_2Zn (2.0 equiv)	53%	1:6.5	16%
4	CH_2I_2 (2.0 equiv), Et_2Zn (2.0 equiv) 3,5-difluorobenzoic acid (2.0 equiv)	28%	1:3.5	19%
5	CH_2I_2 (2.0 equiv), Et_2Zn (2.0 equiv), $TiCl_4$ (20 mol%)	13%	1:4.6	1%
6	CH_2I_2 (1.2 equiv), $AlEt_3$ (1.2 equiv)	38%	1:3.1	9%

Figure S1. Comparison studies of Zn and Al carbenoid cyclopropanations.

Entry 1. In an N_2-filled glovebox, a 5-mL vial was charged with olefin (0.14 mmol, 1.0 equiv), Et_2Zn (8.7 mg, 0.070 mmol, 0.50 equiv), CH_2Cl_2 (0.5 mL), and a magnetic stir bar. A solution of CH_2I_2 (38 mg, 0.14 mmol, 1.0 equiv) in CH_2Cl_2 (0.5 mL) was added dropwise, and the reaction mixture was stirred at room temperature for 24 h. After 24 h, CH_2Cl_2 was added to dilute the solution and an aliquot was used for GC analysis.

Entries 2 and 3. See procedure for Entry 1 with appropriate modifications to the equivalents of CH_2I_2 and Et_2Zn.

Entry 4. In an N_2-filled glovebox, a 5-mL vial was charged with olefin (0.14 mmol, 1.0 equiv), Et_2Zn (35 mg, 0.28 mmol, 2.0 equiv), CH_2Cl_2 (0.5 mL), and a magnetic stir bar. 3,5-Difluorobenzoic acid (43 mg, 0.28 mmol, 2.0 equiv) was added in portions to slow evolution of ethane gas. A solution of CH_2I_2 (75 mg, 0.28 mmol, 2.0 equiv) in CH_2Cl_2 (0.5 mL) was added dropwise, and the reaction mixture was stirred at room temperature for 24 h. After 24 h, CH_2Cl_2 was added to dilute the solution and an aliquot was used for GC analysis.

Entry 5. In an N_2-filled glovebox, a 5-mL vial was charged with olefin (0.14 mmol, 1.0 equiv), Et_2Zn (35 mg, 0.28 mmol, 2.0 equiv), CH_2Cl_2 (0.5 mL), and a magnetic stir bar. A solution of CH_2I_2 (75 mg, 0.28 mmol, 2.0 equiv) in CH_2Cl_2 (0.5 mL) was added dropwise. $TiCl_4$ (5.3 mg, 0.028 mmol, 0.2 equiv) was added dropwise, and the reaction mixture was stirred at room temperature for 24 h. After 24 h, CH_2Cl_2 was added to dilute the solution and an aliquot was used for GC analysis.

Entry 6. In an N_2-filled glovebox, a 5-mL vial was charged with olefin (0.14 mmol, 1.0 equiv), Et_3Al (20 mg, 0.17 mmol, 1.2 equiv), CH_2Cl_2 (0.5 mL), and a magnetic stir bar. A solution of CH_2I_2 (45 mg, 0.17 mmol, 1.2 equiv) in CH_2Cl_2 (0.5 mL) was added dropwise, and the reaction mixture

was stirred at room temperature for 24 h. After 24 h, CH_2Cl_2 was added to dilute the solution and an aliquot was used for GC analysis.

Entry 1

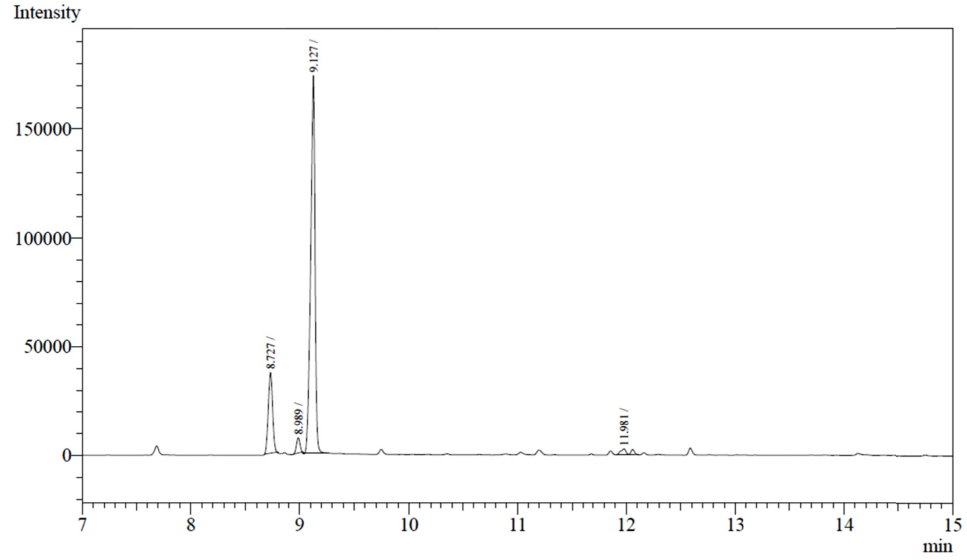

Peak#	Ret.Time	Area	Height
1	8.727	101196	36842
2	8.989	15143	6950
3	9.127	477195	171955
4	11.981	13560	2722
Total		607094	218469

Entry 2

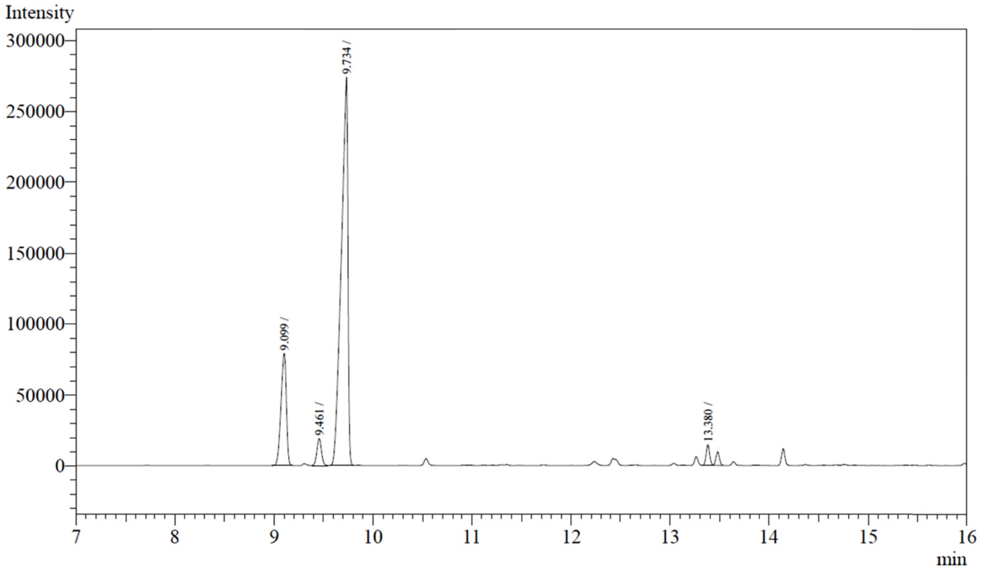

Peak#	Ret.Time	Area	Height
1	9.099	297373	78921
2	9.461	64586	19550
3	9.734	1255396	272338
4	13.380	60218	14604
Total		1677573	385413

Entry 3

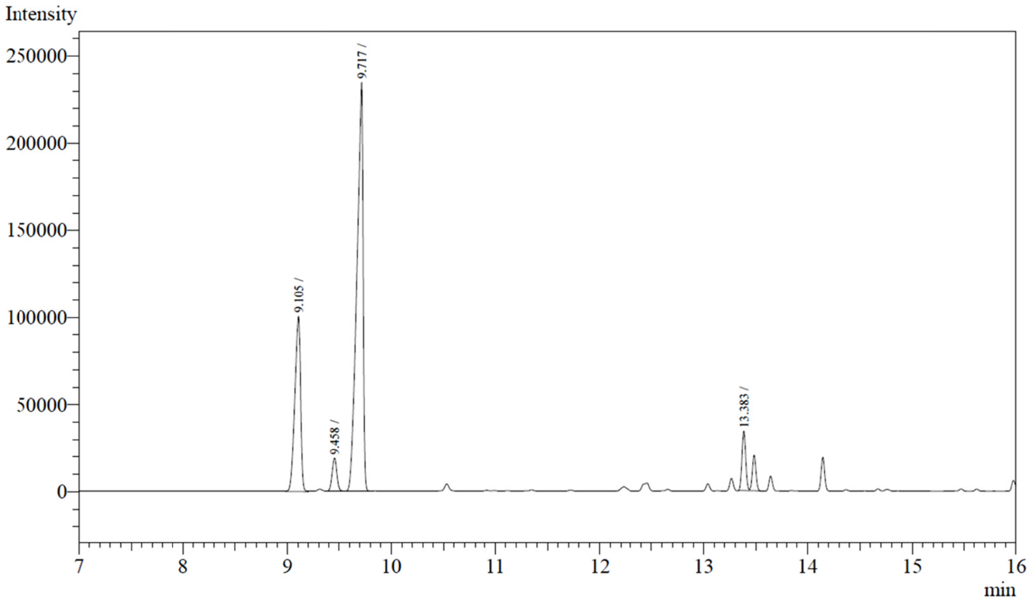

Peak#	Ret.Time	Area	Height
1	9.105	385361	100093
2	9.458	58944	18895
3	9.717	961668	232079
4	13.383	133669	34217
Total		1539642	385284

Entry 4

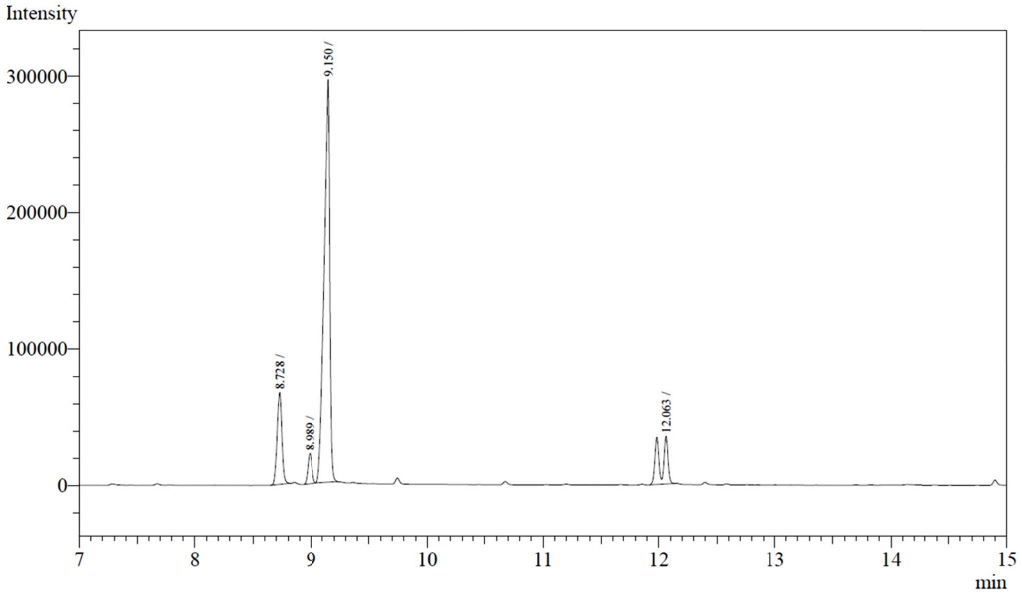

Peak#	Ret.Time	Area	Height
1	8.728	192745	66693
2	8.989	55303	22053
3	9.150	999362	293992
4	12.063	163995	34994
Total		1411405	417732

Entry 5

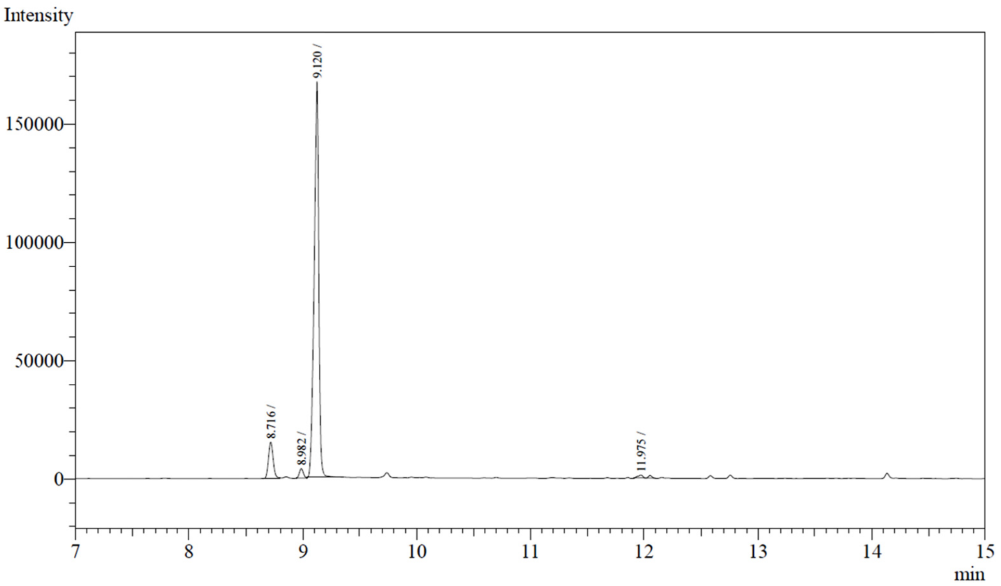

Peak#	Ret.Time	Area	Height
1	8.716	43219	15347
2	8.982	9459	3931
3	9.120	467288	165720
4	11.975	5659	1225
Total		525625	186223

Entry 6

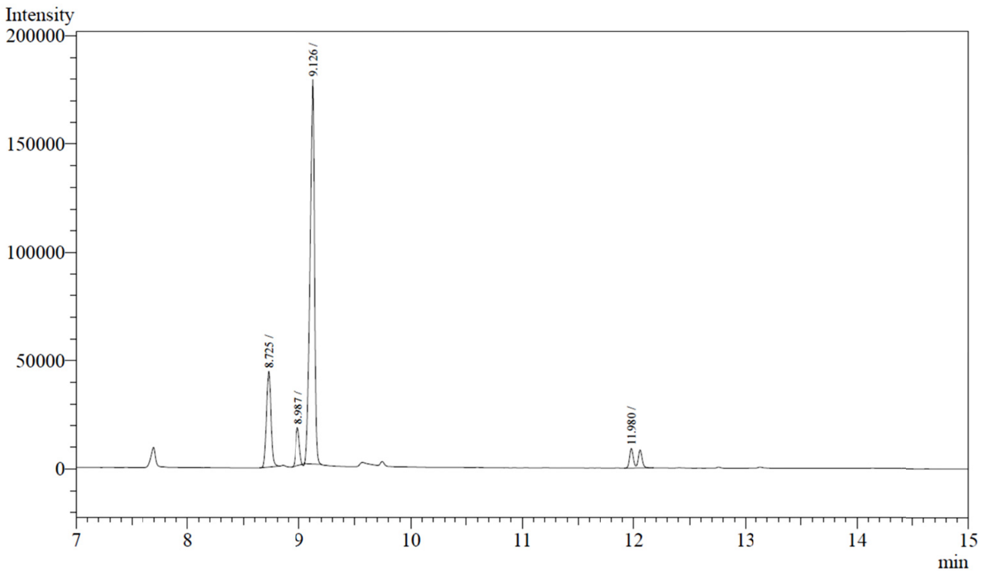

Peak#	Ret.Time	Area	Height
1	8.725	124858	44144
2	8.987	40612	17567
3	9.126	493344	176963
4	11.980	40457	8866
Total		699271	247540

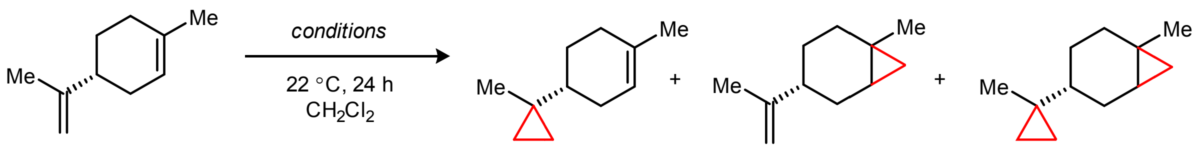

entry	reaction conditions[a]	yield (a + b)	rr (a:b)	yield c
1	CH_2I_2 (1.0 equiv), Et_2Zn (0.5 equiv)	35%	1:1.6	9%
2	CH_2I_2 (2.0 equiv), Et_2Zn (2.0 equiv) 3,5-difluorobenzoic acid (2.0 equiv)	39%	2.1:1	32%
3	CH_2I_2 (2.0 equiv), Et_2Zn (2.0 equiv), $TiCl_4$ (20 mol%)	35%	1:1.7	27%
4	CH_2I_2 (1.2 equiv), $AlEt_3$ (1.2 equiv)	45%	1.6:1	46%

[a]conducted using the same procedues as for 4-vinylcyclohexene)

Figure S2. GC data for Zn and Al carbenoid cyclopropanations.

Entry 1

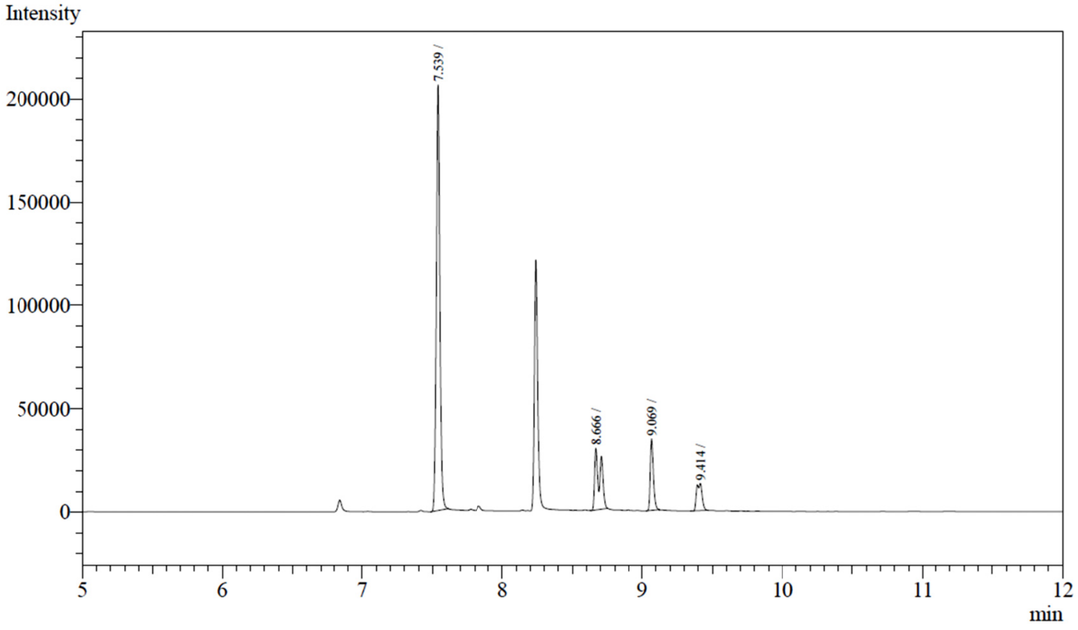

Peak#	Ret.Time	Area	Height
1	7.539	357444	204052
2	8.666	83761	29133
3	9.069	53387	33763
4	9.414	35706	12978
Total		530298	279926

Entry 2

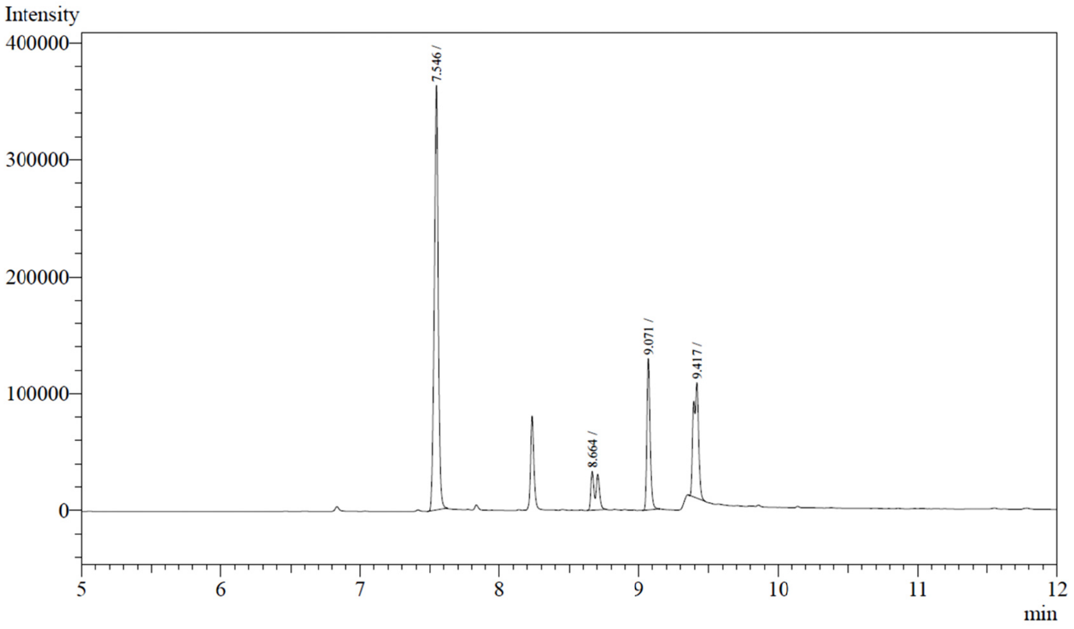

Peak#	Ret.Time	Area	Height
1	7.546	705695	355398
2	8.664	100337	33171
3	9.071	207278	127873
4	9.417	254091	97258
Total		1267401	613700

Entry 3

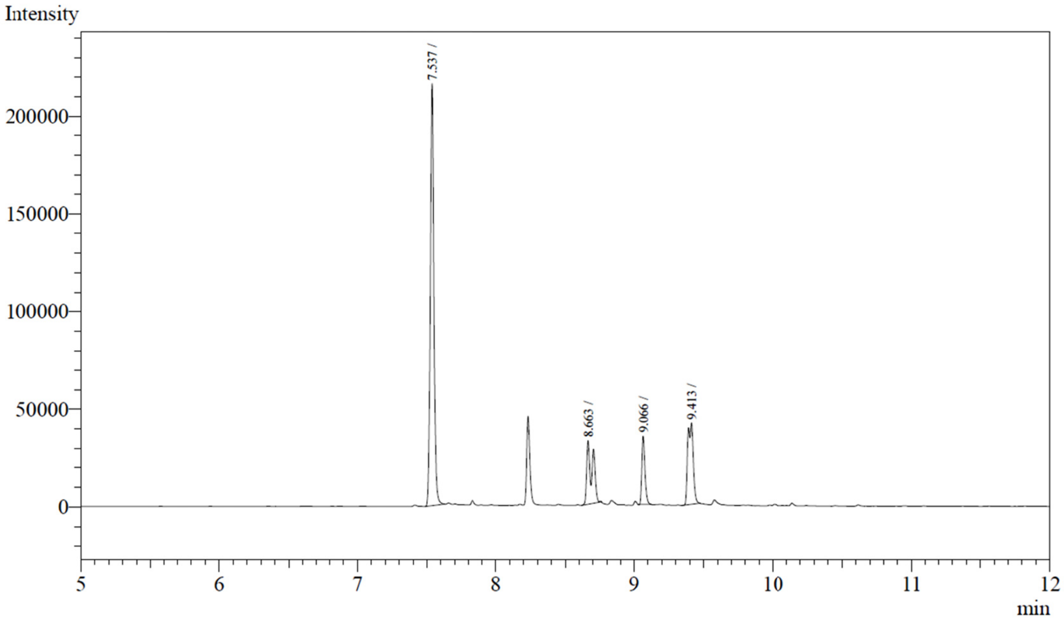

Peak#	Ret.Time	Area	Height
1	7.537	381855	212434
2	8.663	92874	31986
3	9.066	53781	34663
4	9.413	114968	41006
Total		643478	320089

Entry 4

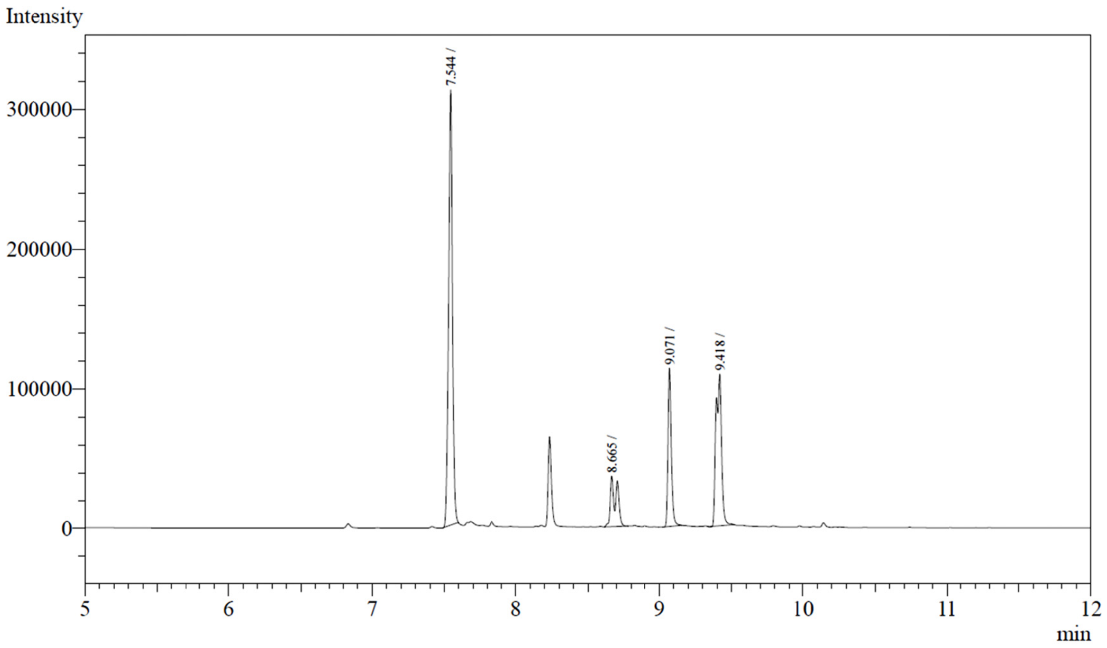

Peak#	Ret.Time	Area	Height
1	7.544	573759	307795
2	8.665	108866	36058
3	9.071	179599	112211
4	9.418	291031	105018
Total		1153255	561082

3. Optimization Studies for Transition Metal-Catalyzed Cyclopropanations

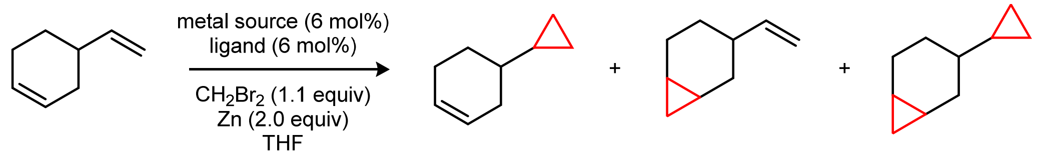

2 **3** **4** *mixture of diastereomers* **5** *mixture of diastereomers*

entry	metal source	ligand	yield (**3** +**4**)	rr (**3**:**4**)	yield **5**
1	–	–	<1%	–	<1%
2	$CoBr_2$	–	<1%	–	<1%
3	Co(DME)Br_2	–	<1%	–	<1%
4	–	$^{i\text{-Pr}}$PDI	<1%	–	<1%
5	$CoBr_2$	$^{i\text{-Pr}}$PDI	81%	>50:1	<1%
6	$CoBr_2$	^{Me}PDI	58%	>50:1	<1%
7	$CoBr_2$	^{Ph}PDI	4%	–	<1%
8	$CoBr_2$	$^{i\text{-Pr}}$DAD	<1%	–	<1%
9	$CoBr_2$	$^{i\text{-Pr}}$IP	2%	–	<1%
10	$CoBr_2$	terpyridine	4%	–	<1%
11	$CoBr_2$	(*S*)-$^{t\text{-Bu}}$PyBOX	<1%	–	<1%
12	$CoBr_2$	(*S*)-^{Ph}PyBOX	<1%	–	<1%
13	$CoBr_2$	$[PPh_3]_2$	<1%	–	0%
14	$FeBr_2$	$^{i\text{-Pr}}$PDI	3%	–	<1%
15	$NiBr_2$	$^{i\text{-Pr}}$PDI	<1%	–	<1%

Figure S3. Optimization studies probing metal and ligand sources.

General Procedure. In an N_2-filled glovebox, a 3-mL vial was charged with the metal source (0.0084 mmol, 0.060 equiv), ligand (0.0084 mmol, 0.060 equiv), THF (0.5 mL), and a magnetic stir bar. The catalyst mixture was allowed to stir at room temperature for 24 h. After this premixing period, 4-vinylcyclohexene (0.14 mmol, 1.0 equiv), CH_2Br_2 (27 mg, 0.15 mmol, 1.1 equiv), Zn powder (18 mg, 0.28 mmol, 2.0 equiv), mesitylene internal standard, and THF (0.5 mL) were added. The reaction mixture was stirred at room temperature for 24 h. After 24 h, CH_2Cl_2 was added to dilute the solution and an aliquot was used for GC analysis.

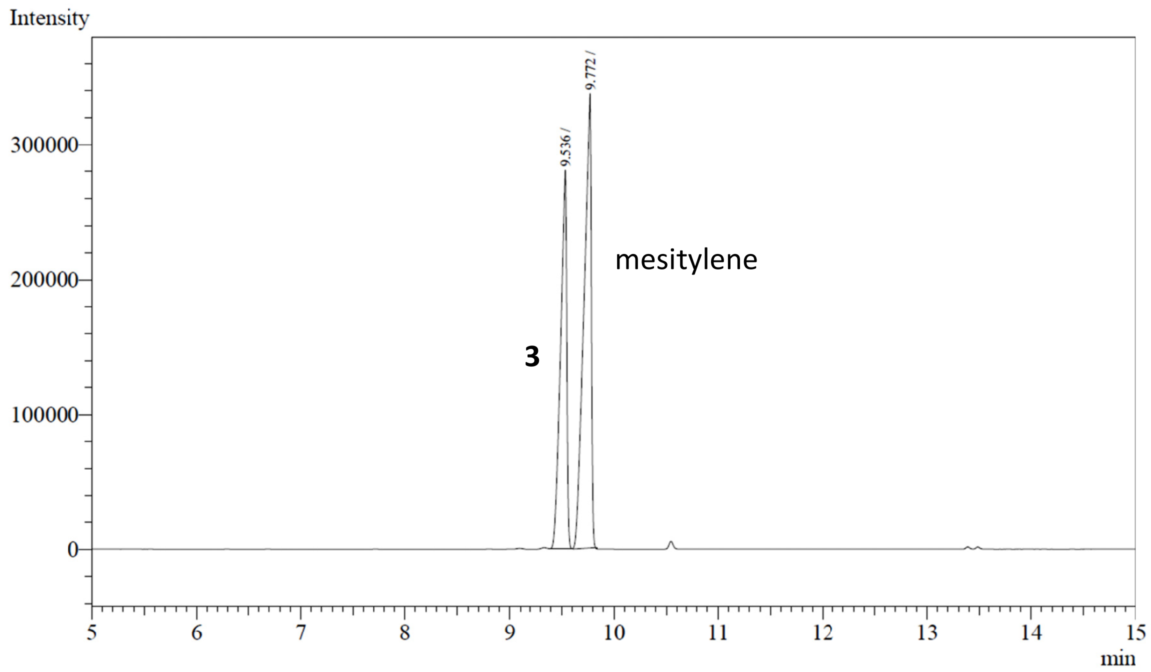

Peak#	Ret.Time	Area	Height
1	9.536	1150313	280109
2	9.772	1625979	334056
Total		2776292	614165

Figure S4. GC data for entry 5 in Figure S3.

4. Procedures for Competition Experiments

In an N_2-filled glovebox, a 3-mL vial was charged with [$^{i\text{-Pr}}$PDI]$CoBr_2$ (5.9 mg, 0.0084 mmol, 0.06 equiv), each alkene (0.14 mmol, 1.0 equiv), CH_2Br_2 (24 mg, 0.14 mmol, 1.0 equiv), Zn powder (18 mg, 0.28 mmol, 2.0 equiv), THF (1.0 mL), and a magnetic stir bar. The reaction was stirred for 24h. An aliquot was diluted with CH_2Cl_2 and used for GC analysis. Relative response factors of all products were assumed to be the same. Retention times were determined using authentic samples of each cyclopropane product.

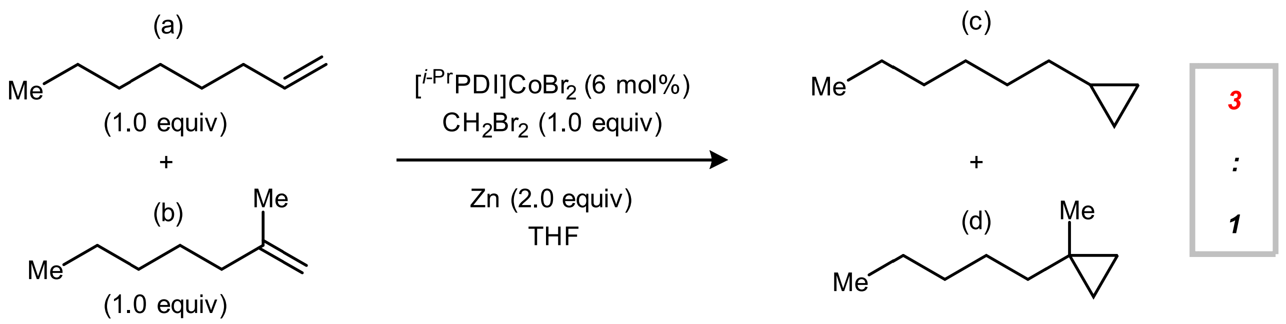

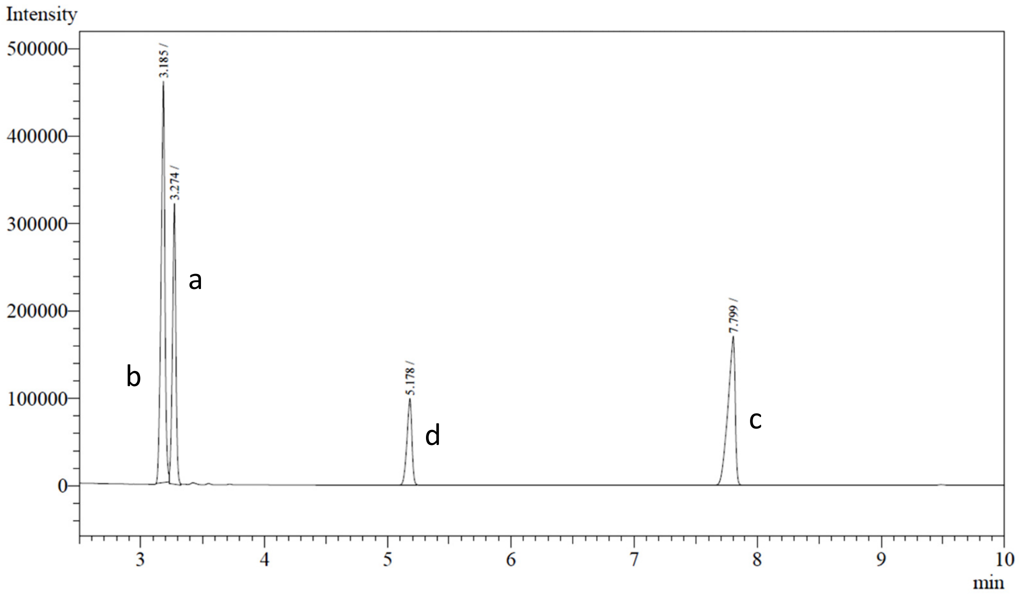

Peak#	Ret.Time	Area	Height
1	3.185	986453	455676
2	3.274	615707	318744
3	5.178	279016	99439
4	7.799	695532	170048
Total		2576708	1043907

Figure S5. GC data for the competition experiment between 1-octene and 2-methyl-1-hepetene.

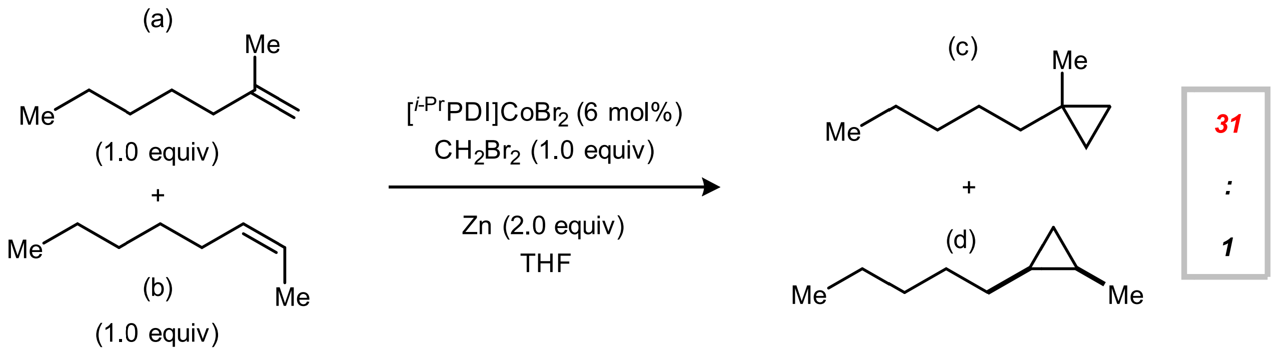

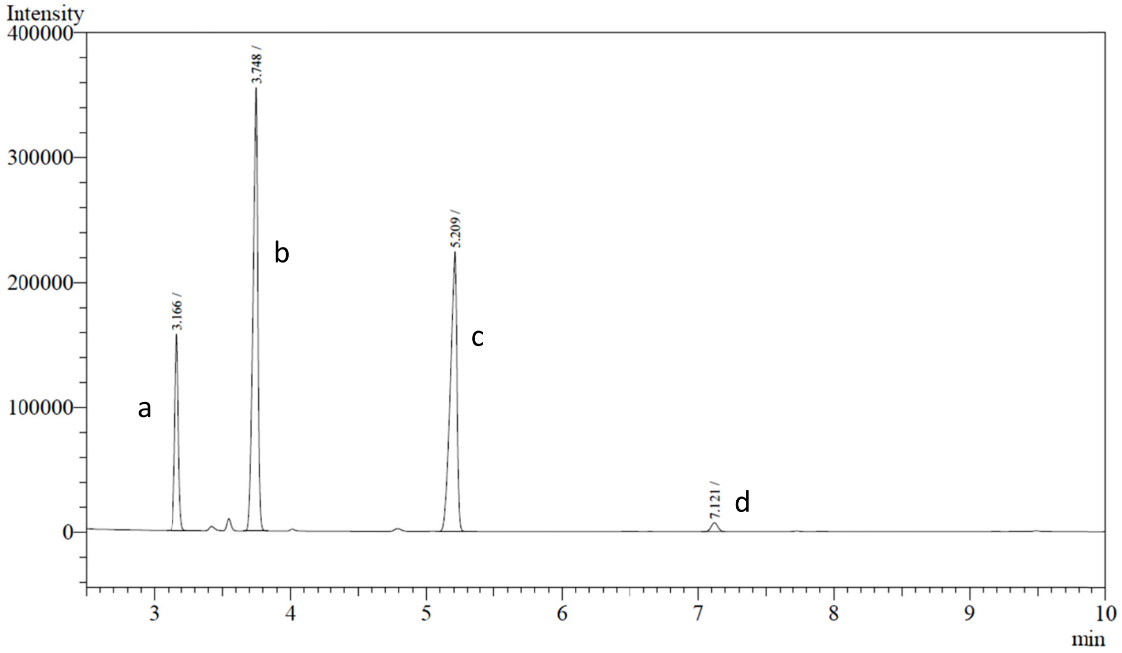

Peak#	Ret.Time	Area	Height
1	3.166	291888	155792
2	3.748	861070	350083
3	5.209	745194	222654
4	7.121	24008	7077
Total		1922160	735606

Figure S6. GC data for the competition experiment between 2-methyl-1-hepetene and (*Z*)-2-octene.

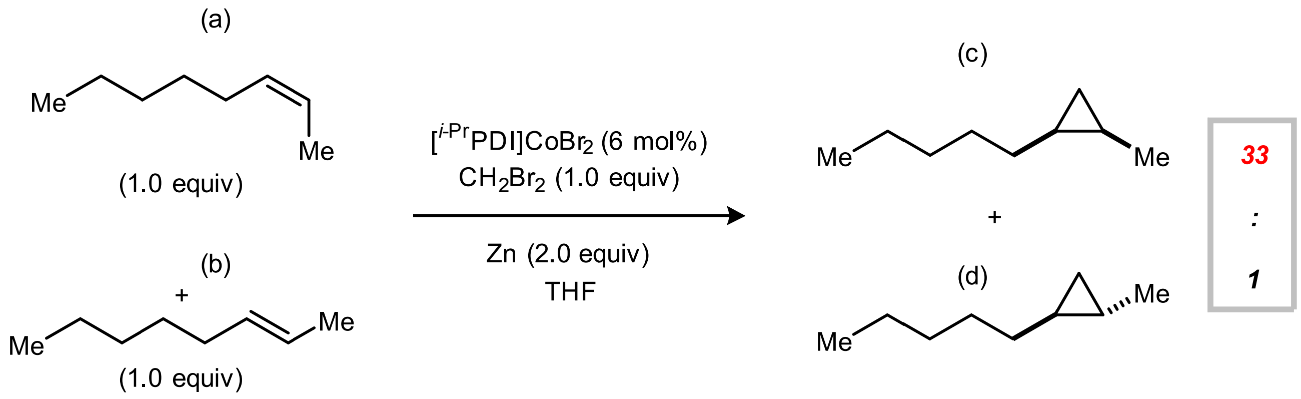

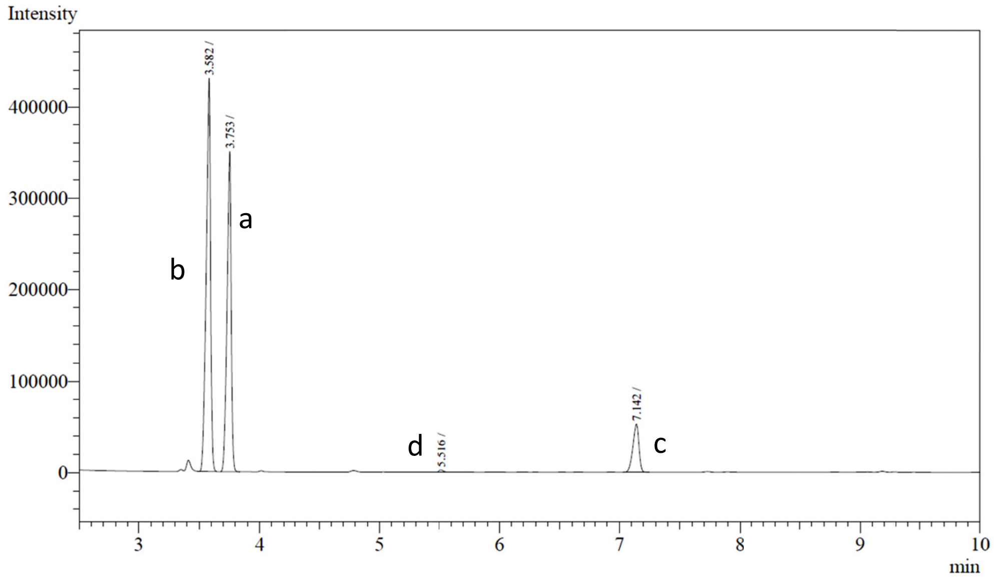

Peak#	Ret.Time	Area	Height
1	3.582	1056255	427254
2	3.753	821866	342851
3	5.516	5517	2178
4	7.142	183591	52909
Total		2067229	825192

Figure S7. GC data for the competition experiment between (*Z*)-2-octene and (*E*)-2-octene using 1.0 equiv of CH_2Br_2.

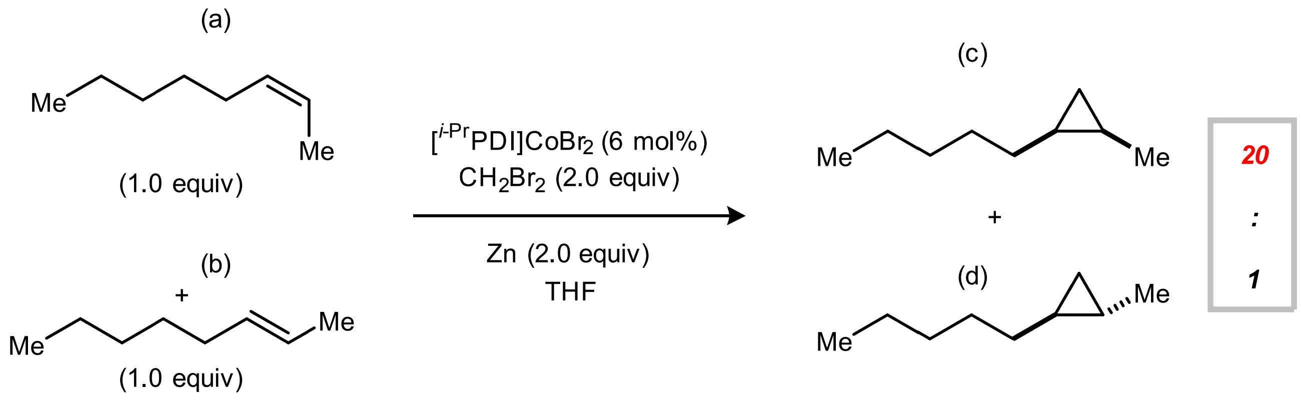

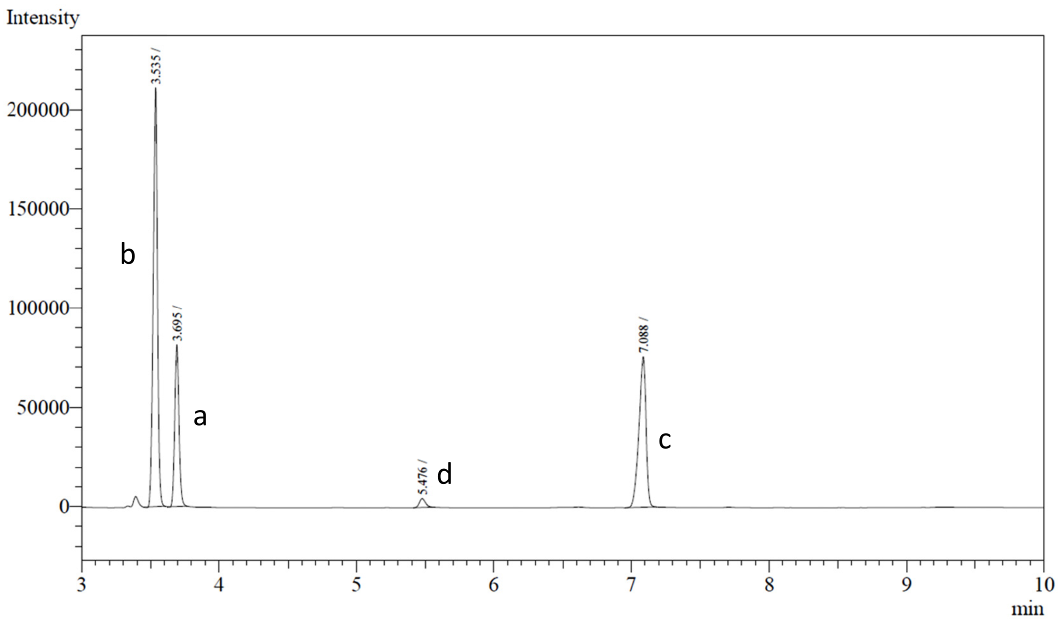

Peak#	Ret.Time	Area	Height
1	3.535	450067	209429
2	3.695	173222	80725
3	5.476	13583	4450
4	7.088	271118	75461
Total		907990	370065

Figure S8. GC data for the competition experiment between (*Z*)-2-octene and (*E*)-2-octene using 2.0 equiv of CH_2Br_2.

5. Procedures for Regioselective Monocyclopropanation Reactions

General Procedure. In an N_2-filled glovebox, a 3-mL vial was charged with [$^{i\text{-Pr}}$PDI]$CoBr_2$ (5.9 mg, 0.0084 mmol, 0.06 equiv), the substrate (0.14 mmol, 1.0 equiv), CH_2Br_2 (37 mg, 0.21 mmol, 1.5 equiv), Zn powder (18 mg, 0.28 mmol, 2.0 equiv), THF (1.0 mL), and a magnetic stir bar. The reaction was stirred at room temperature. After 24 h, the reaction mixture was concentrated under reduced pressure, and the crude residue was directly loaded onto a column for purification.

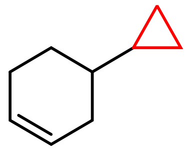

(4). The reaction was conducted using 4-vinylcyclohexene (18 µL) without modification from the general procedure to provide **4** as a colorless oil.

NMR Yield: Run 1: 88% yield. Run 2: 81% yield.

Isolated Yield: 11.7 mg (68% yield)

Purification: SiO_2 column; pentane

^{1}H NMR (300 MHz, $CDCl_3$) δ 5.65 (s, 2H), 2.18-1.98 (m, 3H), 1.90-1.80 (m, 2H), 1.45-1.31 (m, 1H), 0.84-0.72 (m, 1H), 0.63-0.53 (m, 1H), 0.42-0.37 (m, 2H), 0.10-0.06 (m, 2H).

^{13}C{^{1}H} NMR (126 MHz, $CDCl_3$) δ 127.0, 126.7, 39.0, 31.6, 28.6, 25.3, 16.9, 3.2, 3.0.

HRMS (EI) calc. for $C_9H_{14}^+$: m/z=122.1090, found: m/z=122.1091

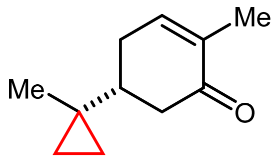

(6). The reaction was conducted using (–)-Carvone (19 µL) without modification from the general procedure to provide **6** as a yellow oil.

Run 1: 19.1 mg (83% yield). Run 2: 21.2 mg (92% yield). 1g scale: 922 mg (91% yield)

Purification: SiO_2 column; CH_2Cl_2

^{1}H NMR (300 MHz, $CDCl_3$) δ 6.76-6.73 (m, 1H), 2.47 (dd, J = 3.78, 16.05 Hz, 1H), 2.34-2.24 (m, 3H), 1.74 (q, J = 1.69 Hz, 3H), 1.41-1.28 (m, 1H), 0.96 (s, 3H), 0.27 (s, 4H).

^{13}C{^{1}H} NMR (126 MHz, $CDCl_3$) δ 200.6, 145.3, 135.3, 44.2, 42.2, 29.6, 19.1, 18.3, 15.7, 12.3, 12.3.

HRMS (ESI) calc. for $C_{11}H_{17}O$: m/z=165.1280, found: m/z=165.1274

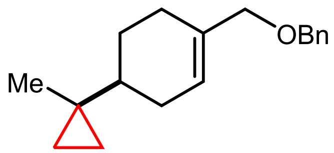

(7). The reaction was conducted using benzyloxylimonene[7] (34 mg) without modification from the general procedure to provide **7** as a colorless oil.

Run 1: 33.7 mg (94% yield). Run 2: 35.5 mg (99% yield).

Purification: SiO_2 column; CH_2Cl_2

^{1}H NMR (300 MHz, $CDCl_3$) δ 7.36-7.27 (m, 5H), 5.73 (s, 1H), 4.47 (s, 2H), 3.89 (s, 2H), 2.21-2.12 (m, 1H), 2.09-2.00 (m, 3H), 1.86-1.77 (m, 1H), 1.49-1.37 (m, 1H), 0.95 (s, 3H), 0.90-0.82 (m, 1H), 0.30-0.21 (m, 4H).

^{13}C{^{1}H} NMR (126 MHz, $CDCl_3$) δ 138.7, 134.7, 128.3, 127.7, 127.4, 125.3, 74.7, 71.6, 42.2, 28.3, 27.1, 26.3, 19.2, 18.7, 12.4, 12.3.

HRMS (ESI) calc. for $C_{18}H_{25}O$: m/z=257.1906, found: m/z=257.1903

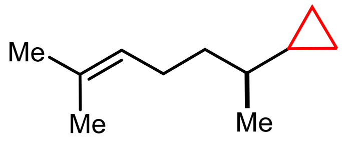

(8). The reaction was conducted using (+)-β-citronellene (25 μL) without modification from the general procedure to provide **8** as a colorless oil.

Run 1: 19.8 mg (93% yield). Run 2: 18.5 mg (87% yield).

Purification: SiO_2 column; pentane

^{1}H NMR (300 MHz, $CDCl_3$) δ 5.10 (t, *J* = 7.09 Hz, 1H), 2.03 (q, *J* = 7.65, 2H), 1.68 (s, 3H), 1.61 (s, 3H), 1.51-1.39 (m, 1H), 1.35-1.23 (m, 2H), 0.95, (d, *J* = 6.6 Hz, 3H), 0.73-0.61 (m, 1H), 0.52-0.30 (m, 2H), 0.12-(-0.07) (m, 2H).

^{13}C{^{1}H} NMR (126 MHz, $CDCl_3$) δ 131.0, 125.2, 38.2, 37.5, 25.8, 25.7, 19.8, 18.0, 17.6, 4.4, 2.9.

HRMS (EI) calc. for $C_{11}H_{20}^+$: m/z=152.1560, found: m/z=152.1563

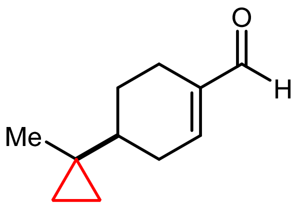

(9). The reaction was conducted using (−)-perillaldehyde (22 μL) without modification from the general procedure to provide **9** as a light yellow oil.

Run 1: 22.3 mg (97% yield). Run 2: 22.5 mg (98% yield).

Purification: SiO_2 column; CH_2Cl_2

^{1}H NMR (300 MHz, $CDCl_3$) δ 9.41 (s, 1H), 6.83-6.80 (m, 1H), 2.52-2.44 (m, 1H), 2.33-2.23 (m, 1H), 2.07-1.92 (m, 1H), 1.89-1.82 (m, 1H), 1.41-1.14 (m, 2H), 0.95 (s, 3H), 0.93-0.86 (m, 1H), 0.28-0.17 (m, 4H).

$^{13}C\{^{1}H\}$ NMR (126 MHz, $CDCl_3$) δ 194.1, 151.6, 141.5, 42.1, 29.8, 25.3, 22.2, 18.9, 18.4, 12.5.

HRMS (ESI) calc. for $C_{11}H_{17}O$: m/z=165.1280, found: m/z=165.1273

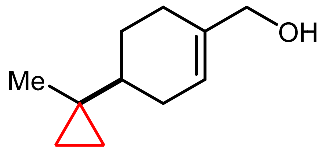

(10). The reaction was conducted using (–)-perillyl alcohol (22 μL) without modification from the general procedure to provide **10** as a light yellow oil.

Run 1: 13.0 mg (56% yield). Run 2: 15.8 mg (68% yield).

Purification: SiO_2 column; CH_2Cl_2

^{1}H NMR (300 MHz, $CDCl_3$) δ 5.68 (s, 1H), 3.98 (s, 2H), 2.10-2.06 (m, 1H), 2.00-1.95 (m, 2H), 1.83-1.76 (m, 1H), 1.48-1.33 (m, 2H), 1.26-1.13 (m, 1H), 0.93 (s, 3H), 0.89-0.79 (m, 1H), 0.28-0.15 (m, 4H).

$^{13}C\{^{1}H\}$ NMR (126 MHz, $CDCl_3$) δ 137.3, 123.1, 67.4, 42.2, 28.2, 26.7, 26.3, 19.2, 18.7, 12.4, 12.3.

HRMS (EI) calc. for $C_{11}H_{18}O^{+}$: m/z=166.1352, found: m/z=166.1352

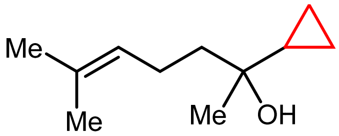

(11). The reaction was conducted using linalool (25 μL) without modification from the general procedure to provide **11** as a light yellow oil.

Run 1: 13.4 mg (57% yield). Run 2: 14.4 mg (61% yield).

Purification: SiO_2 column; pentane/CH_2Cl_2

^{1}H NMR (300 MHz, $CDCl_3$) δ 5.18-5.11 (m, 1H), 2.13 (q, J = 7.05, 2H), 1.69 (s, 3H), 1.63 (s, 3H), 1.59-1.53 (m, 2H), 1.11 (s, 3H), 1.09 (s, 1H), 0.95-0.86 (m, 1H), 0.39-0.25 (m, 4H).

$^{13}C\{^{1}H\}$ NMR (126 MHz, $CDCl_3$) δ 131.7, 124.6, 71.2, 42.9, 25.9, 22.8, 21.0, 17.7, 0.5.

HRMS (EI) calc. for $C_{11}H_{21}$: m/z=169.1593, found: m/z=169.1587

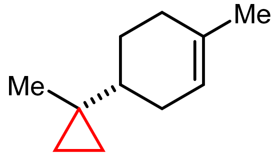

(12). The reaction was conducted using (+)-limonene (23 μL) without modification from the general procedure to provide **12** as a colorless oil.

Run 1: 17.0 mg (81% yield). Run 2: 16.6 mg (79% yield). 1.5 g scale: 1.49 g (90% yield)

Purification: SiO_2 column; pentane

^{1}H NMR (300 MHz, $CDCl_3$) δ 5.38 (s, 1H), 2.04-1.88 (m, 4H), 1.75-1.65 (m, 1H), 1.63 (s, 3H), 1.48-1.32 (m, 1H), 0.92 (s, 3H), 0.88-0.77 (m, 1H), 0.27-0.17 (m, 4H).

$^{13}C\{^{1}H\}$ NMR (126 MHz, $CDCl_3$) δ 133.9, 121.1, 42.1, 31.1, 29.7, 28.5, 26.7, 23.5, 19.3, 18.7, 12.4, 12.3.

HRMS (EI) calc. for $C_{11}H_{18}^{+}$: m/z=150.1403, found: m/z=150.1408

Gram-scale catalytic cyclopropanation of (*R*)-Limonene. In an N_2-filled glovebox, a 250-mL round-bottom flask was charged with $CoBr_2$ (144 mg, 0.66 mmol, 0.060 equiv), the $^{i\text{-Pr}}$PDI ligand (318 mg, 0.66 mmol, 0.060 equiv), THF (50 mL), and a magnetic stir bar. The catalyst mixture was allowed to stir at room temperature for 24 h. After this premixing period, (*R*)-limonene (1.5 g, 11 mmol, 1.0 equiv), CH_2Br_2 (2.85 g, 16.5 mmol, 1.5 equiv), and Zn powder (1.43 mg, 22 mmol, 2.0 equiv) were added. The reaction mixture was stirred at room temperature. After 24 h, the reaction mixture was concentrated under reduced pressure, and the crude residue was directly loaded onto a column for purification. (1.49 g, 90% yield)

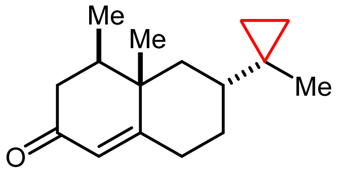

(13). The reaction was conducted using (+)-nootkatone (31 mg) without modification from the general procedure to provide **13** as an off-white solid.

Run 1: 27.3 mg (84% yield). Run 2: 29.9 mg (92% yield).

Purification: SiO_2 column; CH_2Cl_2

^{1}H NMR (300 MHz, $CDCl_3$) δ 5.74 (s, 1H), 2.46-2.31 (m, 2H), 2.27-2.18 (m, 2H), 2.07-1.94 (m, 1H), 1.90-1.74 (m, 2H), 1.40-1.30 (m, 1H), 1.28-1.17 (m, 1H), 1.14-1.10 (m, 1H), 1.01 (s, 3H), 0.98 (d, *J* = 6.79 Hz, 3H), 0.88 (s, 3H), 0.28-0.21 (m, 4H).

$^{13}C\{^{1}H\}$ NMR (126 MHz, $CDCl_3$) δ 199.7, 171.3, 124.6, 42.3, 42.1, 41.4, 40.6, 39.2, 33.2, 29.9, 18.9, 18.8, 16.9, 15.0, 13.0, 12.9.

HRMS (ESI) calc. for $C_{16}H_{25}O$: m/z=233.1906, found: m/z=233.1901

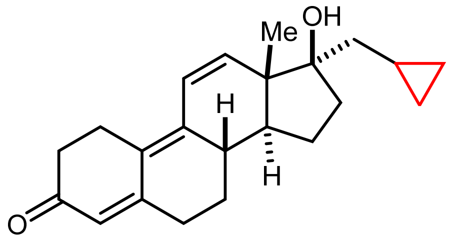

(14). The reaction was conducted using altrenogest (43 mg) without modification from the general procedure to provide **14** as a yellow oil.

Run 1: 37.2 mg (82% yield). Run 2: 39.1 mg (86% yield).

Purification: SiO_2 column; 3:2 EtOAc/hexane

^{1}H NMR (300 MHz, $CDCl_3$) δ 6.44 (d, *J* = 9.93 Hz, 1H), 6.29 (d, *J* = 10.02 Hz, 1H), 5.77 (s, 1H), 2.83-2.76 (m, 2H), 2.61-2.52 (m, 2H), 2.46 (t, *J* = 7.21 Hz, 3H), 2.31-2.21 (m, 1H), 2.10 (s, 1H),

1.94-1.86 (m, 1H), 1.80-1.63 (m, 3H), 1.55-1.47 (m, 2H), 1.39-1.21 (m, 2H), 1.02 (s, 3H), 0.90-0.79 (m, 1H), 0.54 (dd, *J* = 8.0, 1.25 Hz, 2H), 0.16-0.04 (m, 2H).

$^{13}C\{^{1}H\}$ NMR (126 MHz, $CDCl_3$) δ 199.2, 156.5, 142.0, 141.8, 126.9, 123.8, 123.6, 82.7, 49.0, 47.7, 42.7, 38.3, 36.7, 35.1, 31.5, 27.1, 24.3, 23.1, 16.5, 5.6, 4.5, 4.1.

HRMS (ESI) calc. for $C_{22}H_{29}O_2$: m/z=325.2168, found: m/z=325.2163

6. Procedures for Regioselective Monocyclopropanations of 1,3-Dienes

General Procedure. In an N_2-filled glovebox, a 3-mL vial was charged with $[^{i\text{-Pr}}PDI]CoBr_2$ (5.9 mg, 0.0084 mmol, 0.06 equiv), the substrate (0.14 mmol, 1.0 equiv), CH_2Br_2 (37 mg, 0.21 mmol, 1.5 equiv), Zn powder (18 mg, 0.28 mmol, 2.0 equiv), THF (1.0 mL), and a magnetic stir bar. The reaction was stirred at room temperature. After 24 h, the reaction mixture was concentrated under reduced pressure, and the crude residue was directly loaded onto a column for purification.

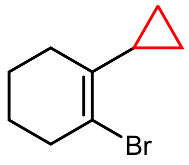

(15). The reaction was conducted using 1-bromo-2-vinylcyclohex-1-ene[8] (26 mg) without modification from the general procedure to provide **15** as a yellow oil.

Run 1: 17.7 mg (63% yield). Run 2: 18.0 mg (64% yield).

Purification: SiO_2 column; CH_2Cl_2

^{1}H NMR (300 MHz, $CDCl_3$) δ 2.54-2.49 (m, 2H), 2.06-1.97 (m, 1H), 1.68-1.66 (m, 4H), 1.64-1.57 (m, 2H), 0.68-0.51 (m, 4H)

^{13}C{^{1}H} NMR (126 MHz, $CDCl_3$) δ 134.6, 119.8, 37.0, 25.9, 24.8, 22.2, 17.0, 14.1, 3.9.

HRMS (ESI) calc. for $C_9H_{13}Br^+$: m/z=199.0121, found: m/z=199.0116

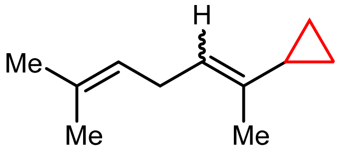

(16). The reaction was conducted using ocimene (2.3:1 *E/Z* ratio) (24 µL) without modification from the general procedure to provide **16** as a colorless oil.

Run 1: 16.6 mg (79% yield). Run 2: 17.5 mg (83% yield).

Purification: SiO_2 column; pentane

^{1}H NMR (300 MHz, $CDCl_3$) δ 5.18 (t, J = 7.21 Hz, 1H), 5.14-5.07 (m, 1H), 2.86-2.67 (m, 2H), 1.70-1.69 (m, 3H), 1.65-1.63 (m, 3H), 1.51-1.41 (m, 3H), 1.39-1.26 (m, 1H), 0.64-0.40 (m, 4H).

^{13}C{^{1}H} NMR (126 MHz, $CDCl_3$) δ 135.4, 134.6, 131.3, 124.5, 123.4, 123.3, 121.9, 27.0, 26.6, 25.7, 18.9, 18.7, 17.7, 13.8, 12.3, 4.2, 4.0.

HRMS (ESI) calc. for $C_{11}H_{17}$: m/z=149.1330, found: m/z=149.1324

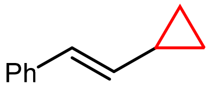

(17). The reaction was conducted using (*E*)-buta-1,3-dien-1-ylbenzene[9] (18 mg) without modification from the general procedure to provide **17** as a colorless oil.

Run 1: 17.0 mg (84% yield). Run 2: 17.4 mg (86% yield).

Purification: SiO_2 column; pentane

^{1}H NMR (300 MHz, $CDCl_3$) δ 7.33-7.14 (m, 5H), 6.49 (d, *J* = 15.8 Hz), 5.79-5.70 (m, 1H), 1.64-1.53 (m, 1H), 0.88-0.78 (m, 2H), 0.55-0.50 (m, 2H).

^{13}C{^{1}H} NMR (126 MHz, $CDCl_3$) δ 137.8, 134.9, 128.5, 127.4, 126.5, 125.6, 14.6, 7.3.

HRMS (CI) calc. for $C_{11}H_{12}^+$: m/z=144.0934, found: m/z=144.0932

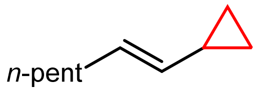

(18). The reaction was conducted using (*E*)-nona-1,3-diene[9] (17 mg) without modification from the general procedure to provide **18** as a colorless oil.

Run 1: 12.6 mg (65% yield). Run 2: 13.9 mg (72% yield).

Purification: SiO_2 column; pentane

^{1}H NMR (300 MHz, $CDCl_3$) δ 5.51 (dt, *J* = 6.76 Hz, 15.24 Hz, 1H), 4.95 (ddt, *J* = 15.2, 8.5, 1.5 Hz, 1H), 2.00-1.93 (m, 2H), 1.39-1.31 (m, 3H), 1.30-1.24 (m, 4H), 0.88 (t, *J* = 6.69 Hz, 3H), 0.68-0.61 (m, 2H), 0.33-0.28 (m, 2H).

^{13}C{^{1}H} NMR (126 MHz, $CDCl_3$) δ 133.6, 128.4, 32.5, 31.4, 29.4, 22.6, 14.1, 13.5, 6.3.

HRMS (CI) calc. for $C_{10}H_{18}^+$: m/z=138.1409, found: m/z=138.1406

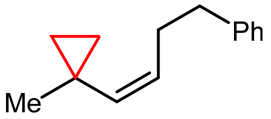

(19). The reaction was conducted using (Z)-(4-(1-methylcyclopropyl)but-3-en-1-yl)benzene[10] (24 mg) without modification from the general procedure to provide **19** as a colorless oil.

Run 1: 20.9 mg (80% yield). Run 2: 18.5 mg (71% yield).

Purification: SiO_2 column; pentane

^{1}H NMR (300 MHz, $CDCl_3$) δ 7.35-7.19 (m, 5H), 5.53 (d, *J* = 10.81 Hz, 1H), 5.42-5.34 (m, 1H), 2.73-2.68 (m, 2H), 2.62-2.54 (m, 2H), 1.14 (s, 3H), 0.54-0.43 (m, 4H).

^{13}C{^{1}H} NMR (126 MHz, $CDCl_3$) δ 142.2, 135.1, 131.1, 128.5, 128.3, 125.8, 36.1, 30.3, 25.0, 14.9, 14.6.

HRMS (APCI) calc. for $C_{16}H_{19}$: m/z=187.1481, found: m/z=187.1483

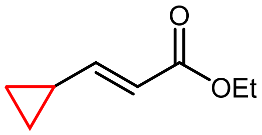

(20). The reaction was conducted using ethyl (*E*)-penta-2,4-dienoate[11] (18 mg) without modification from the general procedure to provide **20** as a colorless oil.

Run 1: 15.1 mg (77% yield). Run 2: 14.9 mg (76% yield).

Purification: SiO_2 column; CH_2Cl_2

^1H NMR (300 MHz, $CDCl_3$) δ 6.41 (dd, J = 10.07, 15.42 Hz, 1H), 5.88 (d, J = 15.42, 1H), 4.16, (q, J = 7.13 Hz, 2H), 1.62-1.50 (m, 1H), 1.27 (t, J = 7.15 Hz, 3H), 0.96-0.90 (m, 2H), 0.65-0.60 (m, 2H).

$^{13}C\{^1H\}$ NMR (126 MHz, $CDCl_3$) δ 166.7, 154.0, 118.2, 60.0, 14.3, 14.3, 8.6.

HRMS (CI) calc. for $C_8H_{12}O_2$: m/z=141.0910, found: m/z=141.0912

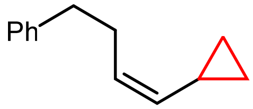

(21). The reaction was conducted using (Z)-hexa-3,5-dien-1-ylbenzene[12] (22 mg) without modification from the general procedure to provide **21** as a colorless oil.

Run 1: 22.9 mg (95% yield). Run 2: 21.9 mg (91% yield).

Purification: SiO_2 column; pentane

^1H NMR (300 MHz, $CDCl_3$) δ 7.34-7.19 (m, 5H), 5.42-5.33 (m, 1H), 4.79 (t, J = 9.83 Hz, 1H), 2.77-2.72 (m, 2H), 2.52 (q, J = 7.82 Hz, 2H), 1.59-1.46 (m, 1H), 0.75-0.69 (m, 2H), 0.34-0.29 (m, 2H).

$^{13}C\{^1H\}$ NMR (126 MHz, $CDCl_3$) δ 142.2, 134.6, 128.5, 128.3, 127.1, 125.8, 36.1, 29.5, 9.7, 6.9.

HRMS (EI) calc. for $C_{13}H_{16}^+$: m/z=172.1247, found: m/z=172.1242

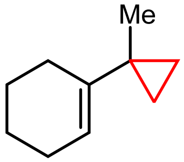

(22). The reaction was conducted using 1-(prop-1-en-2-yl)cyclohex-1-ene[13] (17 mg) without modification from the general procedure to provide **22** as a colorless oil.

Run 1: 12.0 mg (63% yield). Run 2: 13.5 mg (71% yield).

Purification: SiO_2 column; pentane

^1H NMR (300 MHz, $CDCl_3$) δ 5.50-5.48 (m, 1H), 2.02-1.89 (m, 4H), 1.64-1.50 (m, 4H), 1.13 (s, 3H), 0.58-0.55 (m, 2H), 0.34-0.31 (m, 2H).

$^{13}C\{^1H\}$ NMR (126 MHz, $CDCl_3$) δ 141.2, 120.1, 25.9, 25.3, 23.9, 23.1, 22.6, 21.3, 12.5.

HRMS (EI) calc. for $C_{10}H_{15}$: m/z=135.1168, found: m/z=135.1165

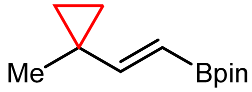

(23). The reaction was conducted using (E)-4,4,5,5-tetramethyl-2-(3-methylbuta-1,3-dien-1-yl)-1,3,2-dioxaborolane[14] (27 mg) without modification from the general procedure to provide **23** as a clear oil.

Run 1: 25.3 mg (87% yield). Run 2: 25.1 mg (86% yield).

Purification: SiO_2 column; CH_2Cl_2

^{1}H NMR (300 MHz, $CDCl_3$) δ 6.16 (d, *J* = 18.1 Hz, 1H), 5.37 (d, *J* = 18.1 Hz, 1H), 1.25 (s, 12H), 1.17, (s, 3H), 0.74-0.64, (m, 4H).

$^{13}C\{^{1}H\}$ NMR (126 MHz, $CDCl_3$) δ 161.9, 82.9, 24.8, 20.2, 19.7, 16.2.

^{11}B NMR (96 MHz, $CDCl_3$): δ 30.0.

HRMS (ESI) calc. for $C_{12}H_{21}BO_2^+$: m/z=208.1749, found: m/z=208.1744

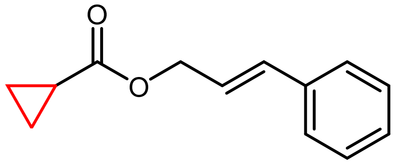

The reaction was conducted using cinnamyl acrylate[15] without modification from the general procedure to provide product **S1** as a yellow oil.

Run 1: 26.3 mg (93% yield). Run 2: 24.3 mg (86% yield).

Purification: SiO_2 column; CH_2Cl_2

^{1}H NMR (300 MHz, $CDCl_3$): 7.42-7.39 (m, 2H), 7.36-7.33 (m, 2H), 7.31-7.27 (m, 1H), 6.67 (d, *J* = 15.6 Hz, 1H), 6.31 (dt, *J* = 6.42, 15.87 Hz, 1H), 4.75 (dd, *J* = 1.26, 6.42 Hz, 2H), 1.71-1.63 (m, 1H), 1.07-1.02 (m, 2H), 0.92-0.86 (m, 2H),

$^{13}C\{^{1}H\}$ NMR (126 MHz, $CDCl_3$):174.7, 136.3, 134.0, 128.6, 128.0, 126.6, 123.4, 65.1, 12.9, 8.6

HRMS (EI) calc. for $C_{13}H_{14}O_2^+$: m/z= 202.0988, found: m/z 202.0994

7. CV Data

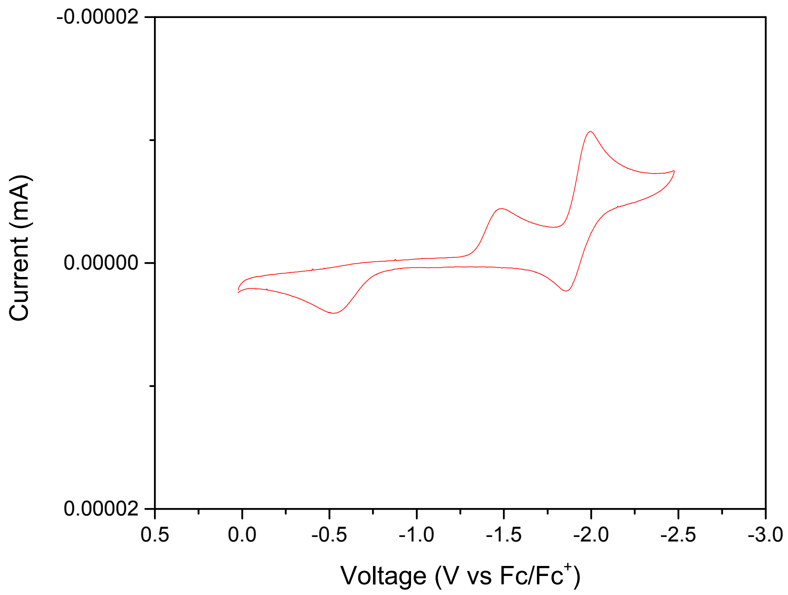

Figure S9. Cyclic voltammetry data for **1** (0.3 M [*n*-Bu_4N][PF_6] supporting electrolyte in THF, glassy carbon working electrode, 100 mV/s scan rate, N_2 atmosphere).

8. Synthesis and Characterization of the Co/Zn Complex 31

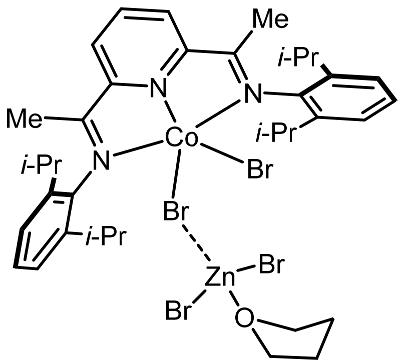

[$^{i\text{-Pr}}$PDI]CoBr$_2$Zn(THF/Et$_2$O)Br$_2$ (31). In an N_2-filled glovebox, a 20-mL vial was charged with [$^{i\text{-Pr}}$PDI]CoBr$_2$ (20 mg, 0.029 mmol, 1.0 equiv), $ZnBr_2$ (6.5 mg, 0.029 mmol, 1.0 equiv), THF (10-mL) and a magnetic stir bar. The reaction mixture was stirred at room temperature for 1 h to ensure complete complete dissolution and consumption of $ZnBr_2$. The reaction mixture was concentrated to dryness under reduced pressure. Ether was added to the residue, and the mixture was filtered through a glass fiber pad to produce a green solution. Slow-cooling of the solution (–30 °C) produced dark-green crystals (7 mg, 0.007 mmol, 24% yield). Single crystals obtained by this procedure were suitable for XRD analysis. Complex **31** is NMR silent.

Anal. Calc. for **31** ($C_{37}H_{51}Br_4CoN_3OZn$): C 44.54%, H 5.15%, N 4.21%; found: C 44.22%, H 5.24%, N 4.14%. In the solid state structure, there is a solvent molecule bound to Zn that is disordered between Et_2O and THF. The calculated elemental analysis is shown assuming 100% THF; however, the found values are also adequately modeled using 100% Et_2O due to the similarity in the elemental composition of the two solvents.

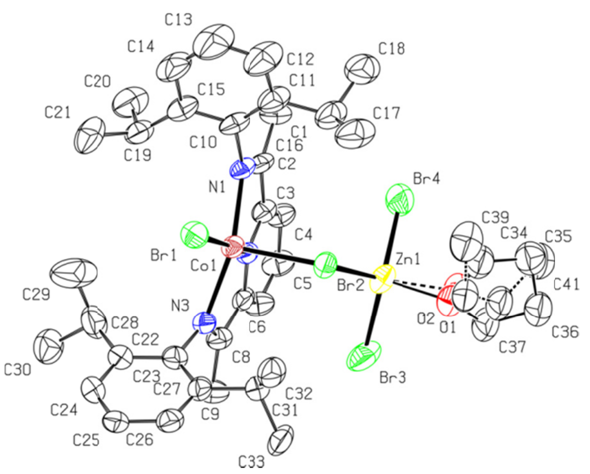

JJWIV_142_CoBr2PDI_0m

Crystal data	
Chemical formula	$C_{37}H_{51.88}Br_4CoN_3OZn$
M_r	998.64
Crystal system, space group	Orthorhombic, $P2_12_12_1$
Temperature (K)	150
a, *b*, *c* (Å)	12.6608 (8), 17.5590 (13), 18.5345 (14)
V (Å^3)	4120.4 (5)
Z	4
Radiation type	Cu *K*α
μ (mm^{-1})	8.65
Crystal size (mm)	0.23 × 0.13 × 0.10
Data collection	
Diffractometer	Bruker AXS D8 Quest CMOS diffractometer
Absorption correction	Multi-scan *SADABS* 2016/2: Krause, L., Herbst-Irmer, R., Sheldrick G.M. & Stalke D., J. Appl. Cryst. 48 (2015) 3-10
T_{min}, T_{max}	0.429, 0.753
No. of measured, independent and observed [$I > 2\sigma(I)$] reflections	23175, 7493, 6130

R_{int}	0.064
Refinement	
$R[F^2 > 2\sigma(F^2)]$, $wR(F^2)$, S	0.048, 0.123, 1.02
No. of reflections	7493
No. of parameters	485
No. of restraints	131
H-atom treatment	H-atom parameters constrained
$\Delta\rho_{max}$, $\Delta\rho_{min}$ (e Å^{-3})	1.23, −0.96
Absolute structure	Flack x determined using 2262 quotients [(I+)-(I-)]/[(I+)+(I-)] (Parsons, Flack and Wagner, Acta Cryst. B69 (2013) 249-259).
Flack parameter	−0.011 (4)

A THF and an ether molecule are disordered at a Zn coordinated site. Equivalent bonds in the disordered moieties were restrained to have similar bond distances, and the ether CH_2-CH_3 bonds were restrained to 1.54(2) Angstrom. Uij components of ADPs for disordered atoms closer to each other than 1.7 Angstrom were restrained to be similar. Subject to these conditions the occupancy ratio refined to 0.558(13) to 0.442(13) in favor of THF.

9. Mechanistic Experiments

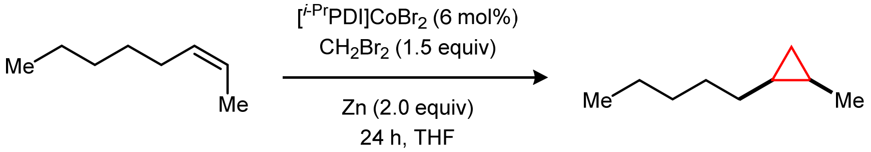

Stereospecificity. In an N_2-filled glovebox, a 3-mL vial was charged with [$^{i\text{-Pr}}$PDI]CoBr$_2$ (5.9 mg, 0.0084 mmol, 0.06 equiv), *cis*-2-octene (0.14 mmol, 1.0 equiv), CH_2Br_2 (37 mg, 0.21 mmol, 1.5 equiv), Zn powder (18 mg, 0.28 mmol, 2 equiv), mesitylene as an internal standard, THF (1 mL), and a magnetic stir bar. The reaction mixture was stirred at room temperature. After 24 h, CH_2Cl_2 was added to dilute the solution, and an aliquot of the mixture was removed and analyzed by GC. (95% yield, >50:1 E/Z ratio)

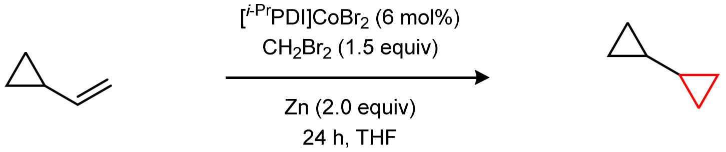

Radical Clock Experiment. In an N_2-filled glovebox, a 3-mL vial was charged with [$^{i\text{-Pr}}$PDI]CoBr$_2$ (5.9 mg, 0.0084 mmol, 0.06 equiv), vinylcyclopropane[16] (0.14 mmol, 1.0 equiv), CH_2Br_2 (37 mg, 0.21 mmol, 1.5 equiv), Zn powder (18 mg, 0.28 mmol, 2.0 equiv), mesitylene as an internal standard, THF (1.0 mL), and a magnetic stir bar. The reaction mixture was stirred at room temperature. After 24 h, an aliquot was removed and analyzed by ^1H NMR spectroscopy. No ring-opened product was observed by the limit of detection in the NMR.

NMR Yields: Run 1: 70% yield. Run 2: 72% yield.

^1H NMR (300 MHz, $CDCl_3$): 0.81-0.70 (m, 2H), 0.32-0.23 (m, 4H), 0.02-(-0.04) (m, 4H).

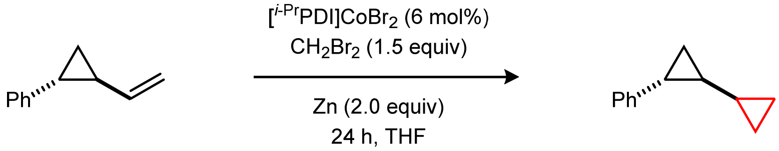

Radical Clock Experiment. In an N_2-filled glovebox, a 3-mL vial was charged with [$^{i\text{-Pr}}$PDI]CoBr$_2$ (5.9 mg, 0.0084 mmol, 0.06 equiv), (2-vinylcyclopropyl)benzene[17] (20 mg, 0.14 mmol, 1.0 equiv), CH_2Br_2 (37 mg, 0.21 mmol, 1.5 equiv), Zn powder (18 mg, 0.28 mmol, 2.0 equiv), THF (1.0 mL), and a magnetic stir bar. The reaction was stirred at room temperature. After 24 h, the reaction mixture was concentrated under reduced pressure. The crude residue directly purified by column chromatography to provide **29** as a colorless oil.

Run 1: 21.5 mg (97% yield). Run 2: 19.3 mg (87% yield).

Purification: SiO_2 column; pentane

^{1}H NMR (300 MHz, $CDCl_3$) δ 7.31-7.23 (m, 2H), 7.17-7.11 (m, 1H), 7.07-7.04 (m, 2H), 1.71-1.65 (m, 1H), 1.21-1.13 (m, 1H), 1.02-0.92 (m, 1H), 0.85-0.74 (m, 2H), 0.50-0.37 (m, 2H), 0.24-0.11 (m, 2H).

$^{13}C\{^{1}H\}$ NMR (126 MHz, $CDCl_3$) δ 143.7, 128.2, 125.6, 125.2, 25.4, 21.7, 13.8, 12.4, 3.4, 2.6.

HRMS (EI) calc. for $C_{12}H_{14}^{+}$: m/z=158.1090, found: m/z=158.1085

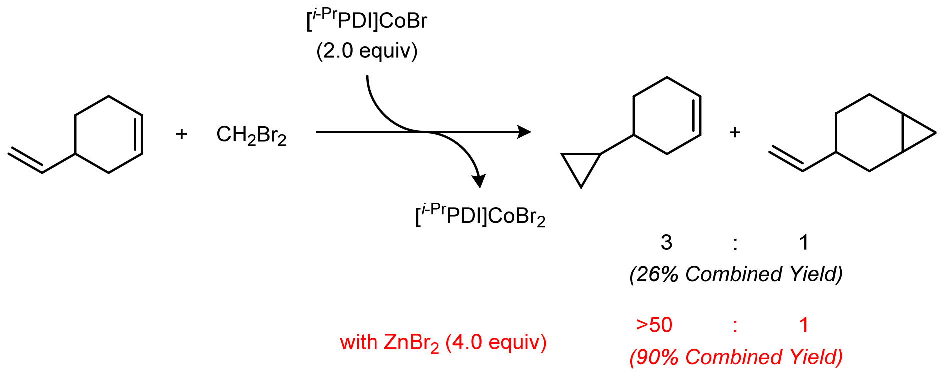

Stoichiometric Cyclopropanation with [$^{i\text{-Pr}}$PDI]CoBr. In an N_2-filled glovebox, a 3-mL vial was charged with [$^{i\text{-Pr}}$PDI]CoBr (5.2 mg, 0.0084 mmol, 0.06 equiv), 4-vinylcyclohexene (0.14 mmol, 1 equiv), CH_2Br_2 (27 mg, 0.15 mmol, 1.1 equiv), mesitylene as an internal standard, THF (1 mL), and a magnetic stir bar. The reaction mixture was stirred at room temperature. After 24 h, CH_2Cl_2 was added to dilute the solution and an aliquot was removed and analyzed by GC. The same procedure was repeated in the presence of $ZnBr_2$ (3.8 mg, 0.017 mmol, 0.12 equiv).

The yields were determined assuming that two equivalents of the [$^{i\text{-Pr}}$PDI]CoBr complex are required for each equivalent of cyclopropane that is generated.

10. NMR Spectra

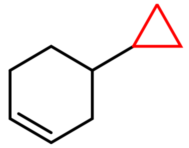

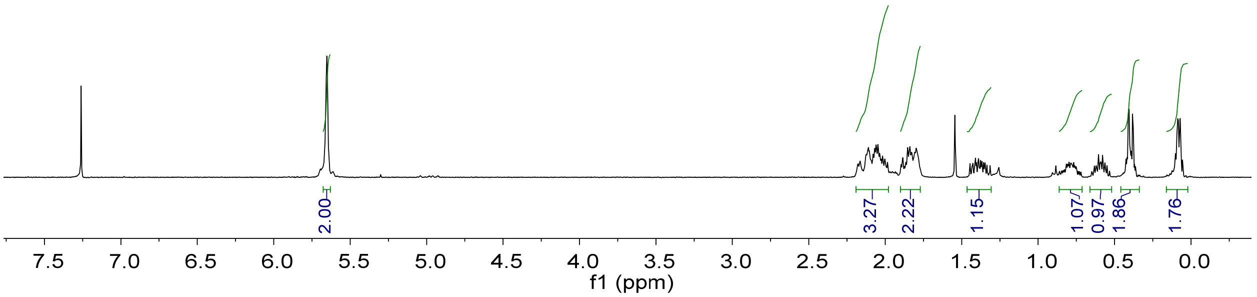

Figure S10. ^{1}H NMR spectrum for **4** ($CDCl_3$, 295 K).

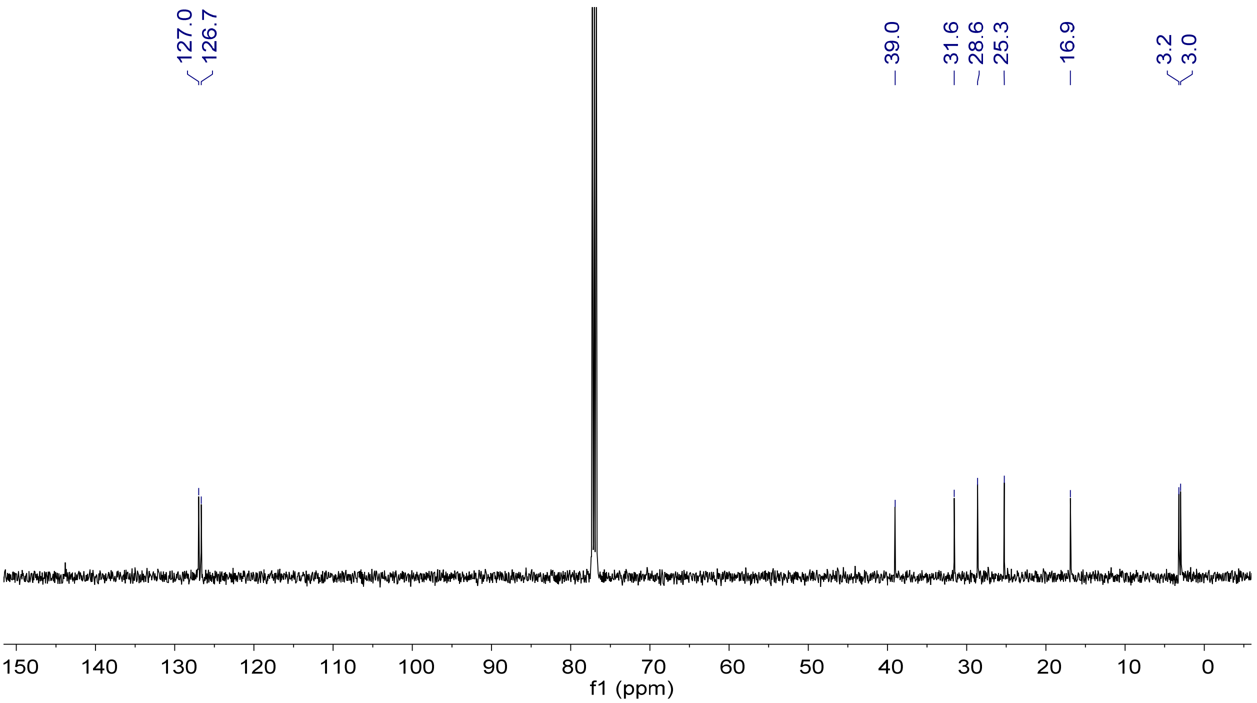

Figure S11. ^{13}C NMR spectrum for **4** ($CDCl_3$, 295 K).

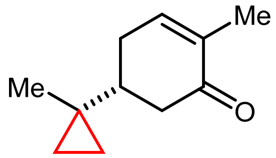

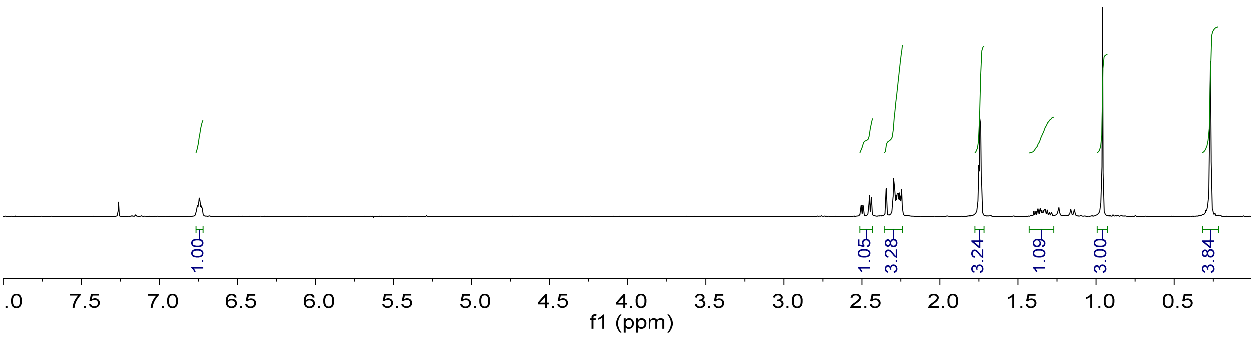

Figure S12. ^{1}H NMR spectrum for **6** ($CDCl_3$, 295 K).

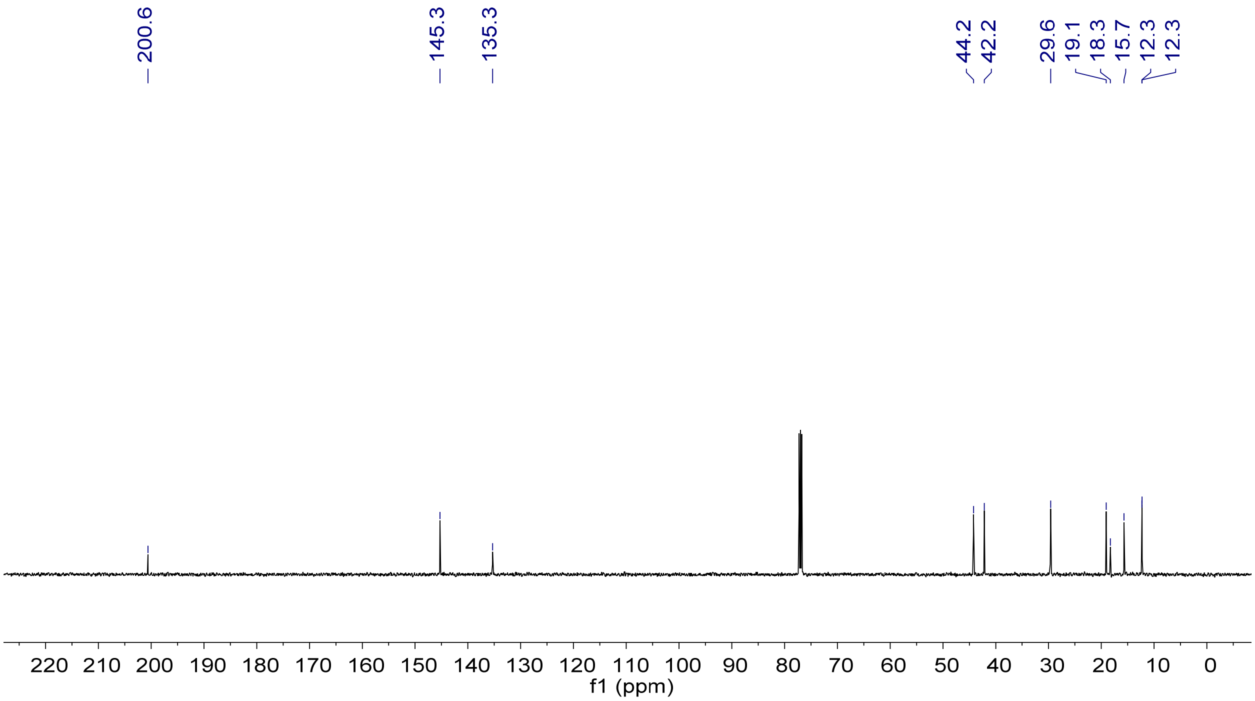

Figure S13. ^{13}C NMR spectrum for **6** ($CDCl_3$, 295 K).

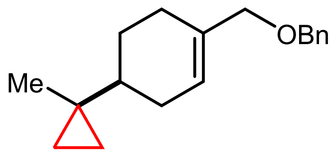

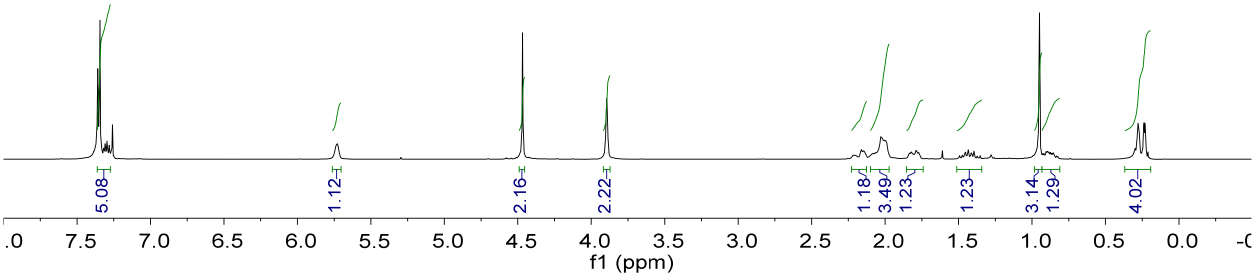

Figure S14. ^{1}H NMR spectrum for **7** ($CDCl_3$, 295 K).

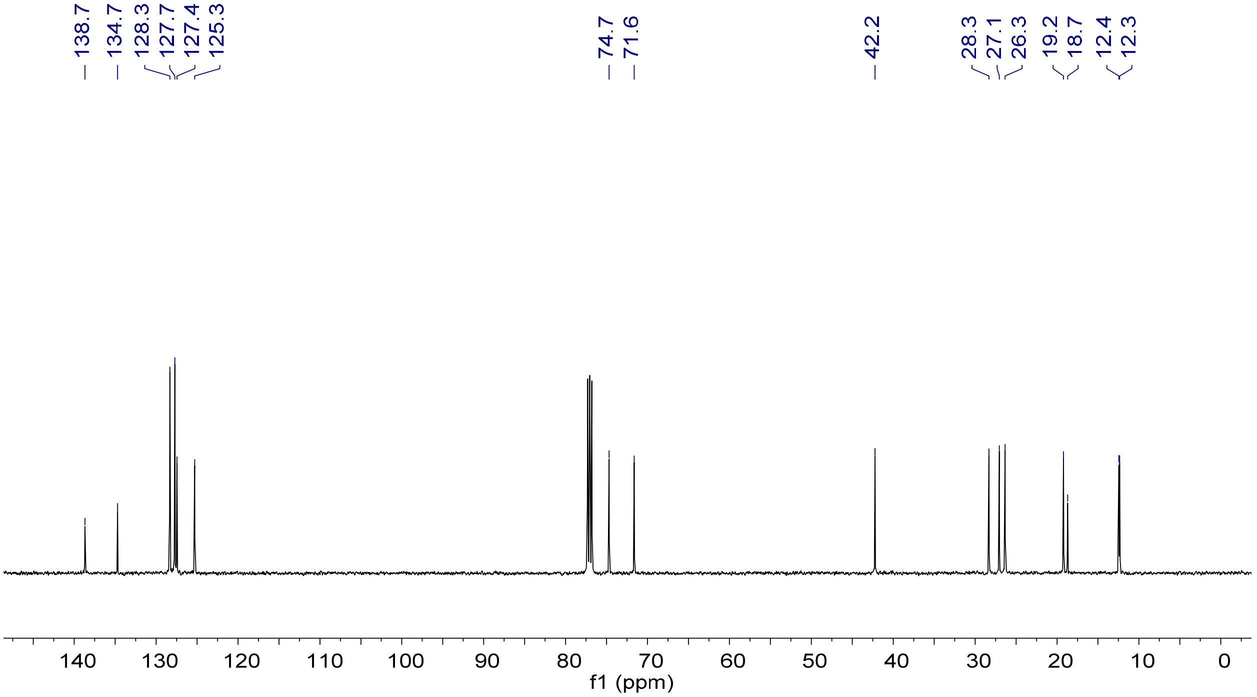

Figure S15. ^{13}C NMR spectrum for **7** ($CDCl_3$, 295 K).

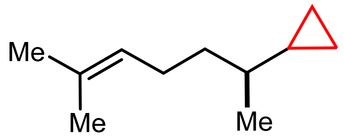

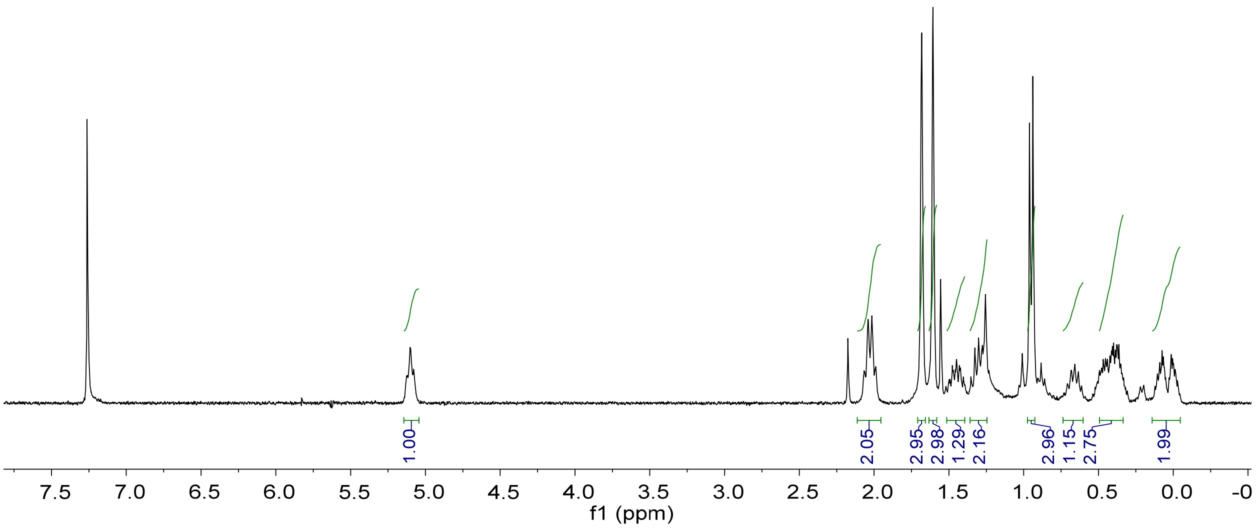

Figure S16. ^{1}H NMR spectrum for **8** ($CDCl_3$, 295 K).

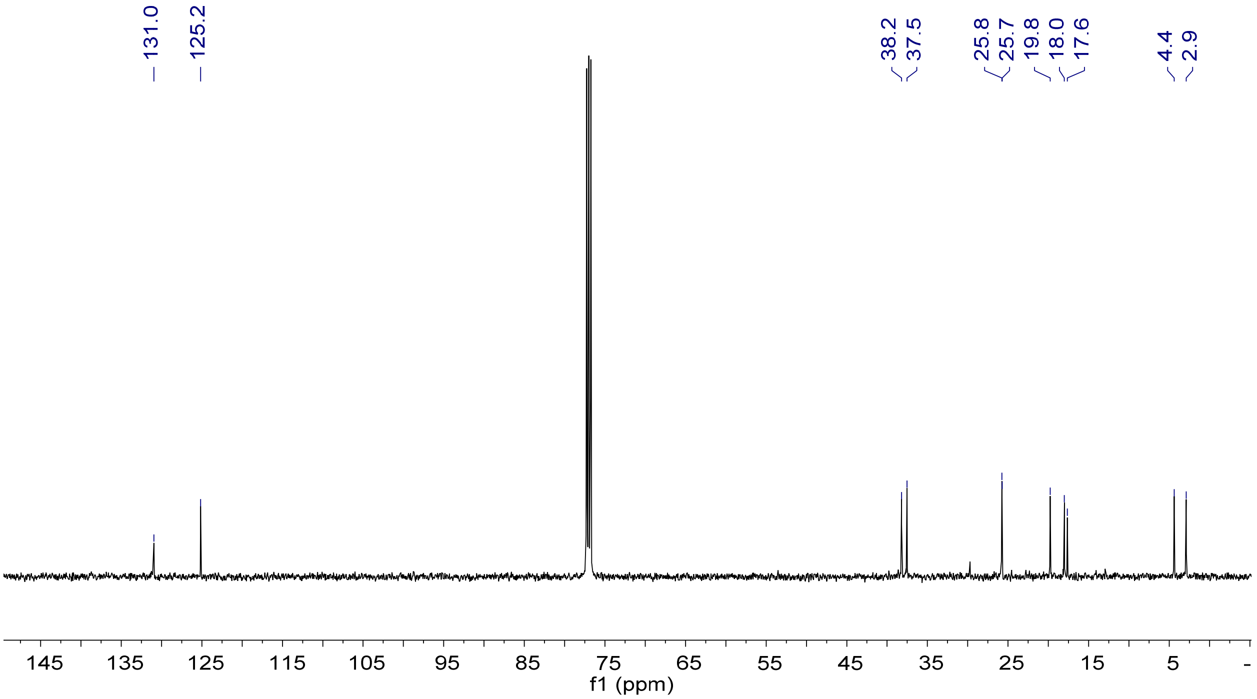

Figure S17. ^{13}C NMR spectrum for **8** ($CDCl_3$, 295 K).

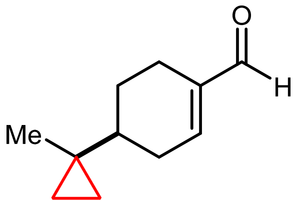

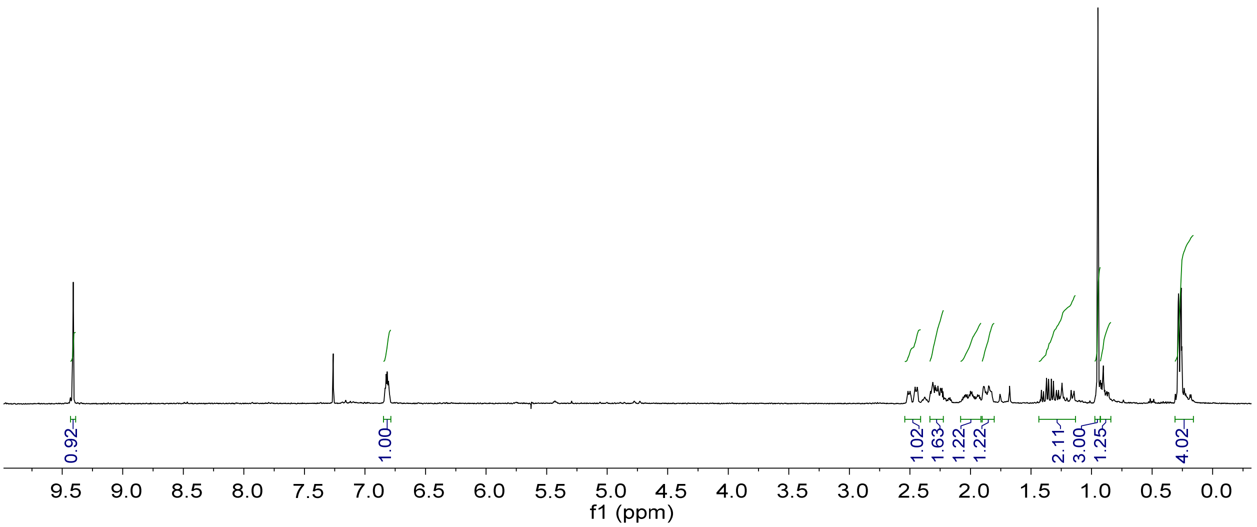

Figure S18. ^{1}H NMR spectrum for **9** ($CDCl_3$, 295 K).

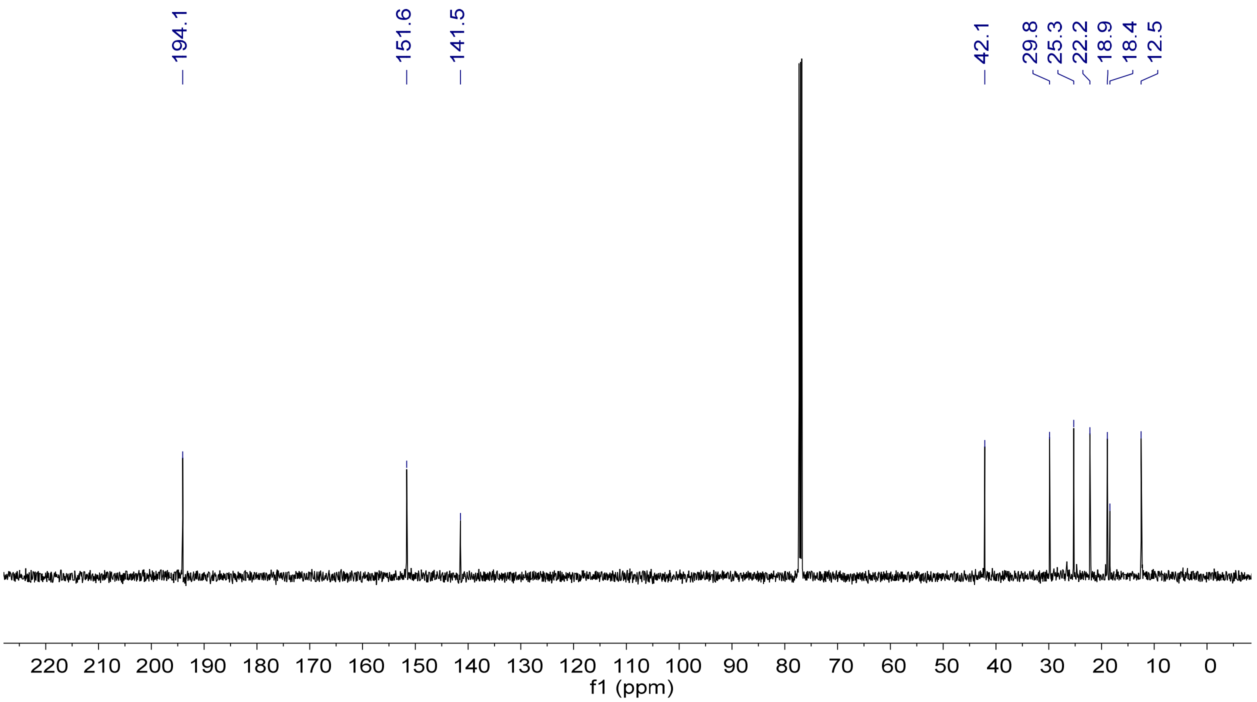

Figure S19. ^{13}C NMR spectrum for **9** ($CDCl_3$, 295 K).

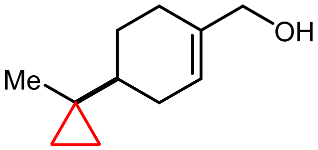

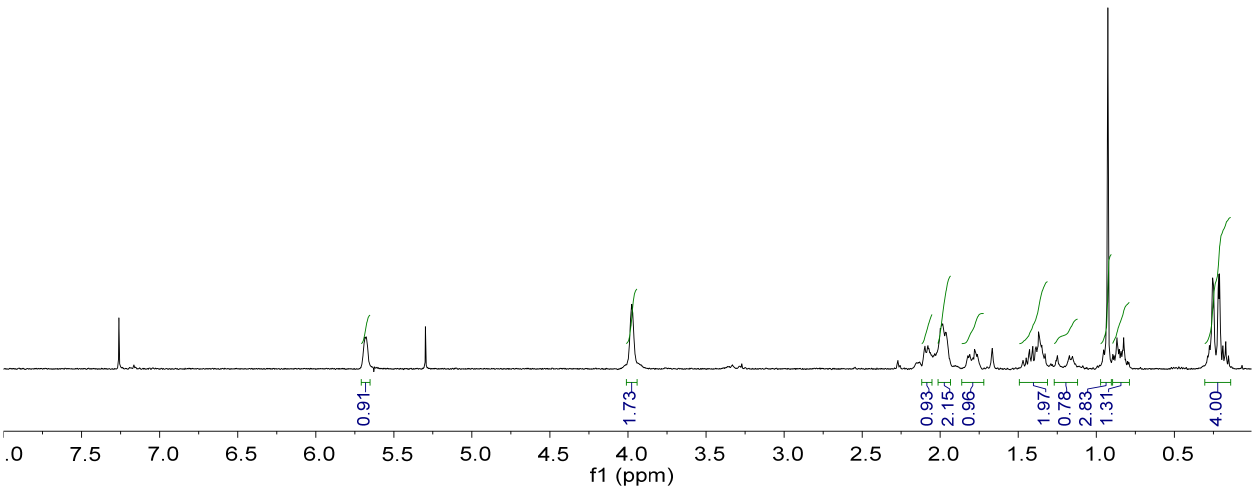

Figure S20. ^{1}H NMR spectrum for **10** ($CDCl_3$, 295 K).[18]

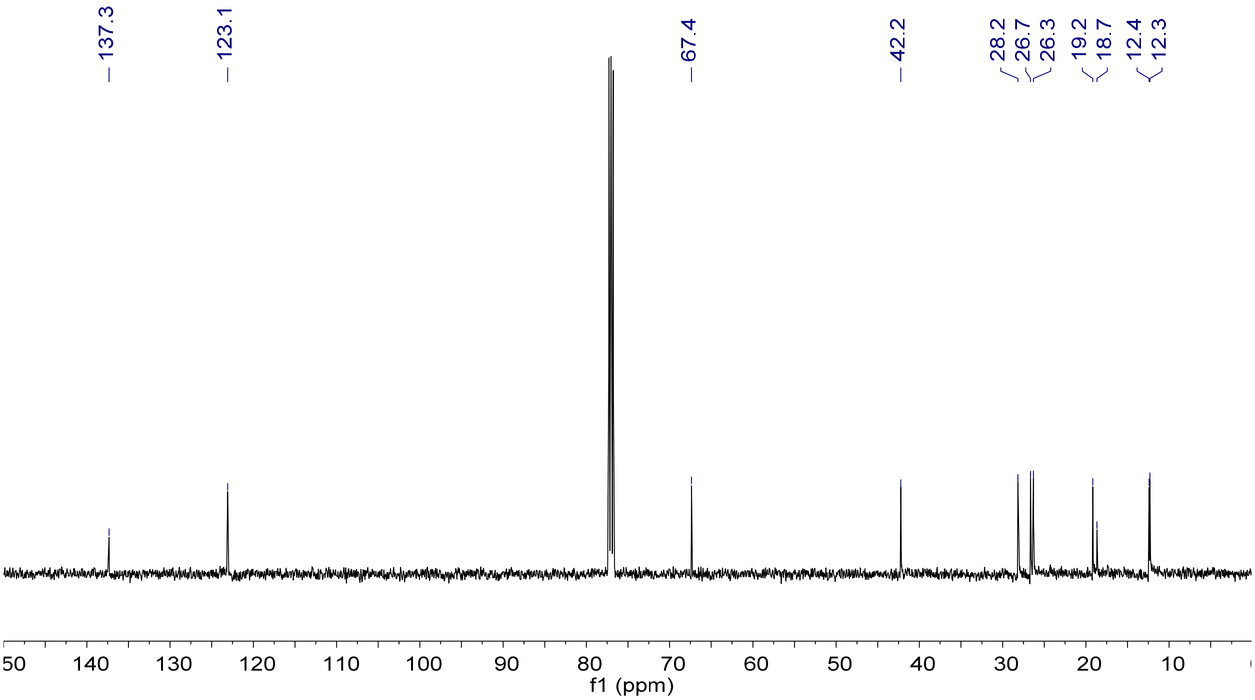

Figure S21. ^{13}C NMR spectrum for **10** ($CDCl_3$, 295 K).

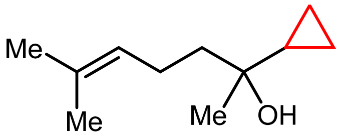

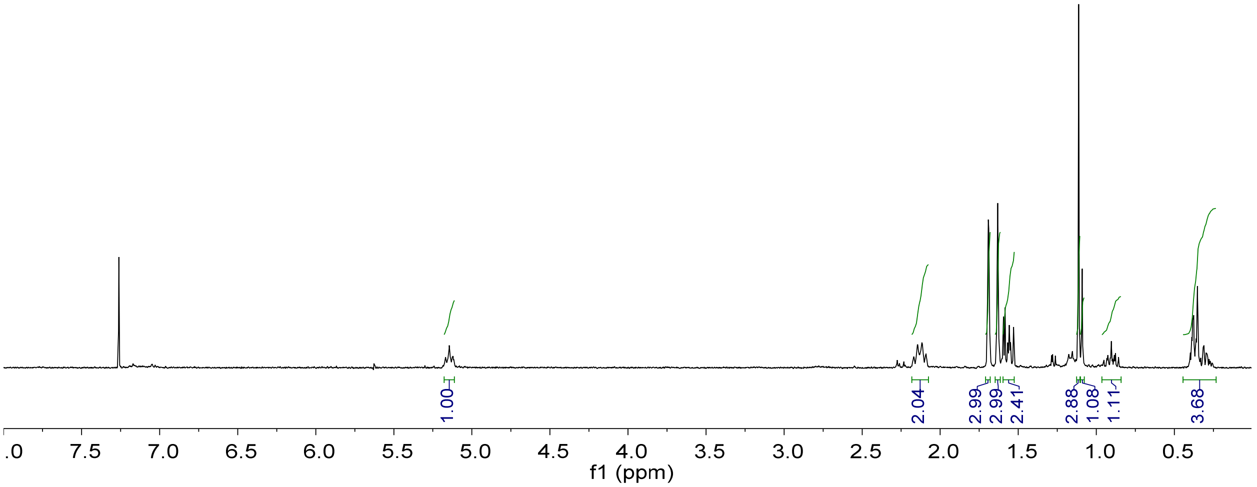

Figure S22. ^{1}H NMR spectrum for **11** ($CDCl_3$, 295 K).

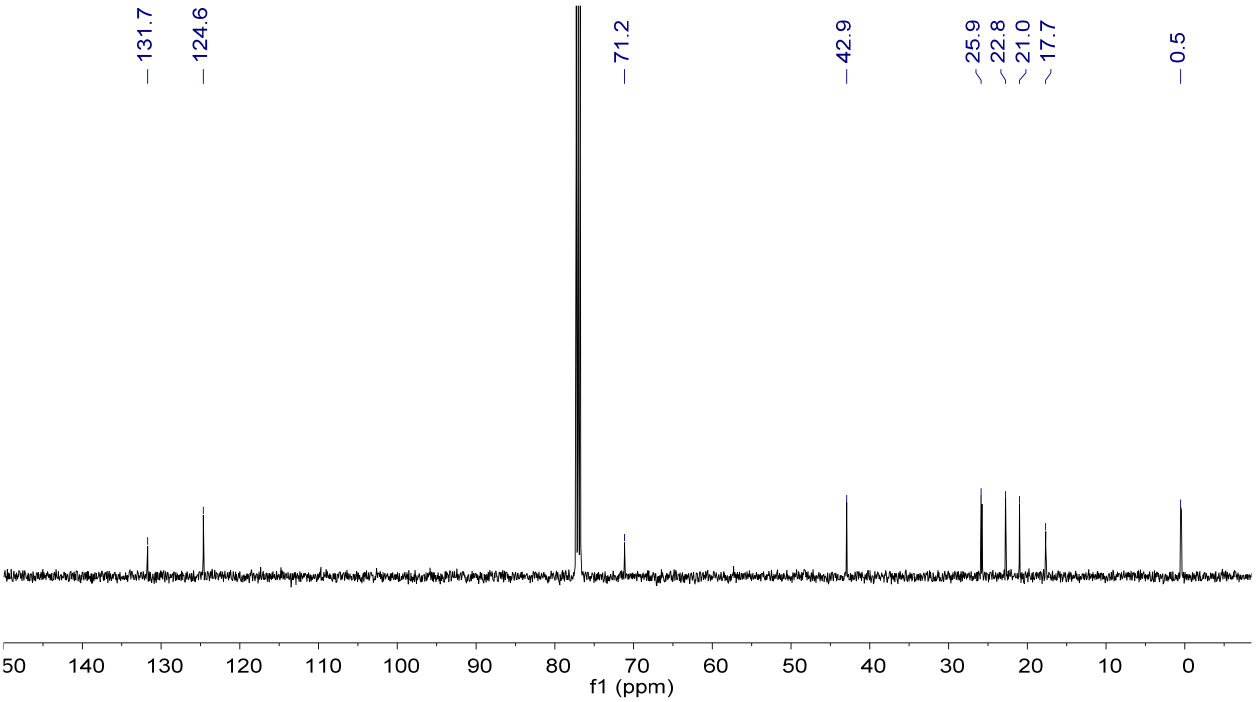

Figure S23. ^{13}C NMR spectrum for **11** ($CDCl_3$, 295 K).

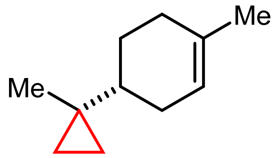

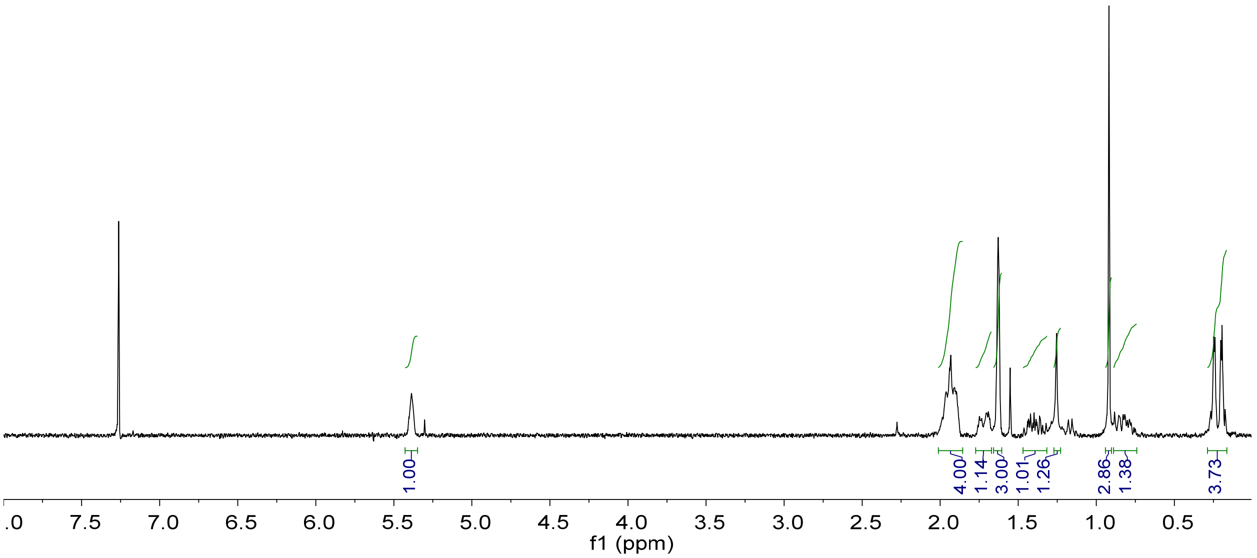

Figure S24. ^{1}H NMR spectrum for **12** ($CDCl_3$, 295 K).

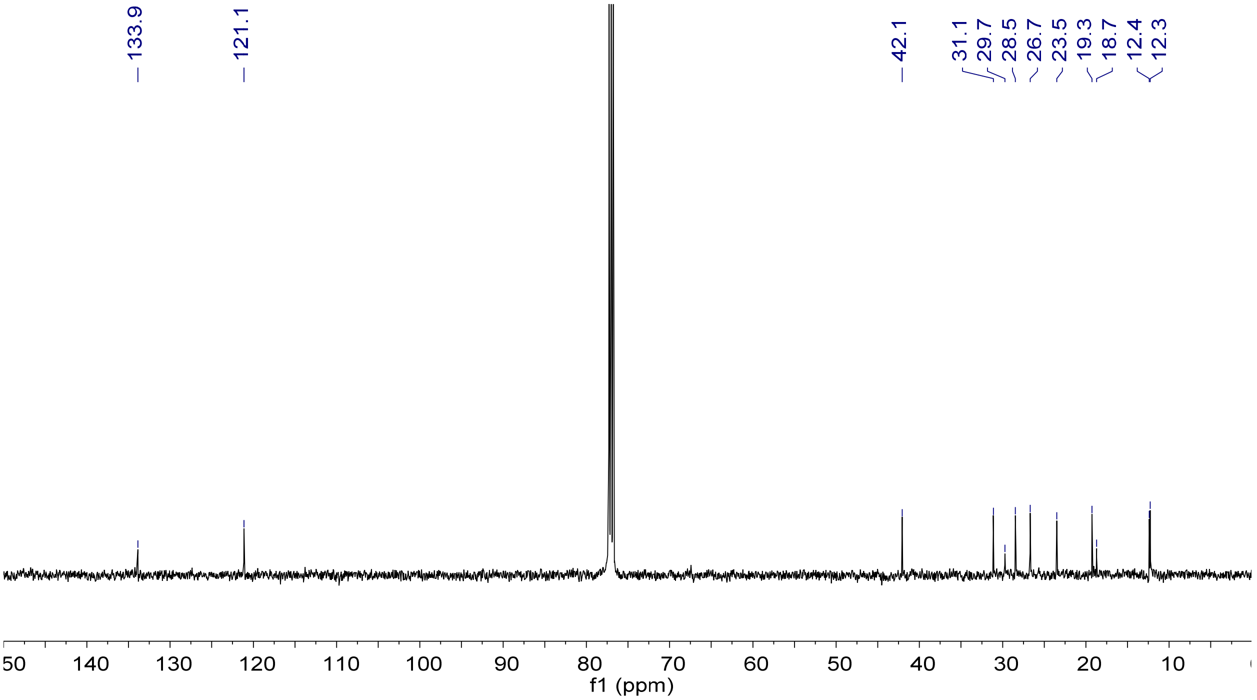

Figure S25. ^{13}C NMR spectrum for **12** ($CDCl_3$, 295 K).

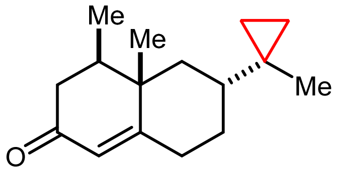

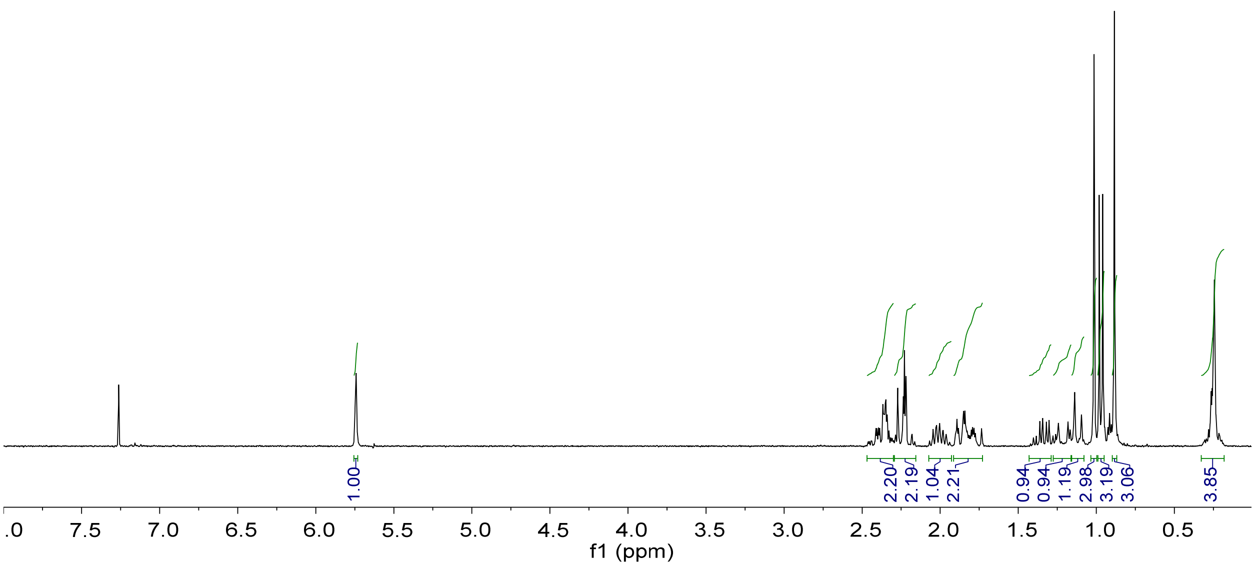

Figure S26. ^{1}H NMR spectrum for **13** ($CDCl_3$, 295 K).

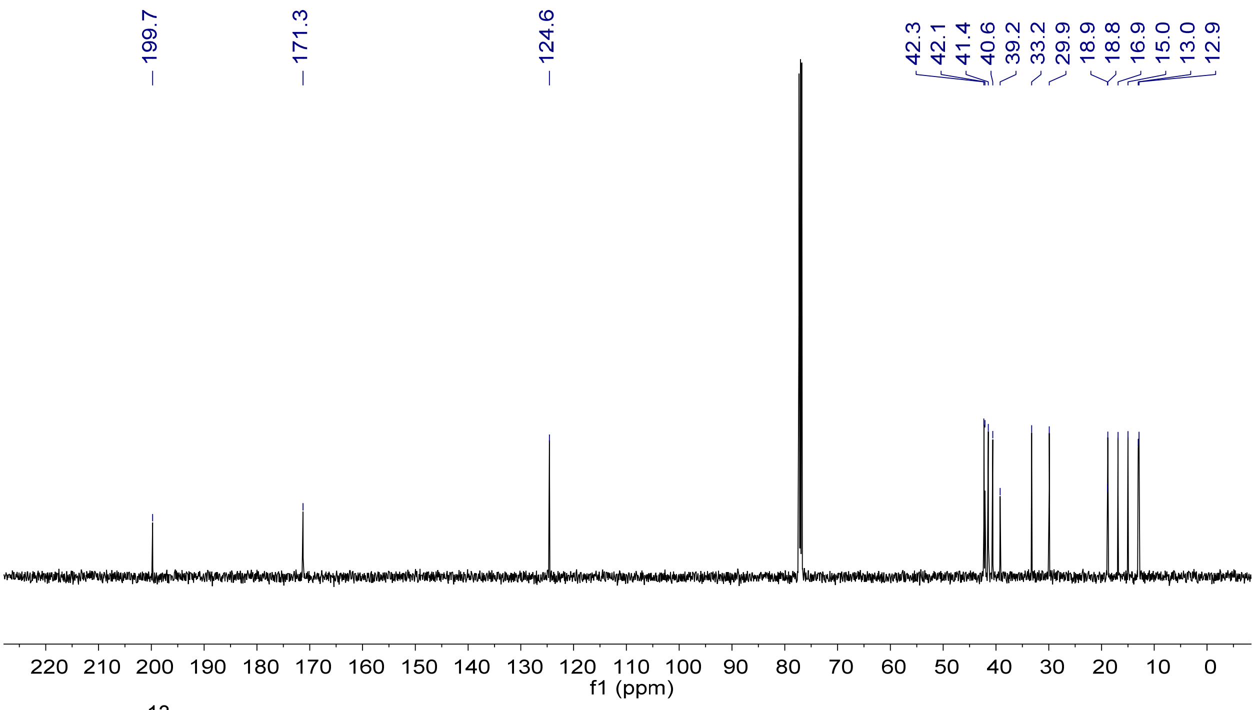

Figure S27. ^{13}C NMR spectrum for **13** ($CDCl_3$, 295 K).

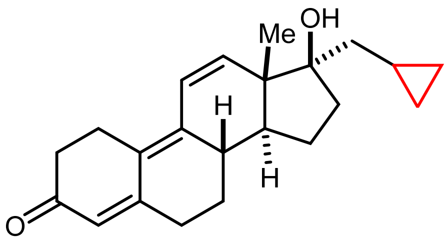

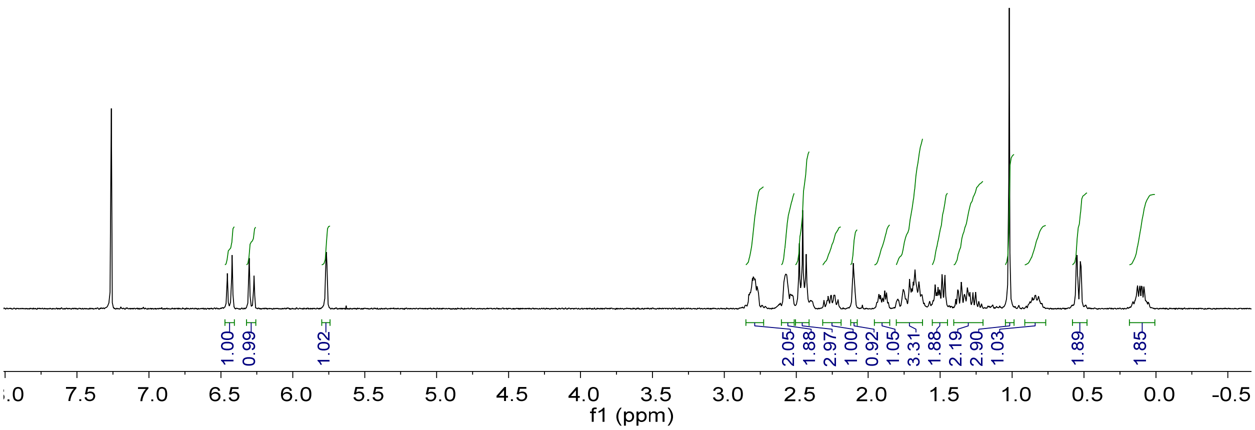

Figure S28. ^{1}H NMR spectrum for **14** ($CDCl_3$, 295 K).

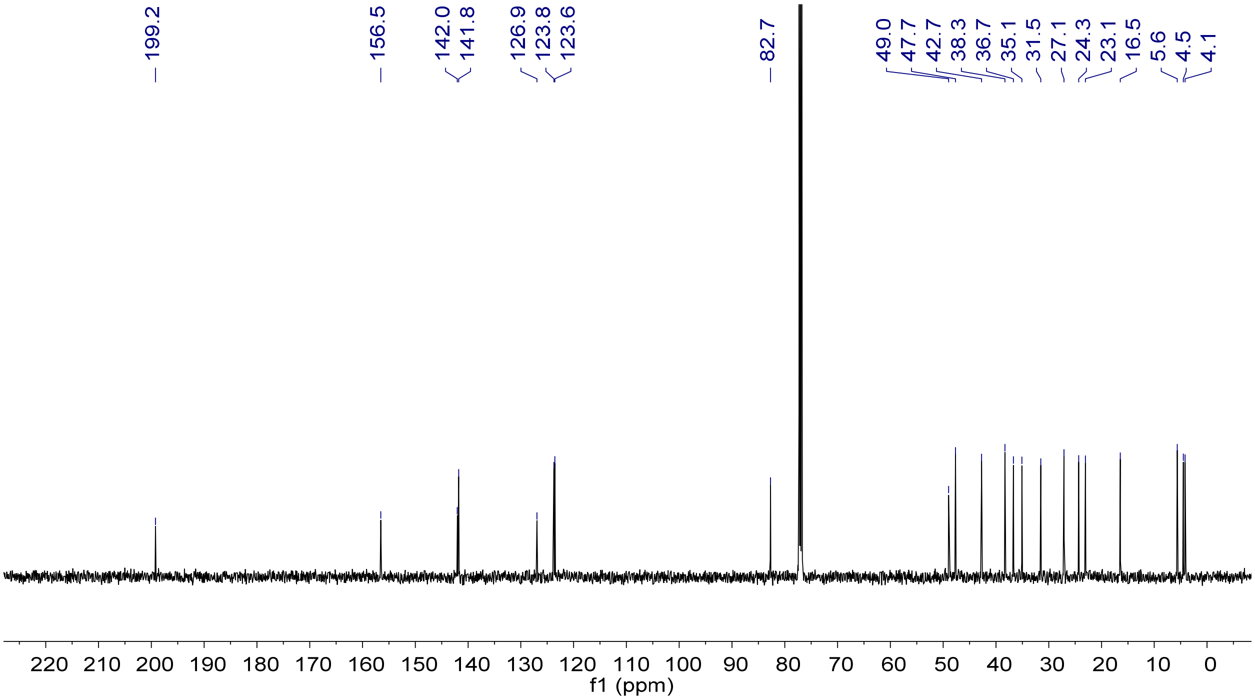

Figure S29. ^{13}C NMR spectrum for **14** ($CDCl_3$, 295 K).

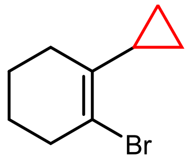

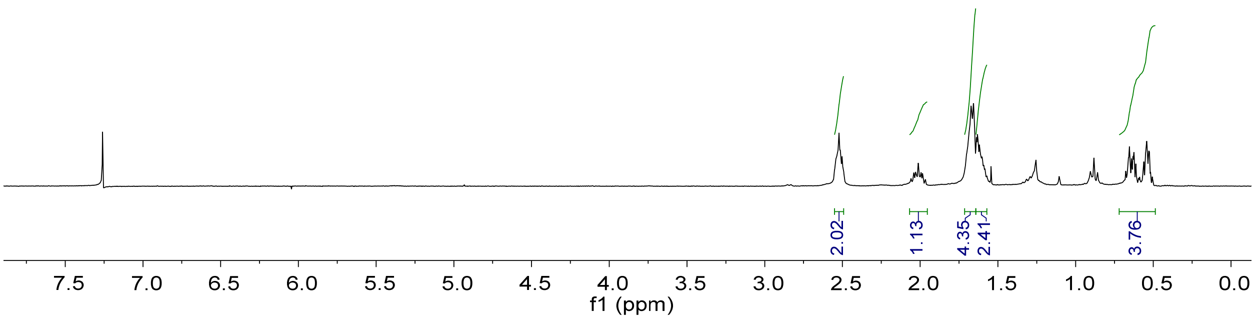

Figure S30. ^{1}H NMR spectrum for **15** ($CDCl_3$, 295 K).

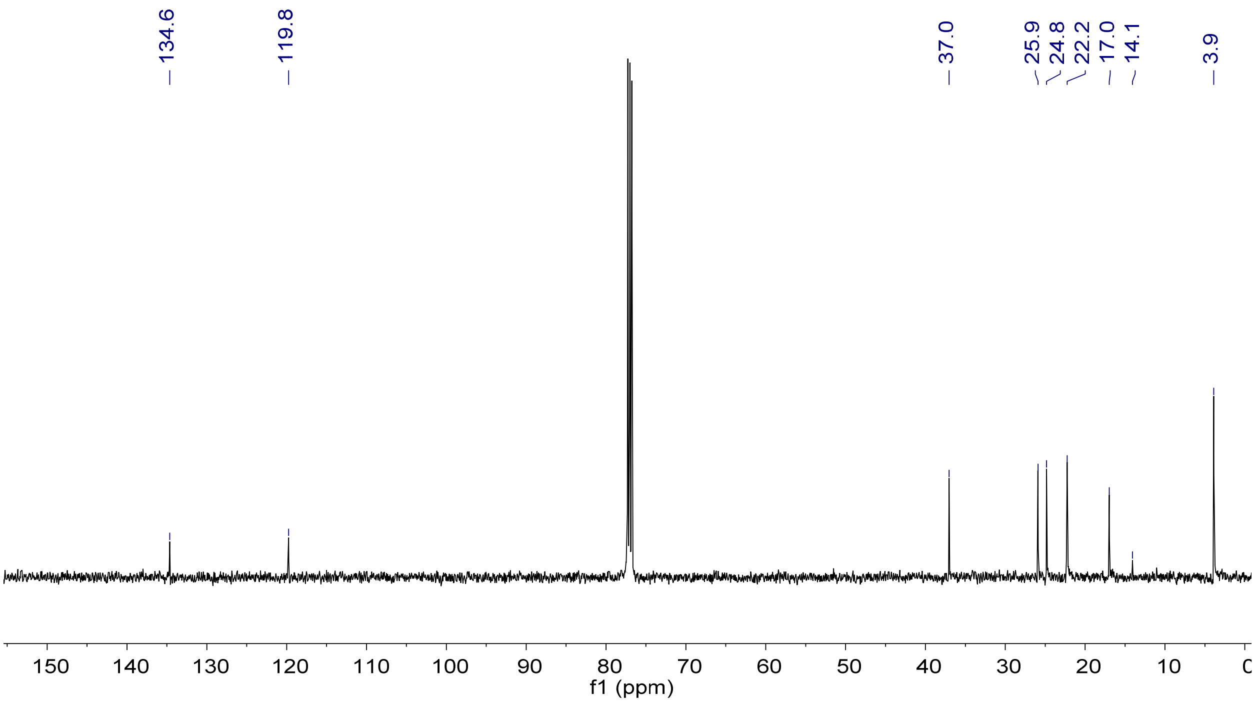

Figure S31. ^{13}C NMR spectrum for **15** ($CDCl_3$, 295 K).

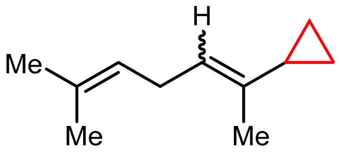

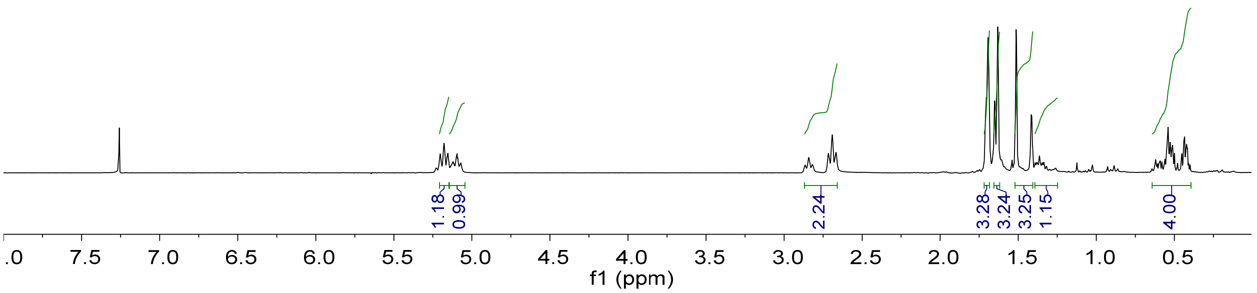

Figure S32. ^{1}H NMR spectrum for **16** ($CDCl_3$, 295 K).

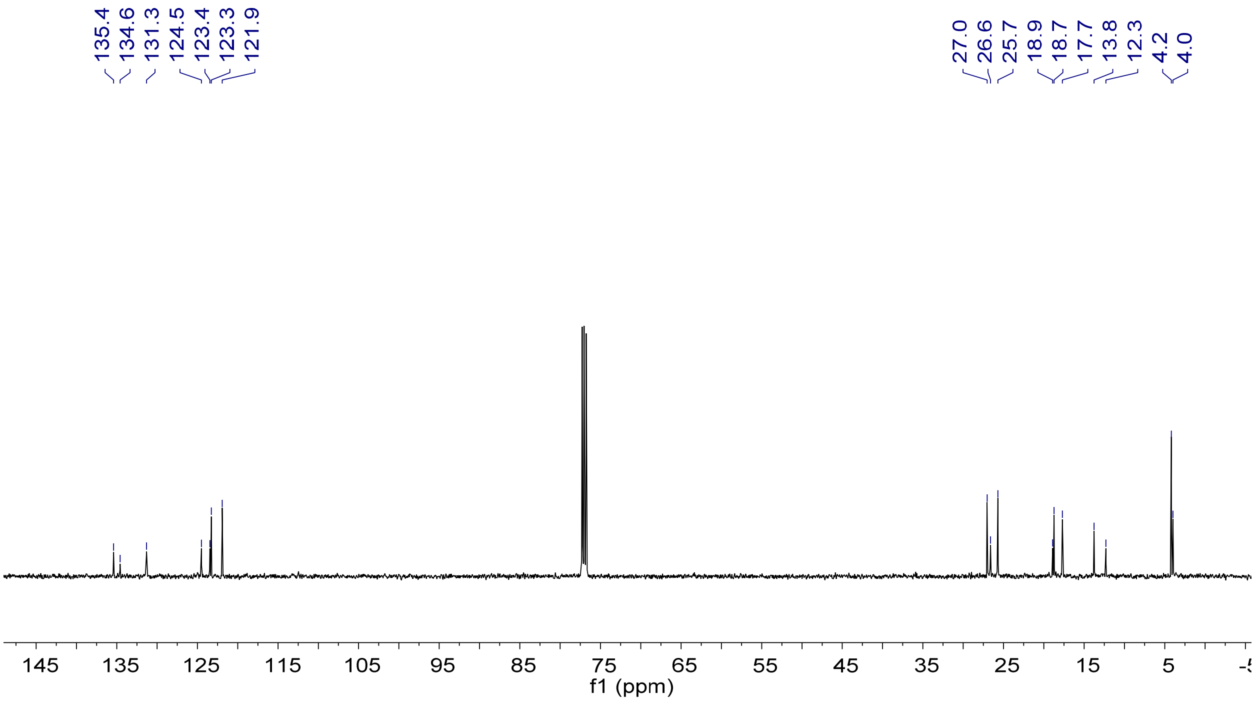

Figure S33. ^{13}C NMR spectrum for **16** ($CDCl_3$, 295 K).

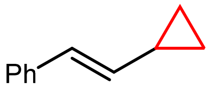

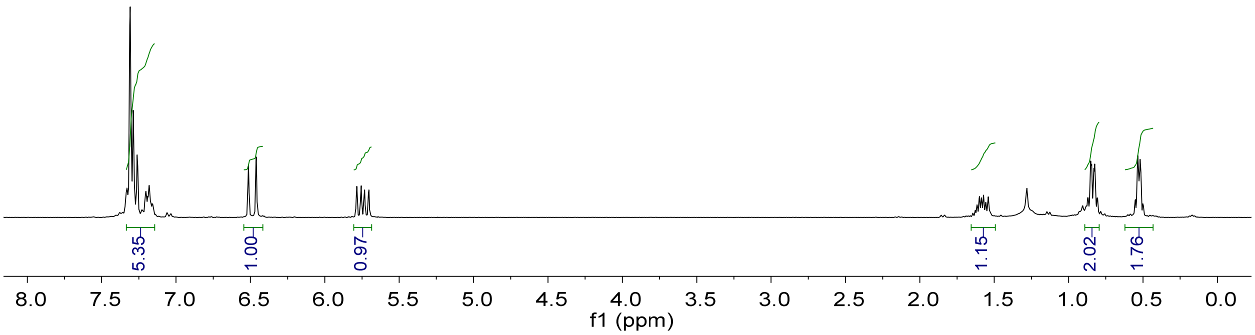

Figure S34. ^{1}H NMR spectrum for **17** ($CDCl_3$, 295 K).

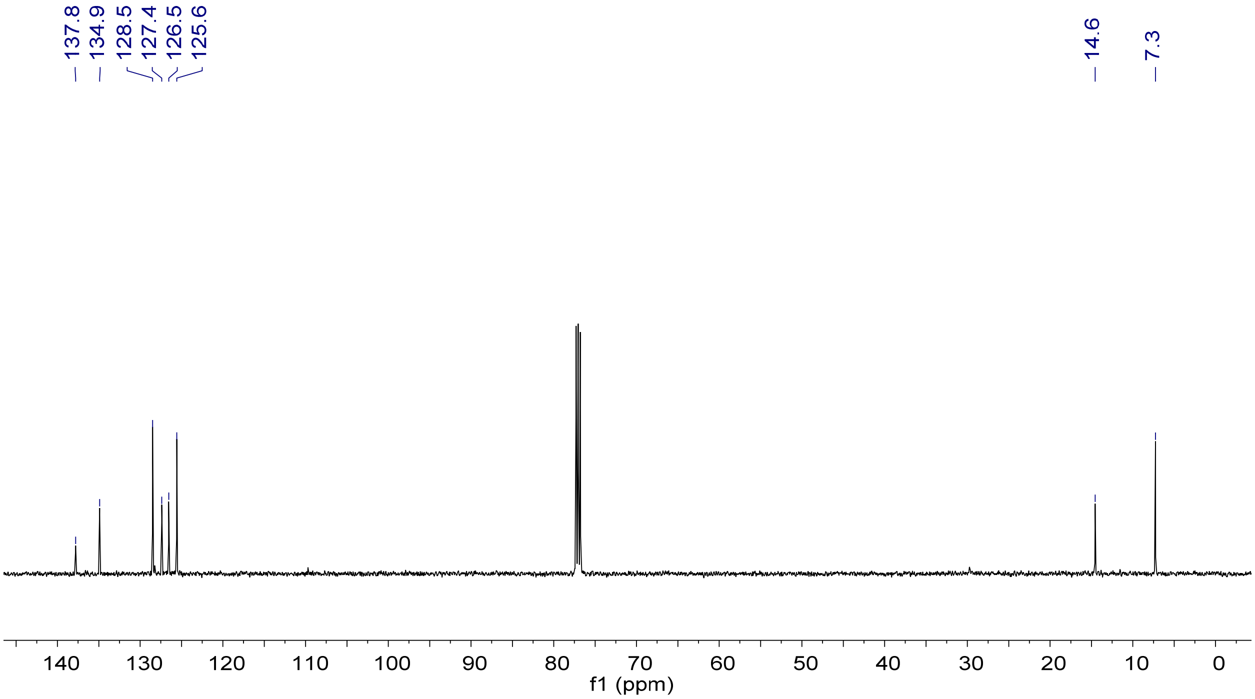

Figure S35. ^{13}C NMR spectrum for **17** ($CDCl_3$, 295 K)

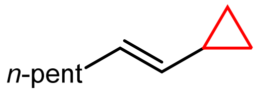

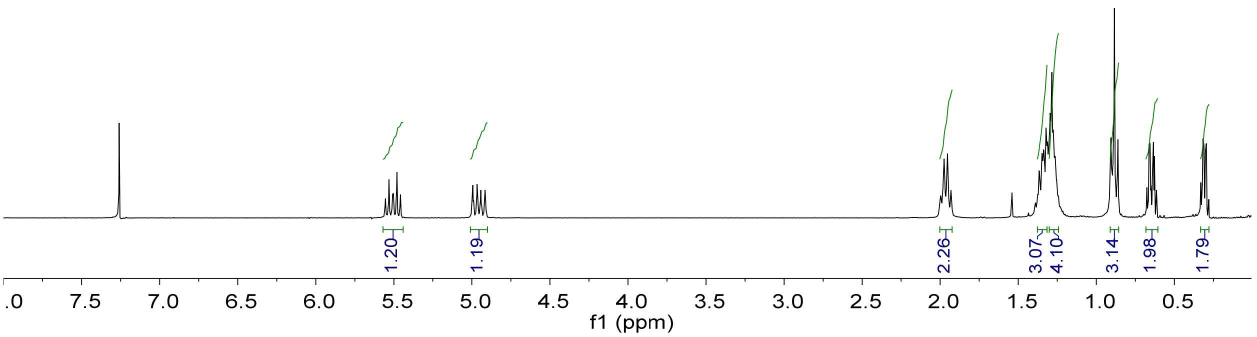

Figure S36. ^{1}H NMR spectrum for **18** ($CDCl_3$, 295 K)

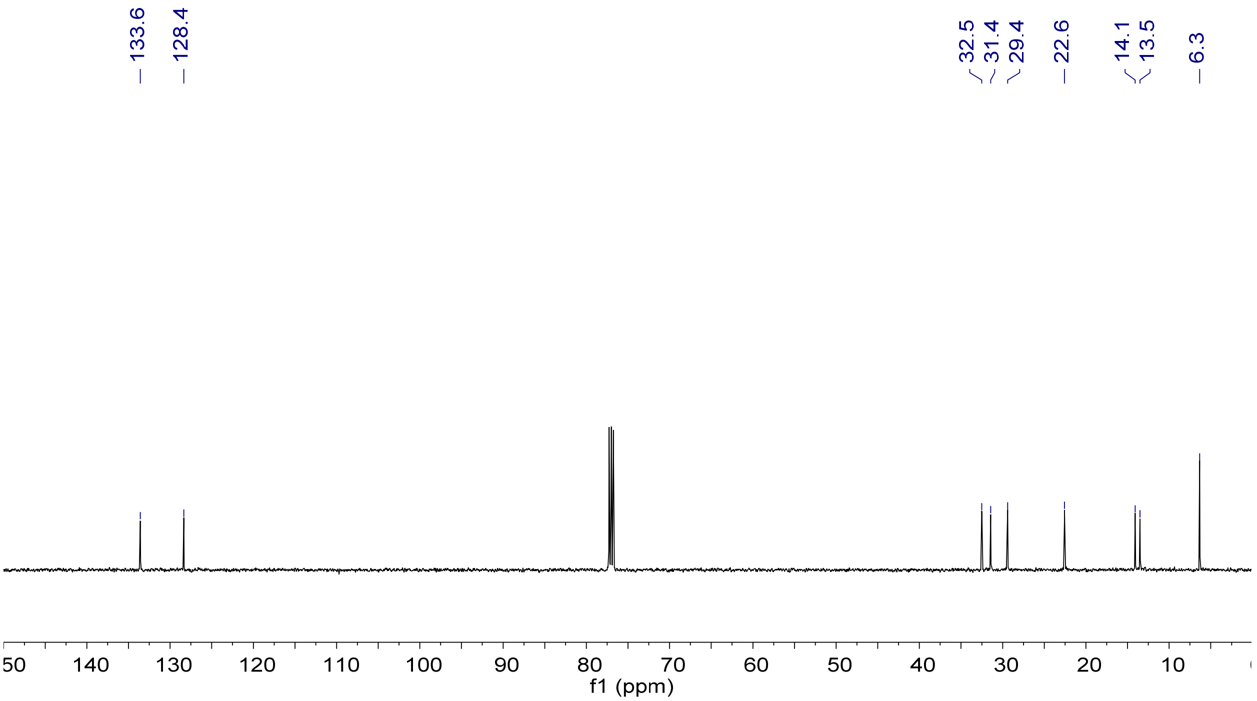

Figure S37. ^{13}C NMR spectrum for **18** ($CDCl_3$, 295 K)

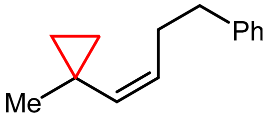

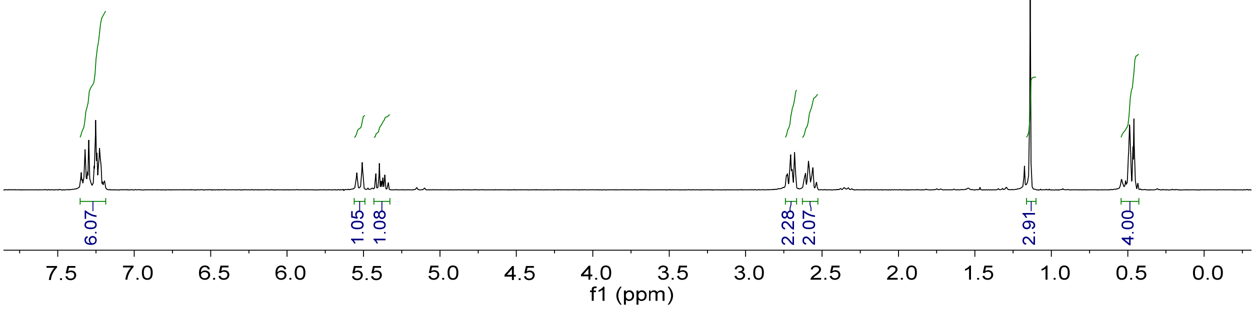

Figure S38. ^{1}H NMR spectrum for **19** ($CDCl_3$, 295 K)

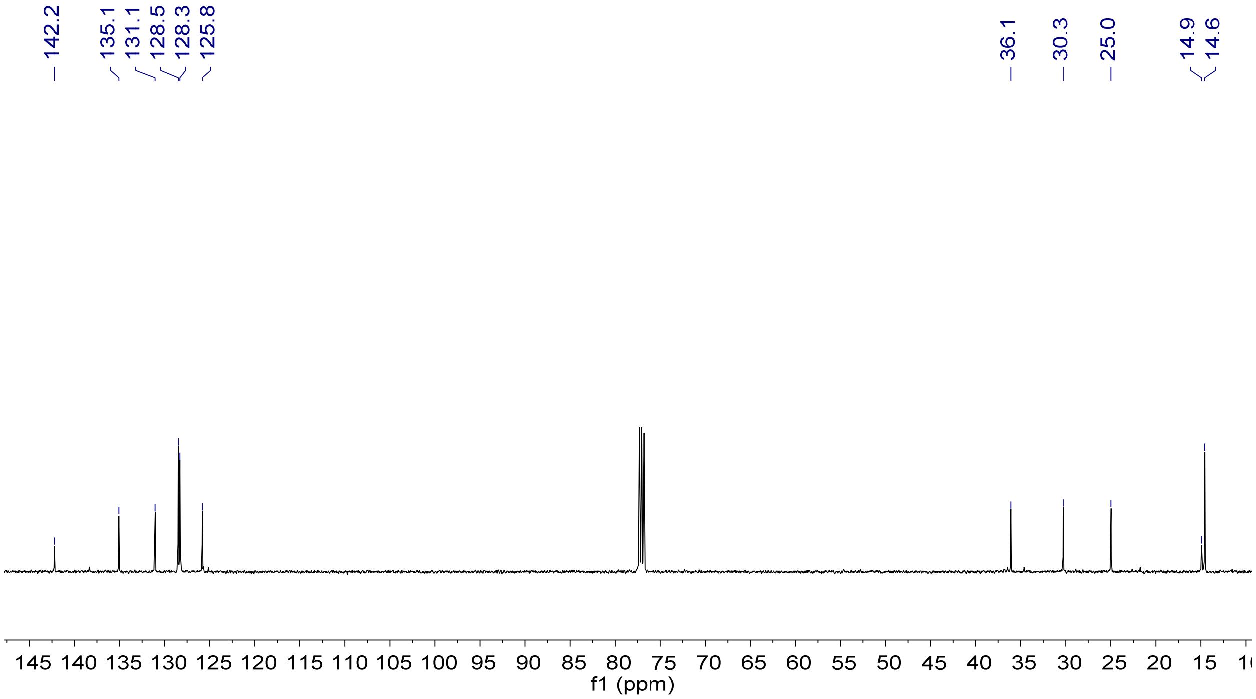

Figure S39. ^{13}C NMR spectrum for **19** ($CDCl_3$, 295 K)

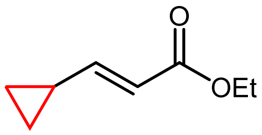

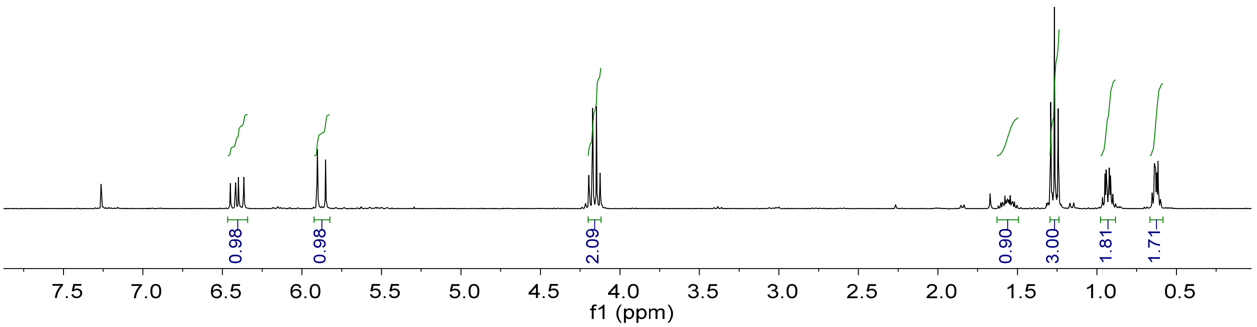

Figure S40. ^{1}H NMR spectrum for **20** ($CDCl_3$, 295 K)

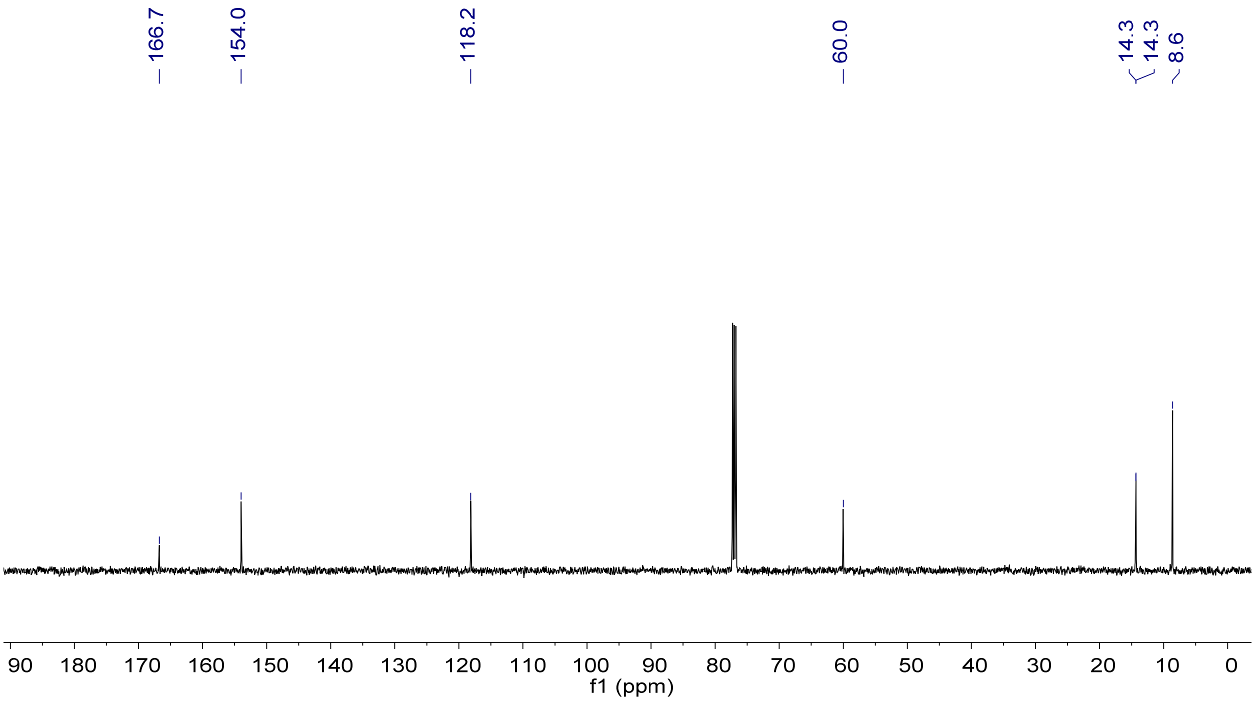

Figure S41. ^{13}C NMR spectrum for **20** ($CDCl_3$, 295 K)

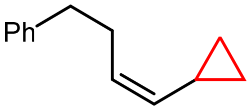

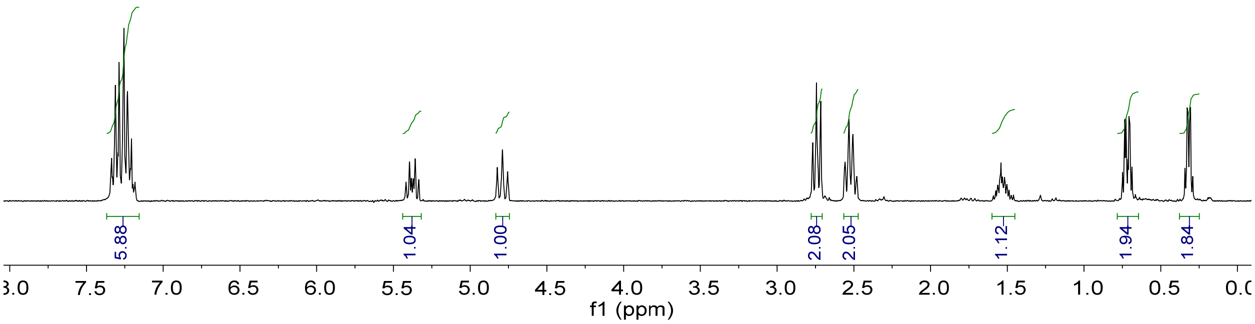

Figure S42. ^{1}H NMR spectrum for **21** ($CDCl_3$, 295 K)

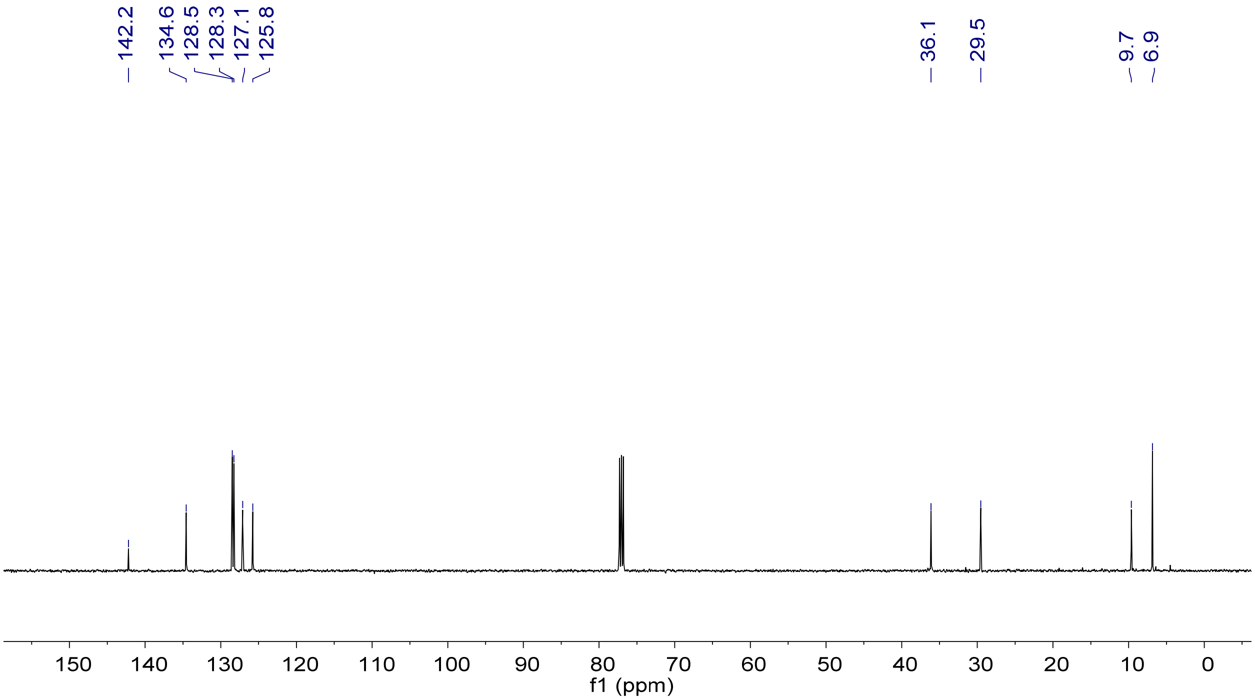

Figure S43. ^{13}C NMR spectrum for **21** ($CDCl_3$, 295 K)

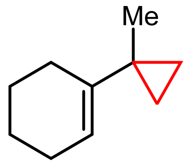

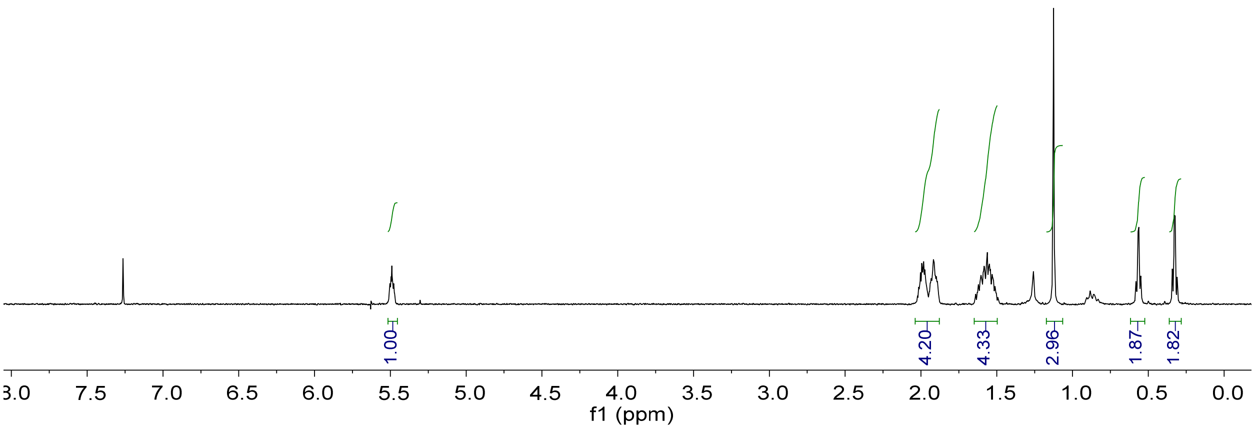

Figure S44. ^{1}H NMR spectrum for **22** ($CDCl_3$, 295 K)

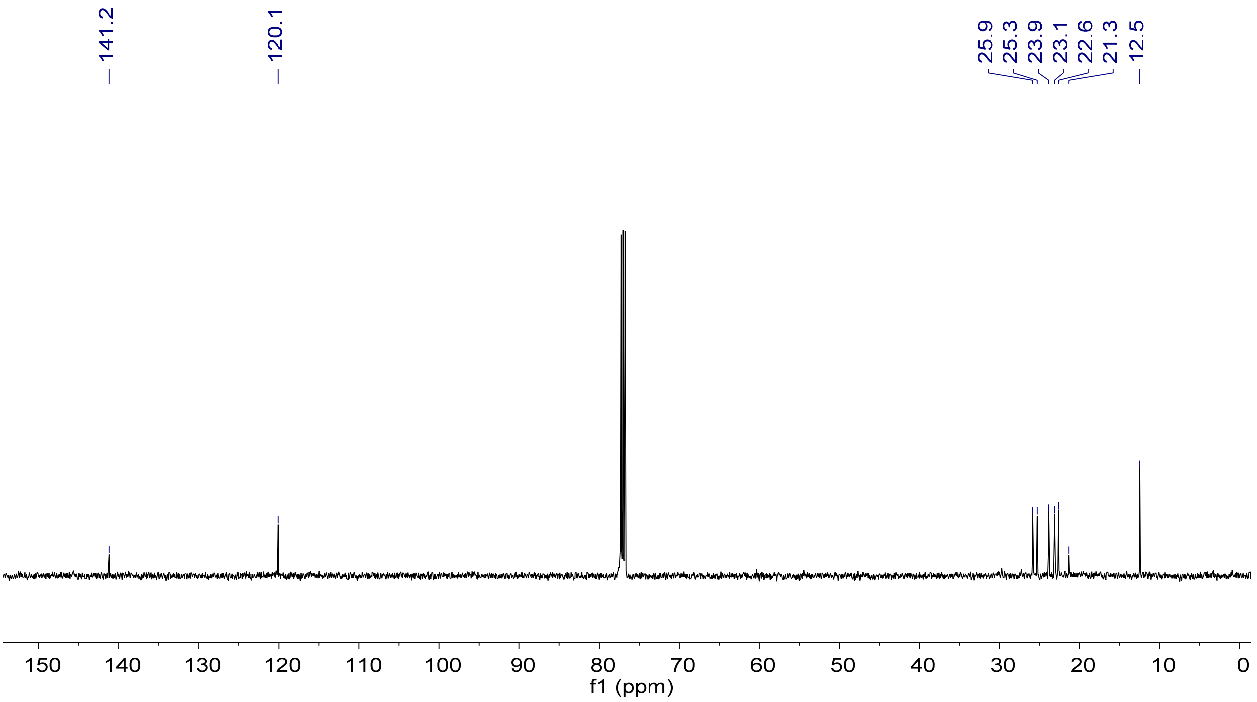

Figure S45. ^{13}C NMR spectrum for **22** ($CDCl_3$, 295 K)

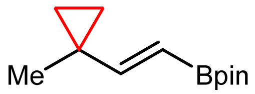

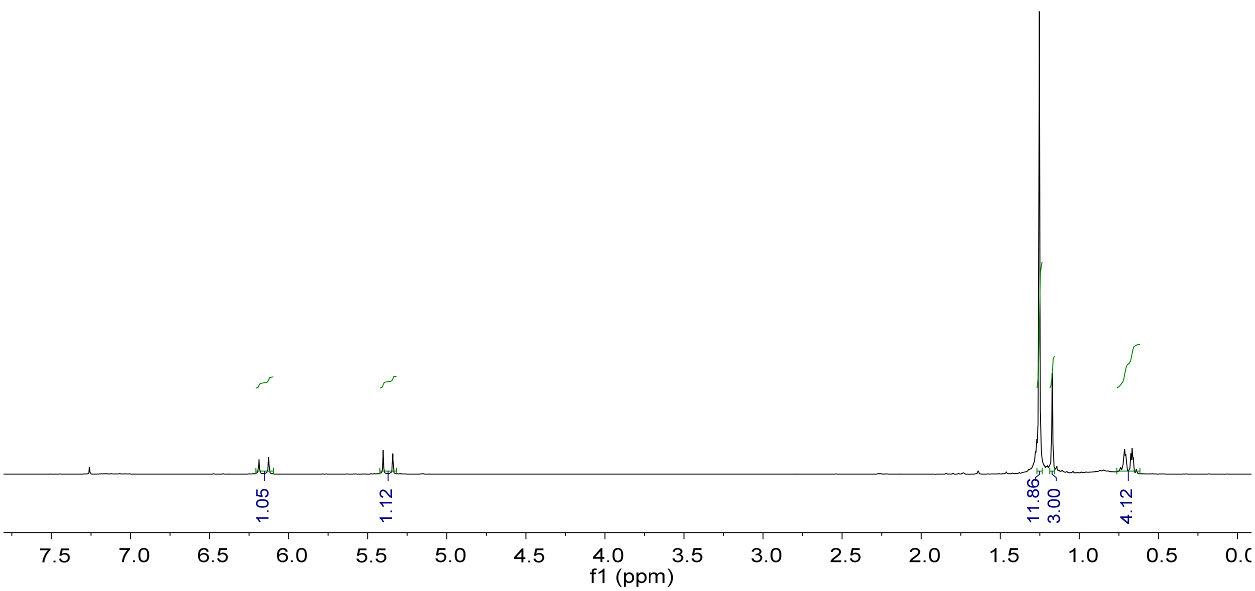

Figure S46. ^{1}H NMR spectrum for **23** ($CDCl_3$, 295 K)

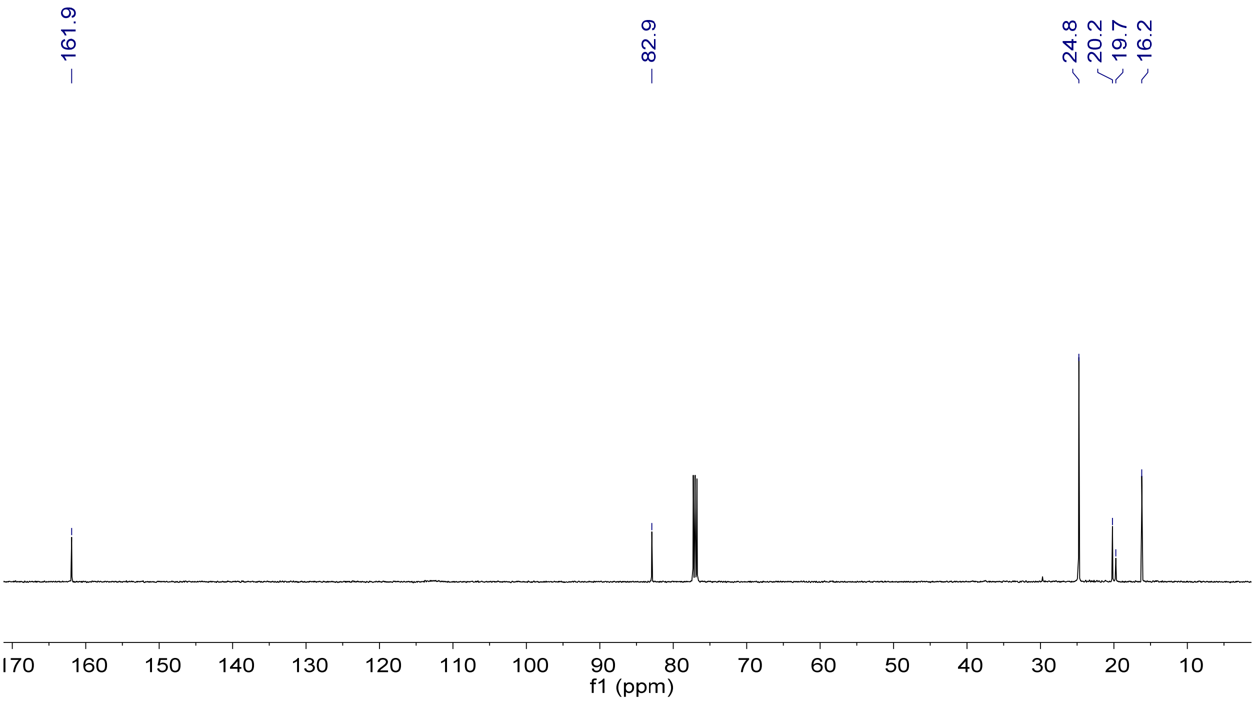

Figure S47. ^{13}C NMR spectrum for **23** ($CDCl_3$, 295 K)

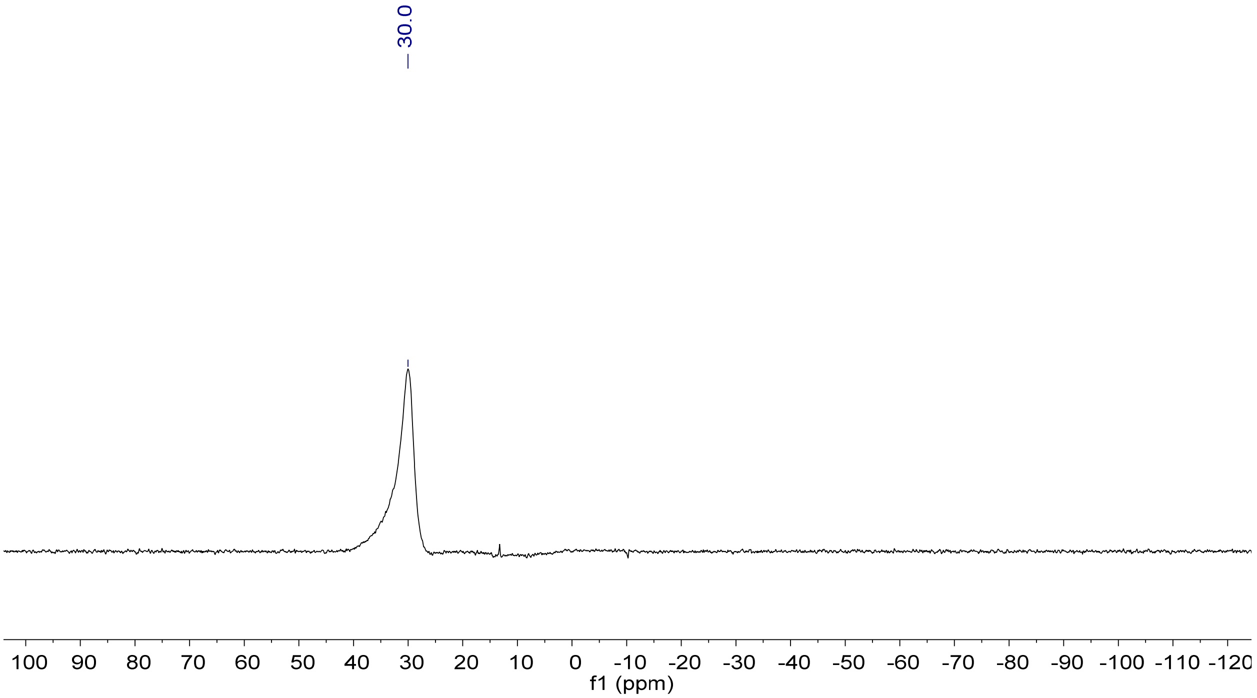

Figure S48. ^{11}B NMR spectrum for **23** ($CDCl_3$, 295 K)

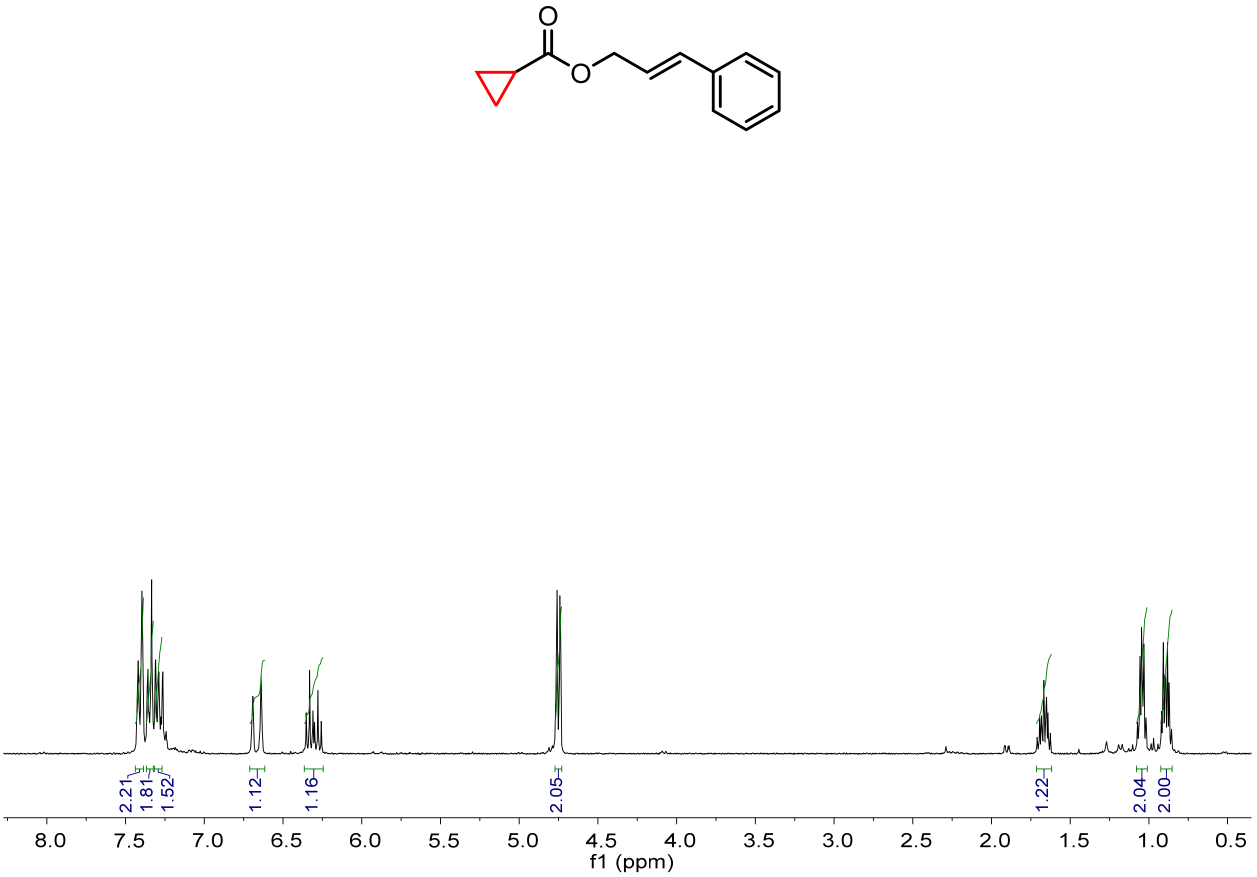

Figure S49. ^{1}H NMR spectrum for **S1** ($CDCl_3$, 295 K)

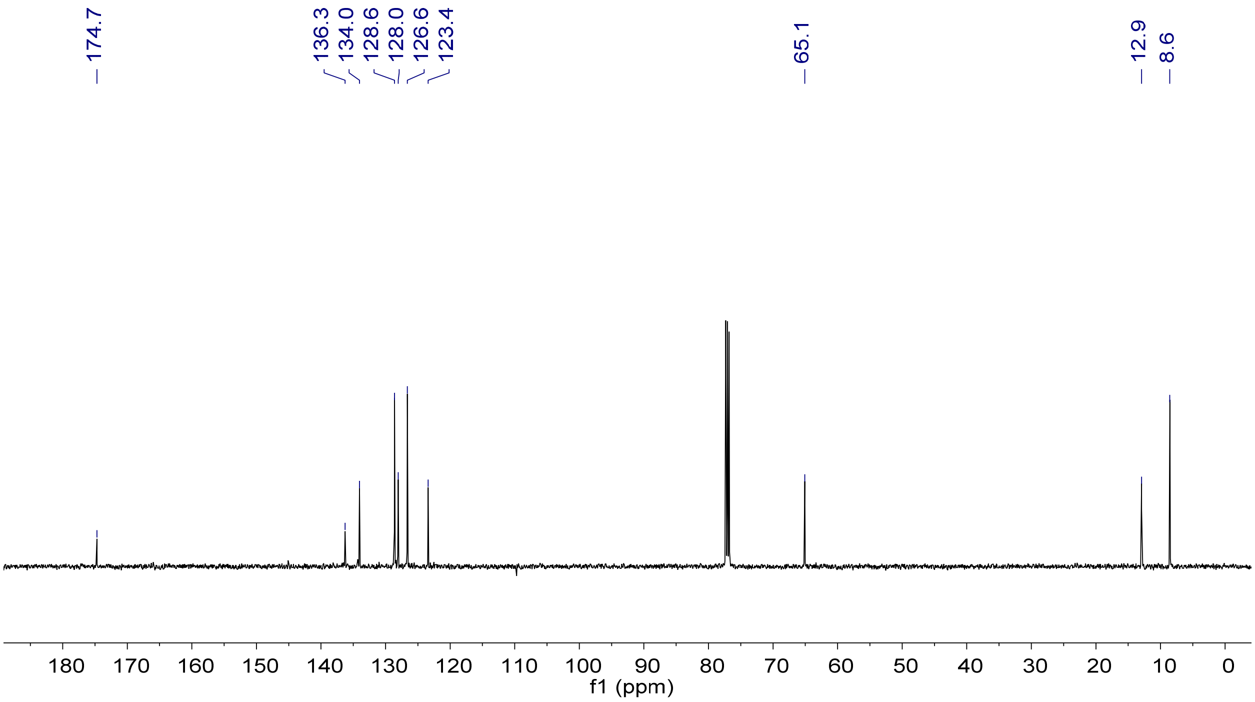

Figure S50. ^{13}C NMR spectrum for **S1** ($CDCl_3$, 295 K)

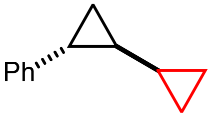

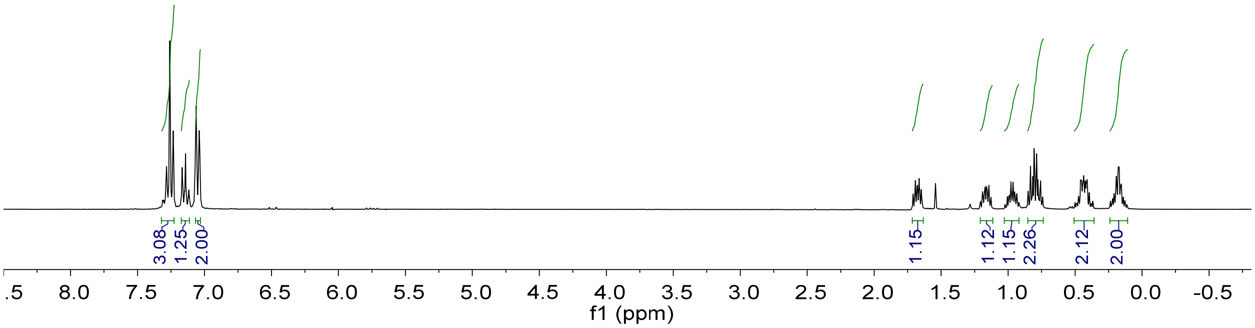

Figure S51. ^{1}H NMR spectrum for **29** ($CDCl_3$, 295 K)

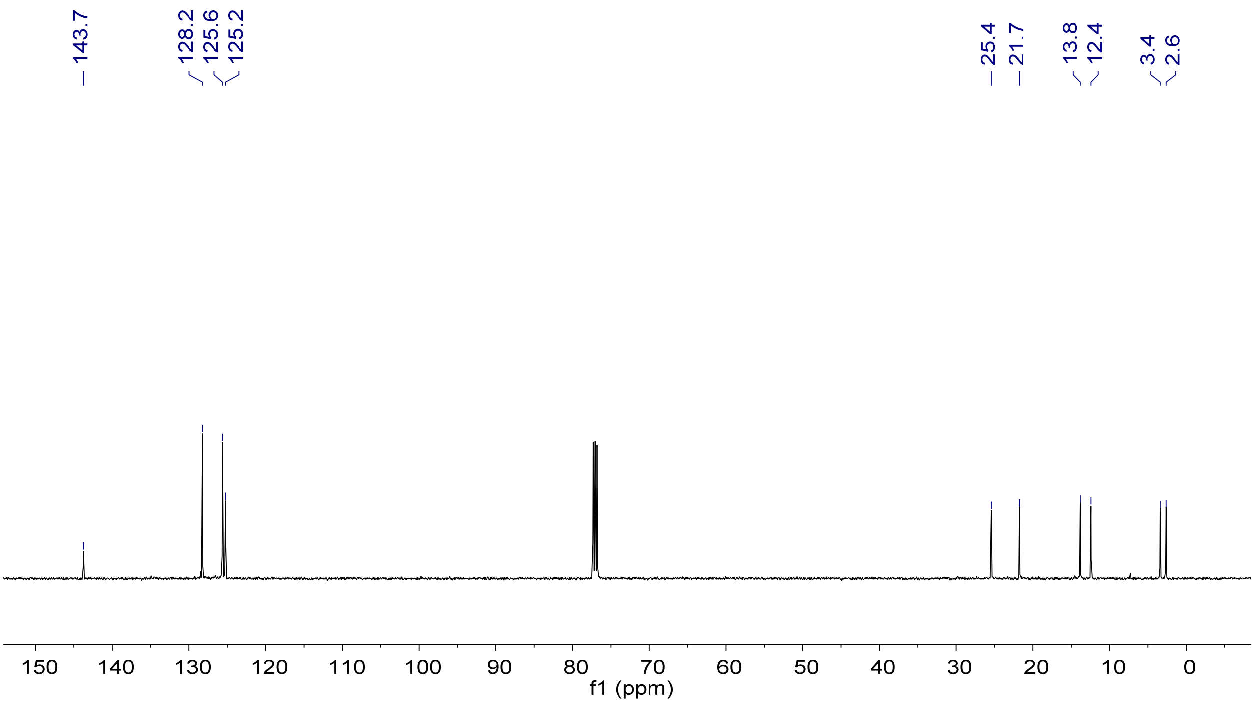

Figure S52. ^{13}C NMR spectrum for **29** ($CDCl_3$, 295 K)

11. IR Spectra

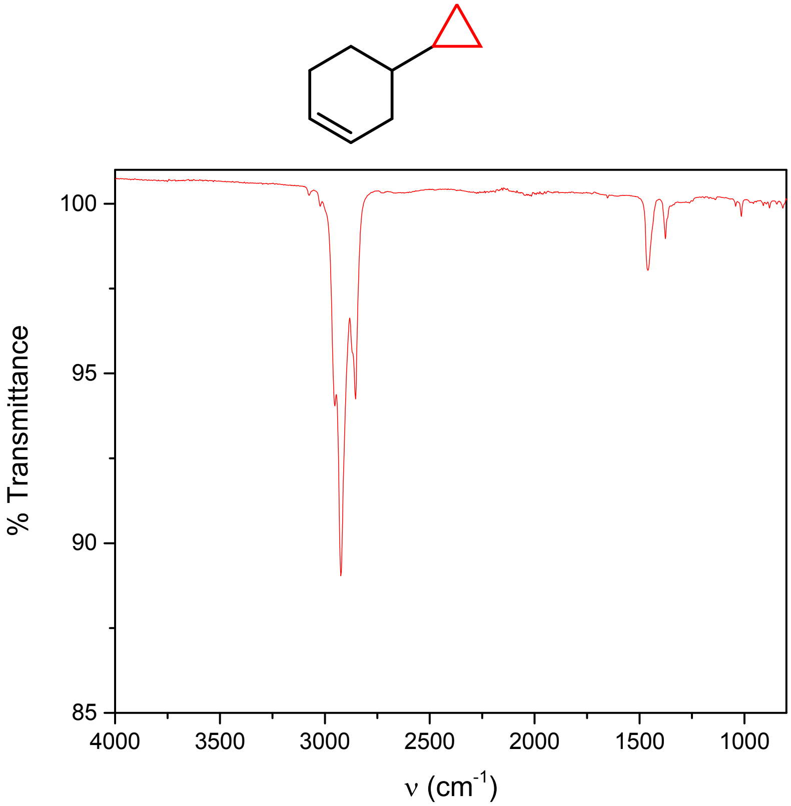

Figure S53. ATR-IR spectrum for **5**.

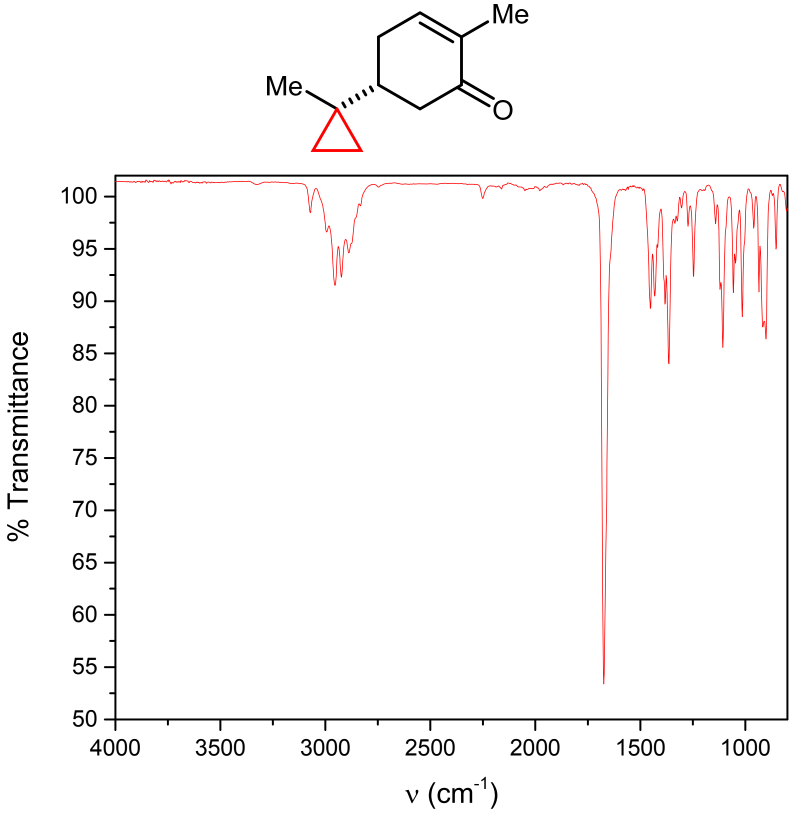

Figure S54. ATR-IR spectrum for **6**.

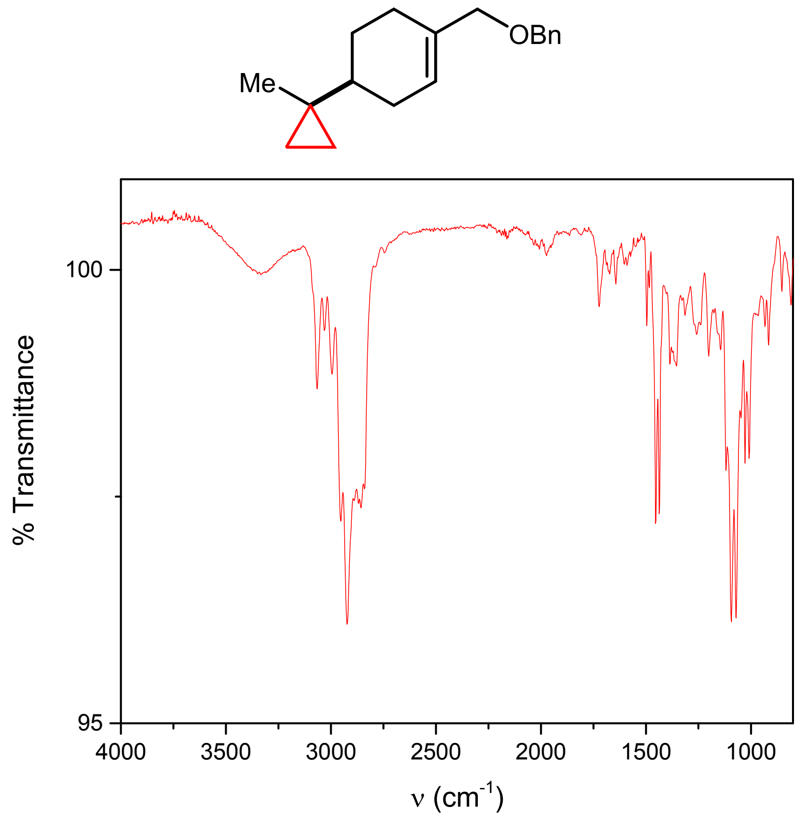

Figure S55. ATR-IR spectrum for **7**.

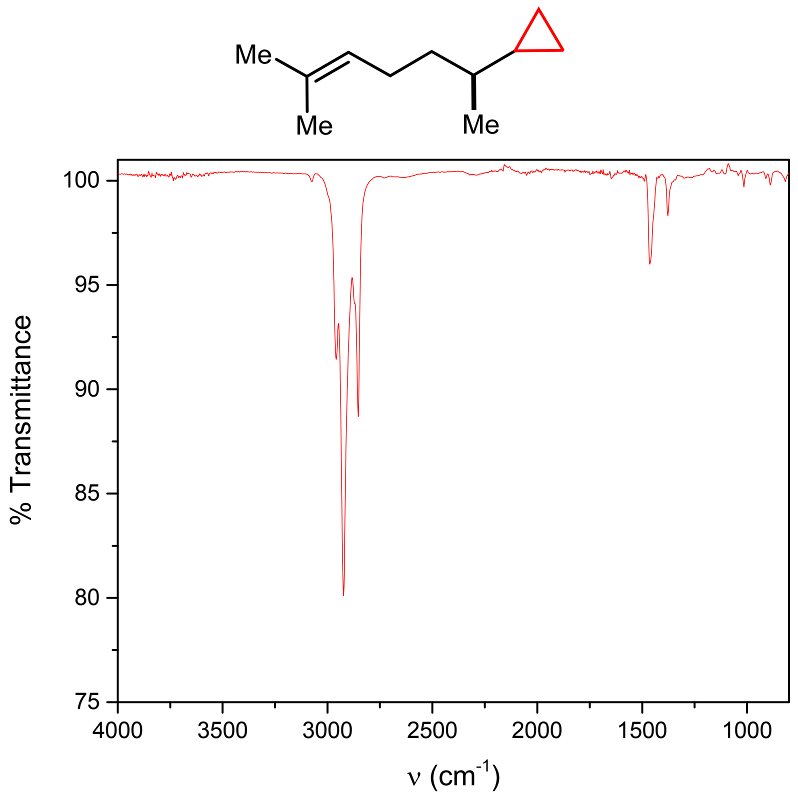

Figure S56. ATR-IR spectrum for **8**.

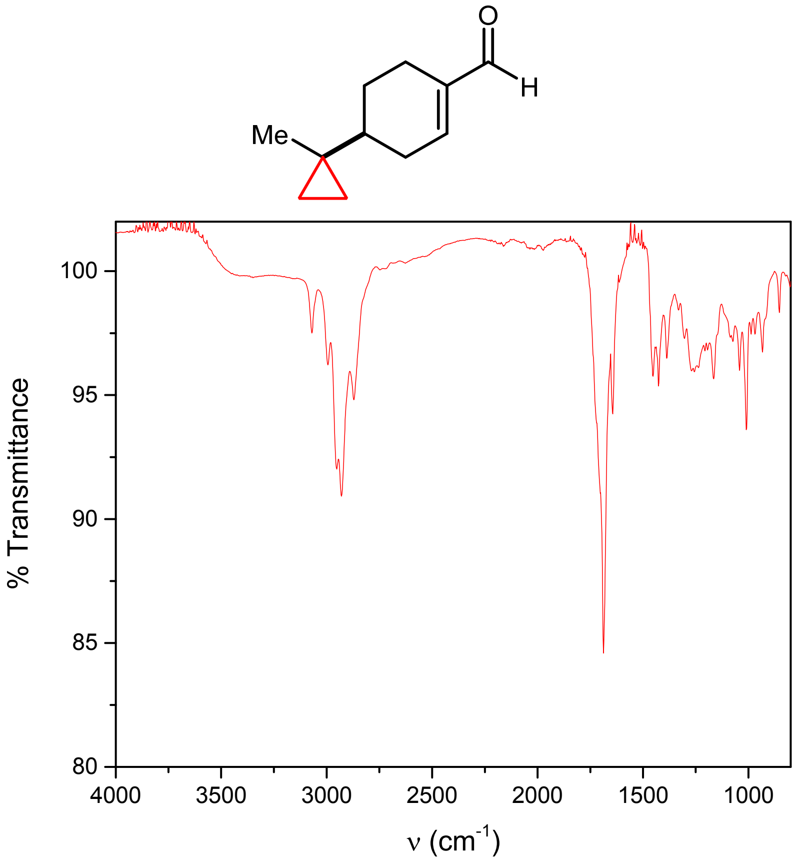

Figure S567. ATR-IR spectrum for **9**.

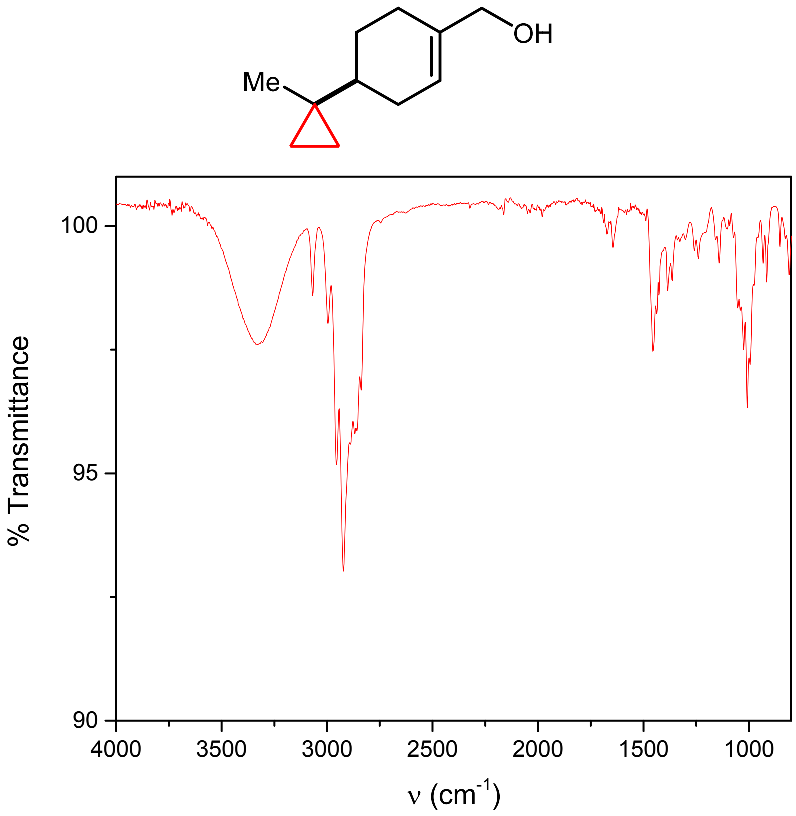

Figure S58. ATR-IR spectrum for **10**.

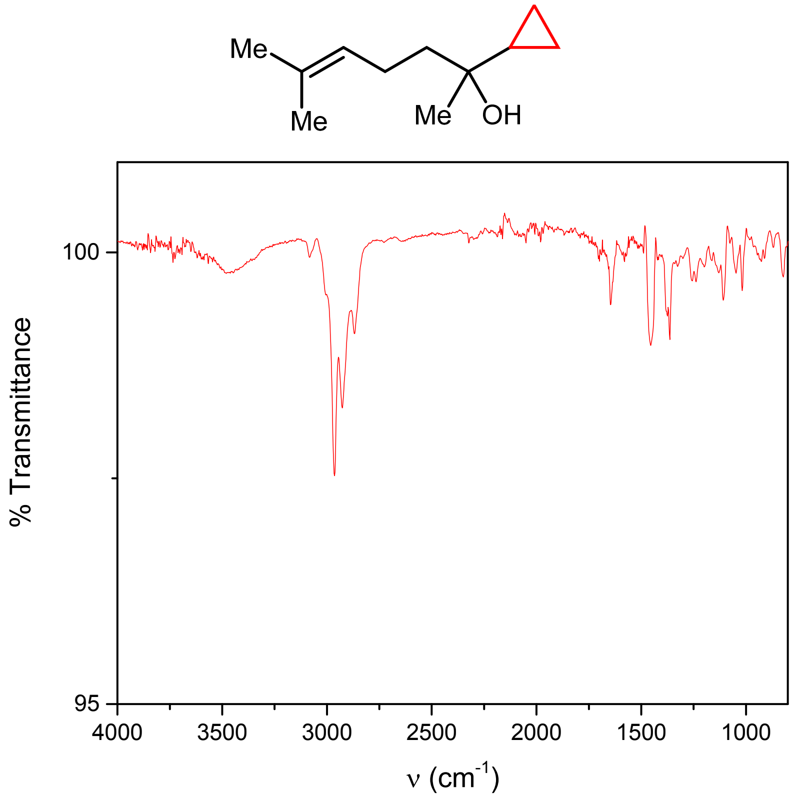

Figure S59. ATR-IR spectrum for **11**.

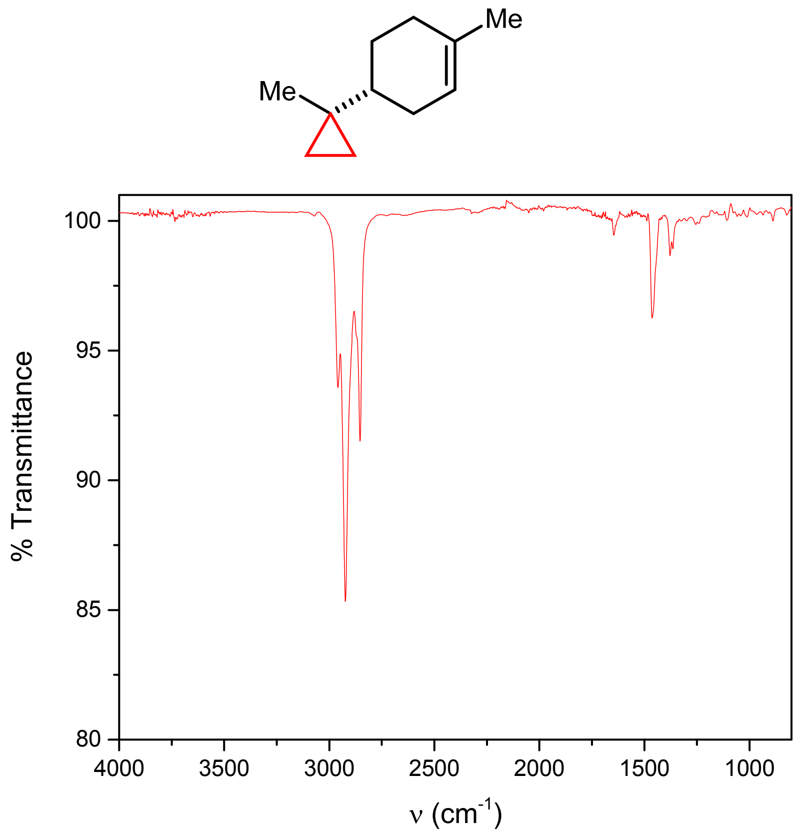

Figure S60. ATR-IR spectrum for **12**.

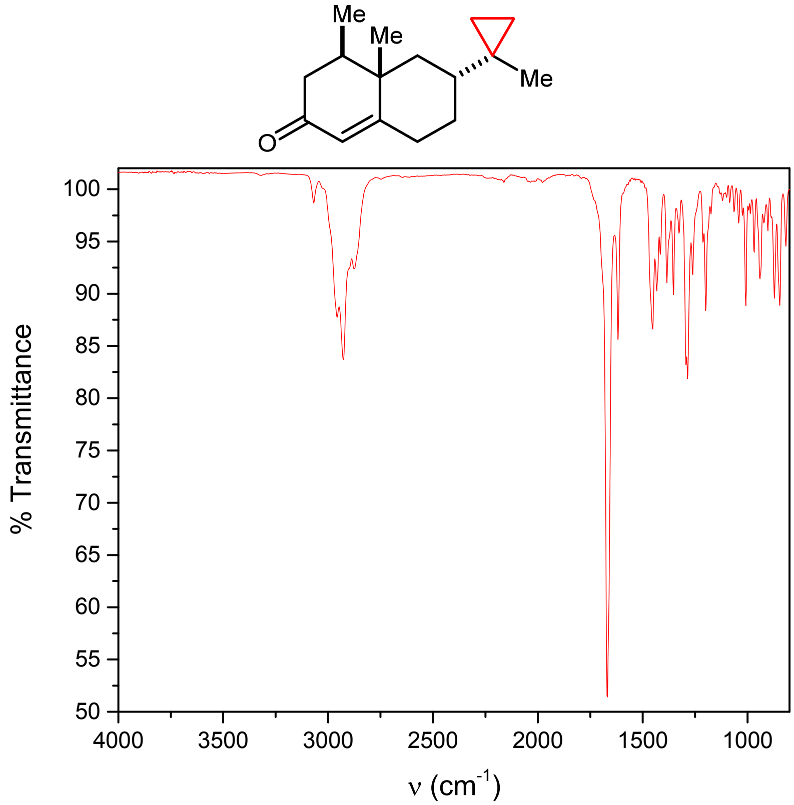

Figure S61. ATR-IR spectrum for **13**.

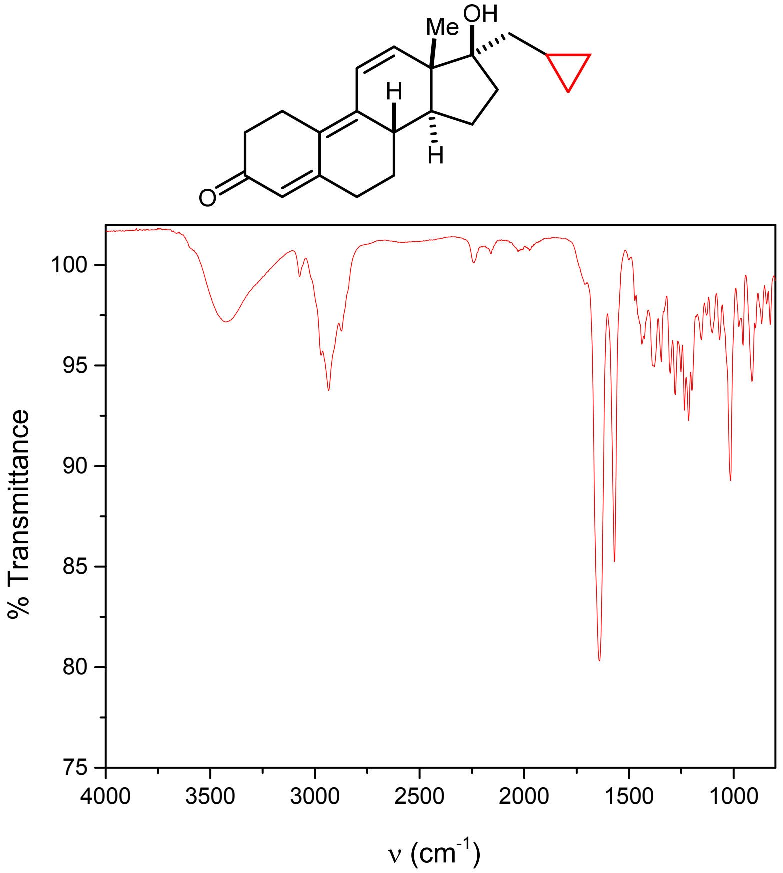

Figure S62. ATR-IR spectrum for **14**.

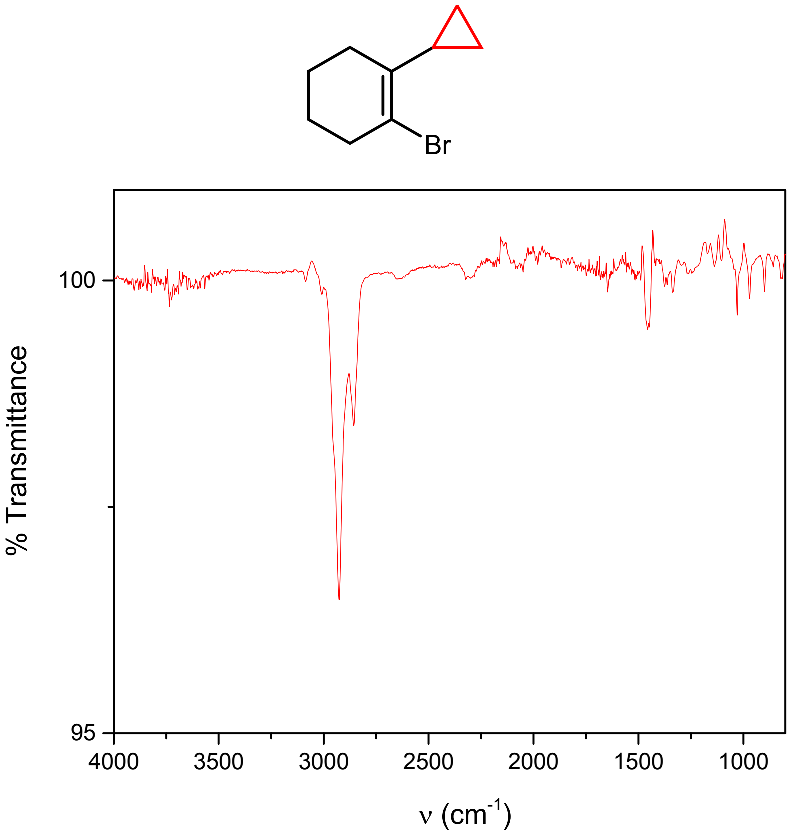

Figure S63. ATR-IR spectrum for **15**.

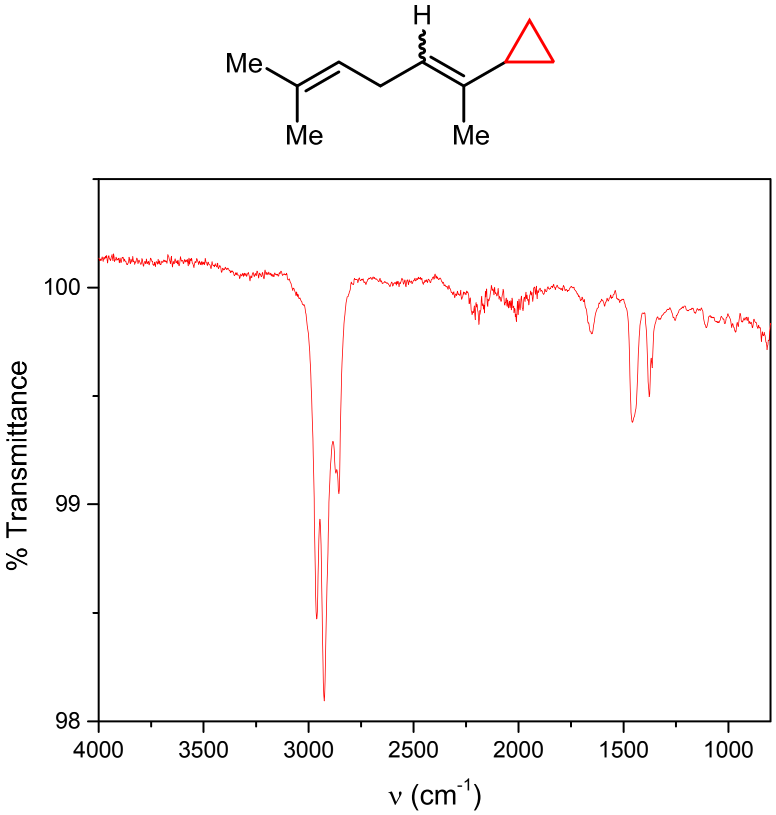

Figure S64. ATR-IR spectrum for **16**.

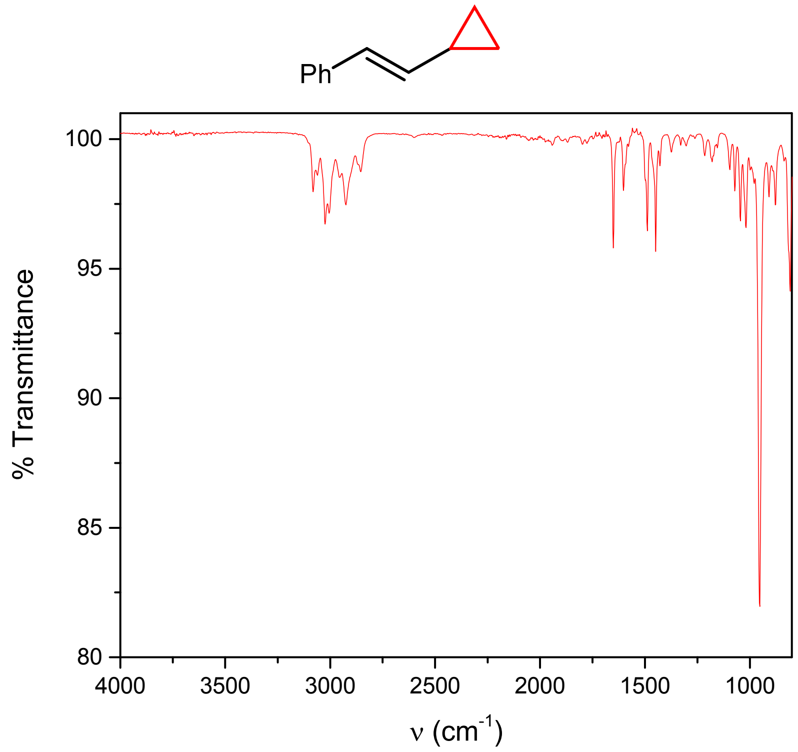

Figure S65. ATR-IR spectrum for **17**.

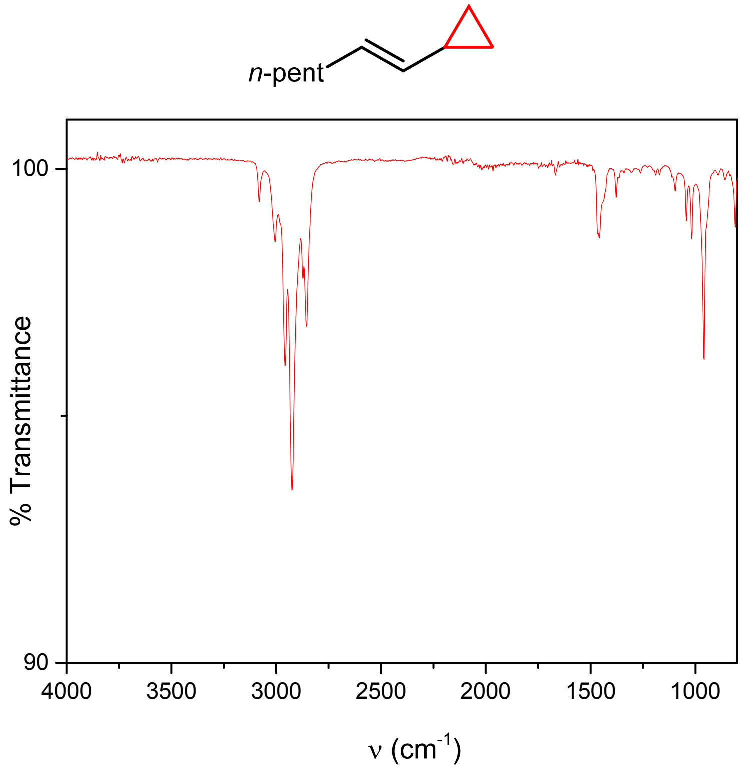

Figure S66. ATR-IR spectrum for **18**.

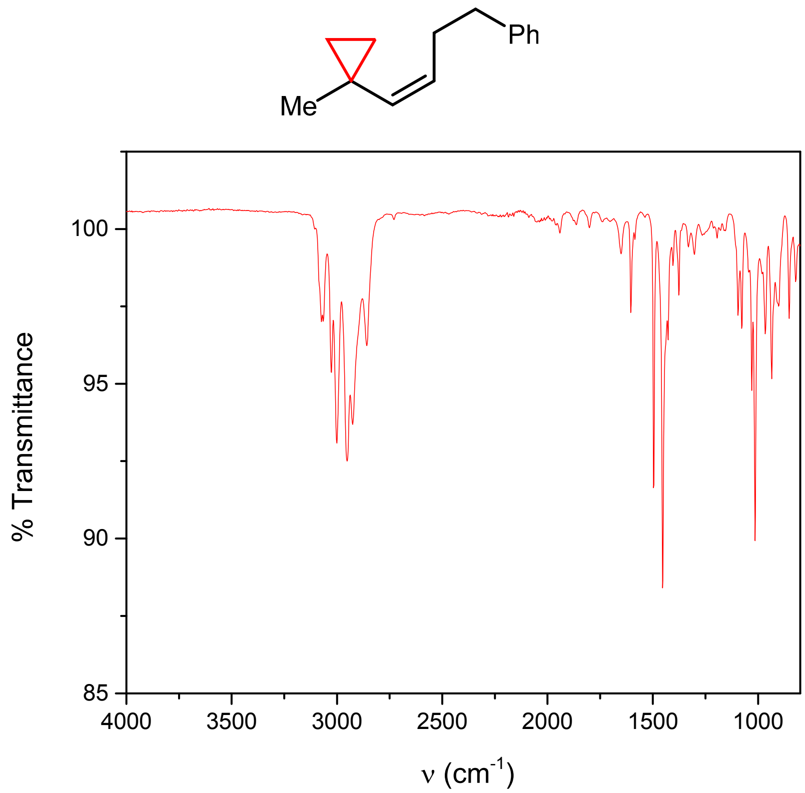

Figure S67. ATR-IR spectrum for **19**.

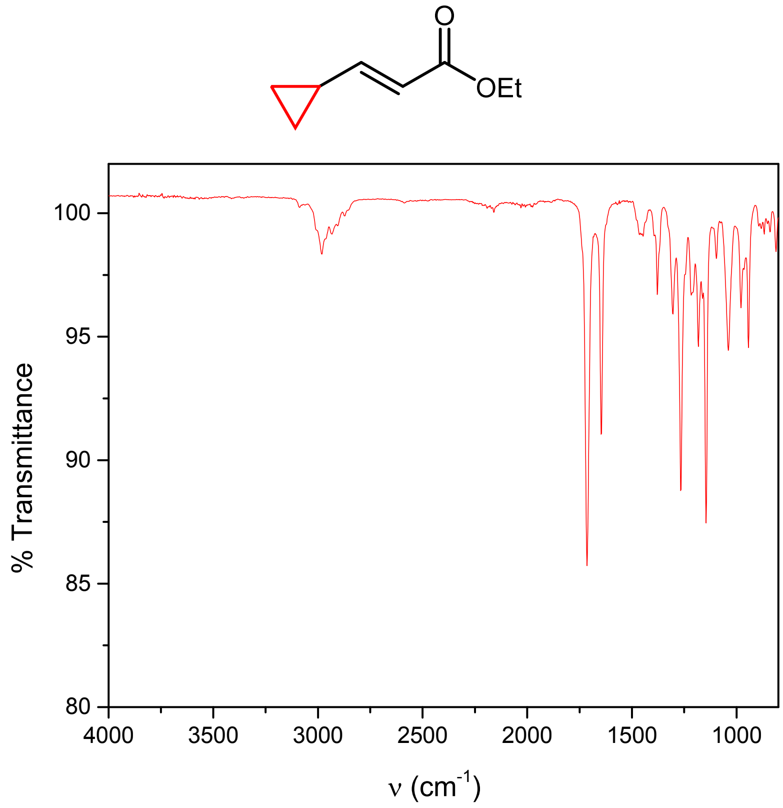

Figure S68. ATR-IR spectrum for **20**.

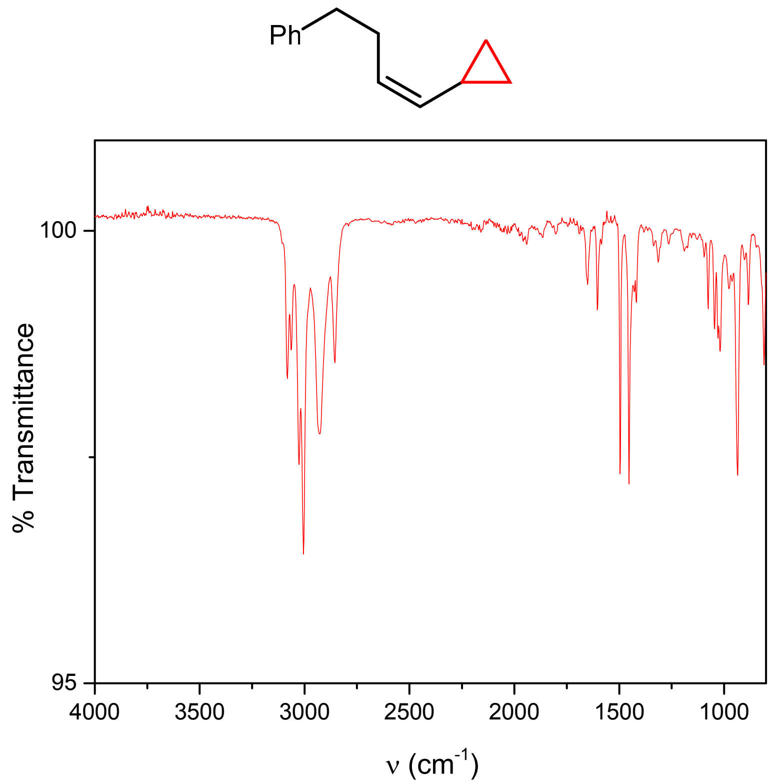

Figure S69. ATR-IR spectrum for **21**.

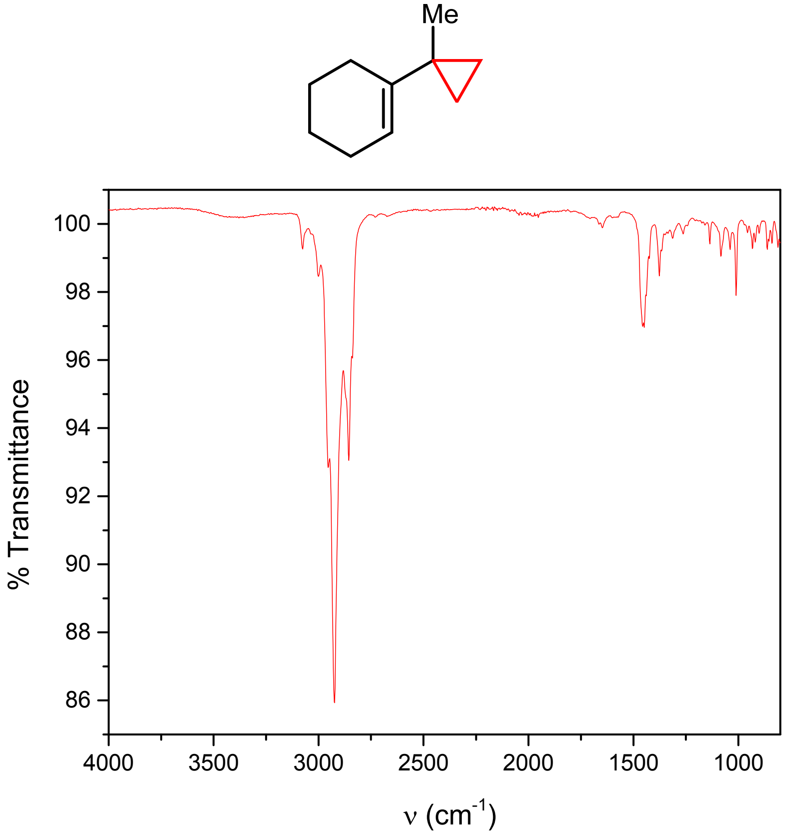

Figure S70. ATR-IR spectrum for **22**.

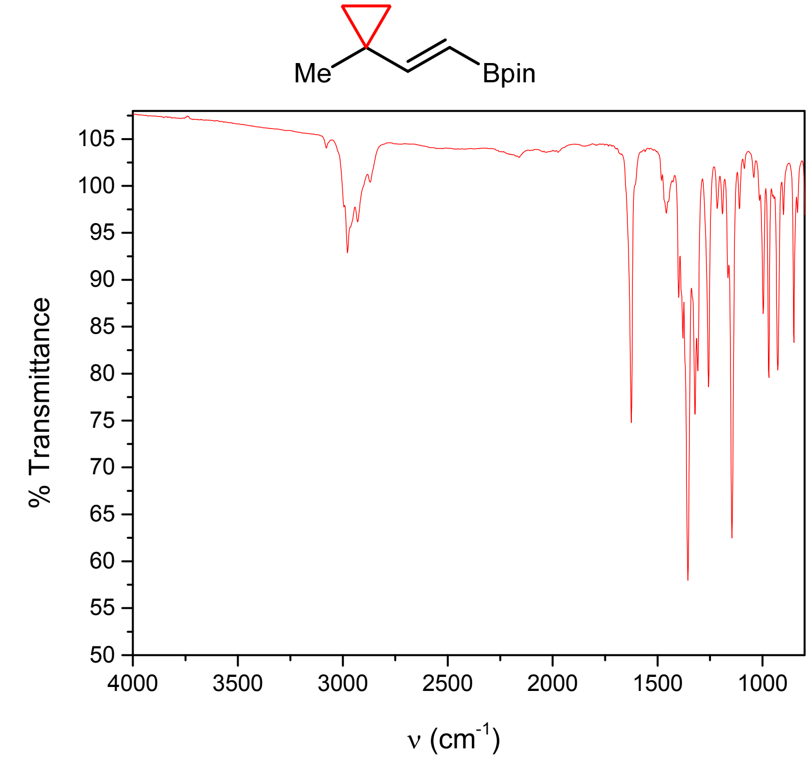

Figure S71. ATR-IR spectrum for **23**.

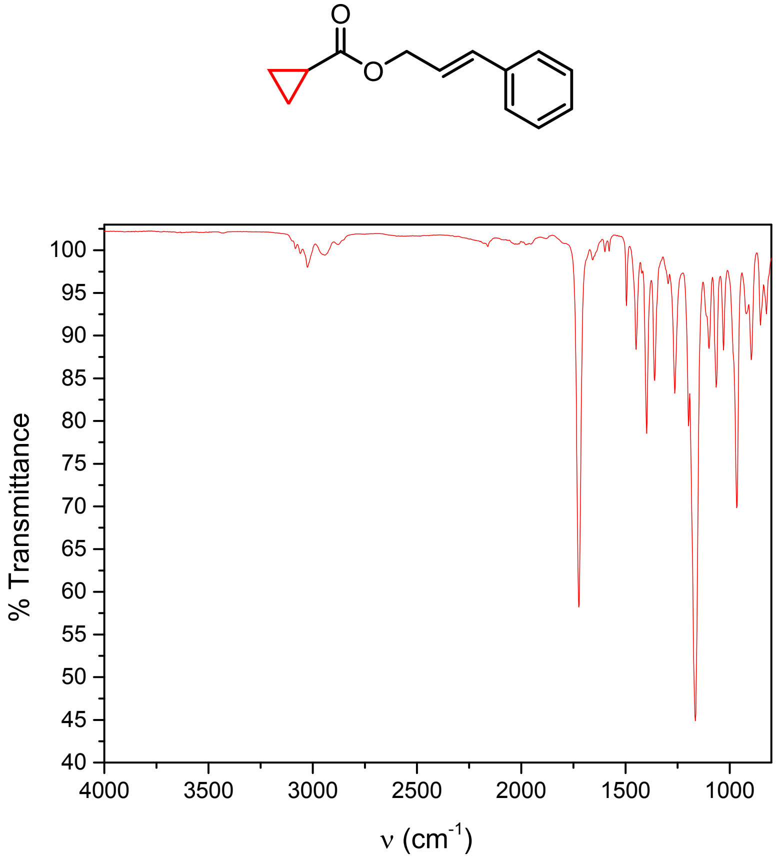

Figure S72. ATR-IR spectrum for **S1**.

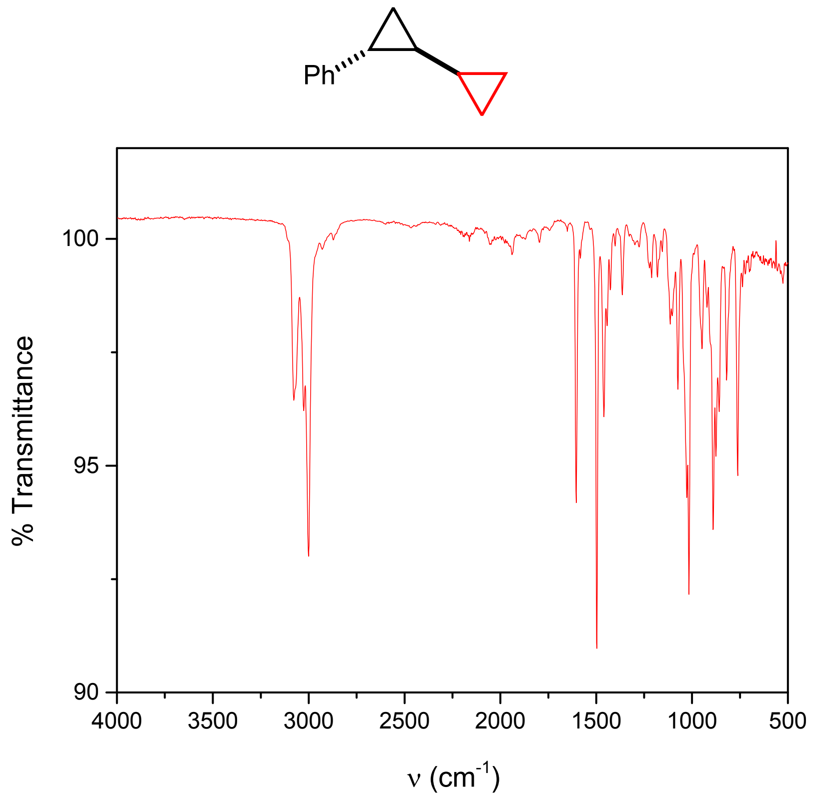

Figure S73. ATR-IR spectrum for **29**.

References

[1] Wei, L.; Yang, Y.; Fan, R.; Wang, P.; Li, L.; Yu, J.; Yang, B.; Cao, W. *RSC. Adv*. **2013**. 3, 25908-25916.
[2] A. M. A. Bennett (DuPont), *WO Pat*., 98/27124, 1998
[3] Bruker (2016). Apex3 v2016.9-0, Saint V8.34A, SAINT V8.37A, Bruker AXS Inc.: Madison (WI), USA, 2013/2014.
[4] (a) SHELXTL suite of programs, Version 6.14, 2000-2003, Bruker Advanced X-ray Solutions, Bruker AXS Inc., Madison, Wisconsin: USA; (b) Sheldrick, G. M. *Acta Crystallogr A.* **2008**, *64*, 112–122.
[5] Sheldrick, G. M. *Acta Crystallogr Sect C Struct Chem.* **2015**, *71*, 3–8.
[6] Hübschle, C. B.; Sheldrick, G. M.; Dittrich, B. *J. Appl. Crystallogr.* **2011**, *44*, 1281–1284.
[7] Arora, A.; Teegardin, K.A.; Weaver, J.D. *Org. Lett*. **2015**, 17, 3722-3725.
[8] Percy, J.M.; Emerson,H.; Fyfe, J.W.B.; Kennedy, A.R.; Maciuk, S.; Orr, D.; Rathouská, L.; Redmond, J.M.; Wilson, P.G. *Chem. Eur. J.* **2016**, 22, 12166-12175.
[9] Zheng, M.; Huang, L.; Wu, W.; Jiang, H. *Org. Lett.* **2013**, 15, 1838-1841.
[10] Tamura, R.; Saegusa, K.; Kakihana, M.; Oda, D. *J. Org. Chem.* **1988**, 53, 2723-2728.
[11] Zhao, D.; Lied, F.; Glorius, F. *Chem. Sci*. **2014**, 5, 2869.
[12] Kilman, L.T.; Mlynarski, S. N.; Ferris, G. E.; Morken, J. P. *Angew. Chem. Int. Ed*. **2012**, 51, 521-524.
[13] Niu, G.; Hou, C.; Chuang, G.J.; Wu, C.; Liao, C. *Eur. J. Org. Chem*. **2014**, 3794-3801.
[14] Cannillo, A.; Norsikian, S.; Retailleau, P.; Dau, M.T.H.; Iorga, B.I.; Beau, J. *Chem. Eur. J*. **2013**, 19, 9127-9131.
[15] Kalyva, M.; Zografos, A.L.; Kapourani, E.; Giambazolias, E.; Devel, L.; Papakyriakou, A.; Dive, V.; Lazarou, Y.G.; Georgiadis, D. *Chem. Eur. J.* **2015**, 21, 3278-3289.
[16] Coombs, J. R.; Haeffner, F.; Kilman, L.T.; Morken, J. P. *J. Am. Chem. Soc*. **2013**, 135, 11222-11231.
[17] Jang, Y.H.; Youn, S.W. *Org. Lett*., **2014**, 16, 3720-3723.
